# Supplementary material for: ZNF117 regulates glioblastoma stem cell differentiation towards oligodendroglial lineage
Source: Nat Commun. 2022 Apr 22;13:2196. doi: 10.1038/s41467-022-29884-3 (PMC9033827; doi:10.1038/s41467-022-29884-3)
Supplement: Supplementary file 1 — Supplementary Information [file 41467_2022_29884_MOESM1_ESM.pdf]

SUPPLEMENTARY INFORMATION

ZNF117 regulates glioblastoma stem cell differentiation towards oligodendroglial lineage

Jun Liu[1#], Xiaoying Wang[1,2#], Ann T. Chen[1,3#], Xingchun Gao[1#], Benjamin T. Himes[3], Hongyi Zhang[1], Zeming Chen[1], Jianhui Wang[4], Wendy C. Sheu[1,3], Gang Deng[1], Yang Xiao[3], Pan Zou[1], Shenqi Zhang[1], Fuyao Liu[1], Yong Zhu[5], Rong Fan[3], Toral R. Patel[1,6*], W. Mark Saltzman[3*], Jiangbing Zhou[1,3 *]

[1]Department of Neurosurgery, [3]Department of Biomedical Engineering, [4]Department of Pathology, [5]School of Public Health, Yale University, New Haven, CT 06511, USA. [2]Wuxi School of Medicine, Jiangnan University, Jiangsu Province, 214122, China. [6]Department of Neurosurgery, University of Texas Southwestern Medical Center, Dallas, Texas, United States.

[#] These authors contributed equally to this work.

*Correspondence to e-mail:

jiangbing.zhou@yale.edu or mark.saltzman@yale.edu or toral.patel@utsouthwestern.edu

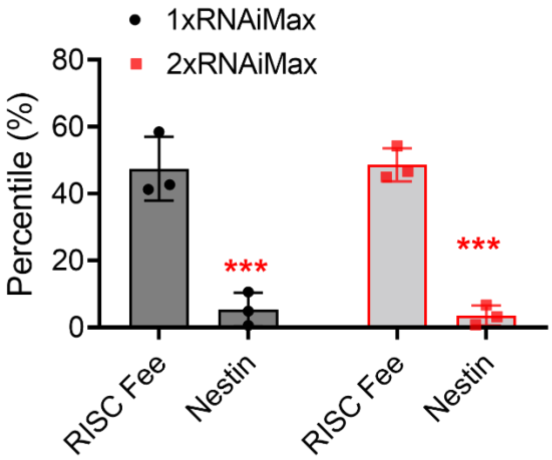

Extended Data Fig. 1. Quantification of the efficiency in reducing Nestin expression in GS5 cells by delivering siNestin or control siGENOME RISC-Free siRNA. 2x RNAiMax represents the use of two times volume of lipofectamine RNAiMax reagent suggested by the vendor (Invitrogen). 2x RNAiMax, which demonstrates a slightly greater efficiency than 1x RNAiMax, was selected for the genome wide RNAi screen. The mean of data of three biologically independent samples is shown and data are presented as mean values ± SD (n = 3).

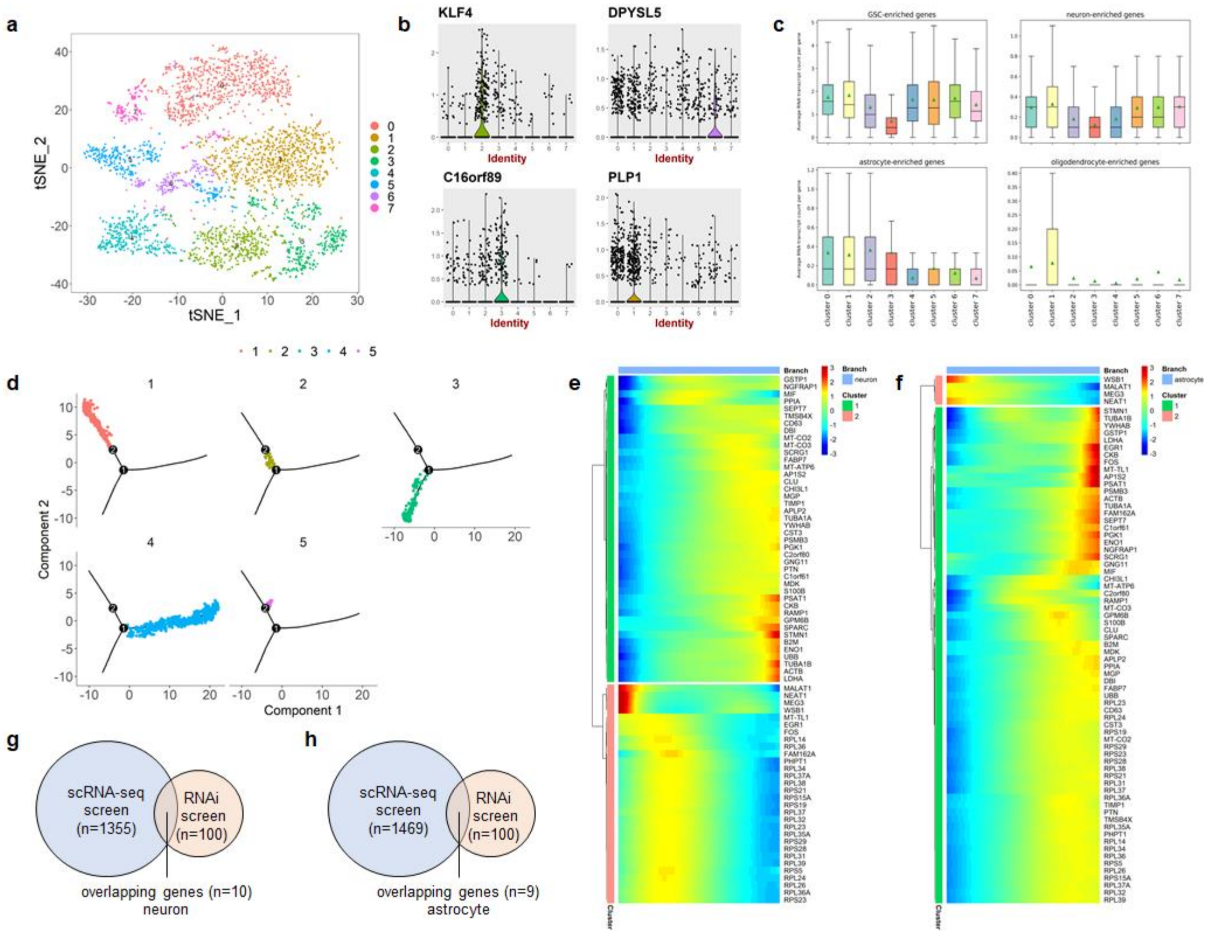

Extended Data Fig. 2. Analysis of GS5 scRNA-seq data. **a**, tSNE of scRNA-seq data from GS5 cells. Unsupervised clustering based on top 1,000 variable genes reveals cell-cell heterogeneity within the GS5 tumor population. **b**, Violin plot of individual gene markers used to identify cell types from clusters. *KLF4*[1], *DPYSL5*[2], *C16orf89*[2], and *PLP1*[2] were used to identify GSCs, neurons, astrocytes, and oligodendrocytes, respectively. **c**, Box-and-whisker plots of canonical gene marker sets used to identify cell types from clusters (the middle, bottom, and top lines correspond to the median, bottom, and top quartile, and whiskers to lower and upper extremes minus bottom quartile and top quartile, respectively). Units are the average number of mRNA transcripts for each gene set per cell. Canonical cell type markers include: *CD133*[3], *OLIG2*[4], *ITGA6*[5], *PDGFRA*[6], *DLL3*[7], *OLIG1*[7] used to define GSCs. *NEFL*[8], *RBFOX3*[9], *GRIA3*[4], *STMN2*[1], *COBL*[2], *LIN7C*[10], *DCX*[11], and *CD24*[12] used to define neurons, *GFAP*[13], *PAQR6*[2], *PPP1R3C*[2], *C16orf89*[2], *ALDOC*[14], and *AQP4*[2] used to define astrocytes, and *MAL*[2], *PLP1*[2], *MBP*[2], *MAG*[2], and *MOG*[14] used to define oligodendrocytes. Enriched gene score was calculated as the sum of the average number of mRNA transcripts per gene for each cell type. **d**, Cell states of single-cell trajectories. States 1, 3, and 4 were used for GSC to neuron, oligodendrocyte, and astrocyte differentiation, respectively. **e,f**, Venn diagram of significant genes that regulate (e) neurons and (f) astrocyte differentiation from single-cell trajectory analysis and Nestin expression from the RNAi screen. *ZNF117* does not overlap for either cell type. **g,h**, Heatmap of significant genes that regulate GSC to oligodendrocyte differentiation. *ZNF117* decreases in expression as GSCs differentiation into (g) neurons, and (h) astrocytes. Heatmap colors represent normalized gene expression across pseudotime. Genes are

hierarchically clustered according to their expression pattern. q-val < 1e-320.

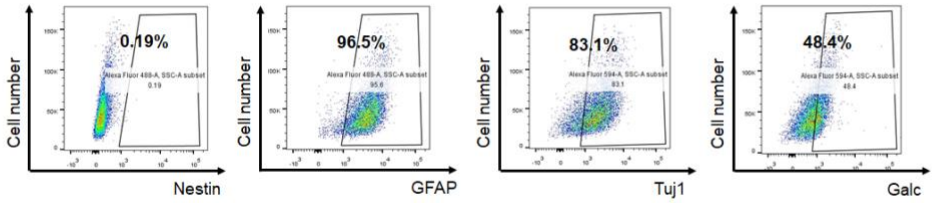

Extended Data Fig. 3. Flow cytometry analysis of FBS-differentiated GS5 cells. Cells were cultured in DMEM medium supplemented with 10% FBS. Two weeks later, the cells were collected, stained with the indicated antibodies, and subjected to flow cytometry analysis.

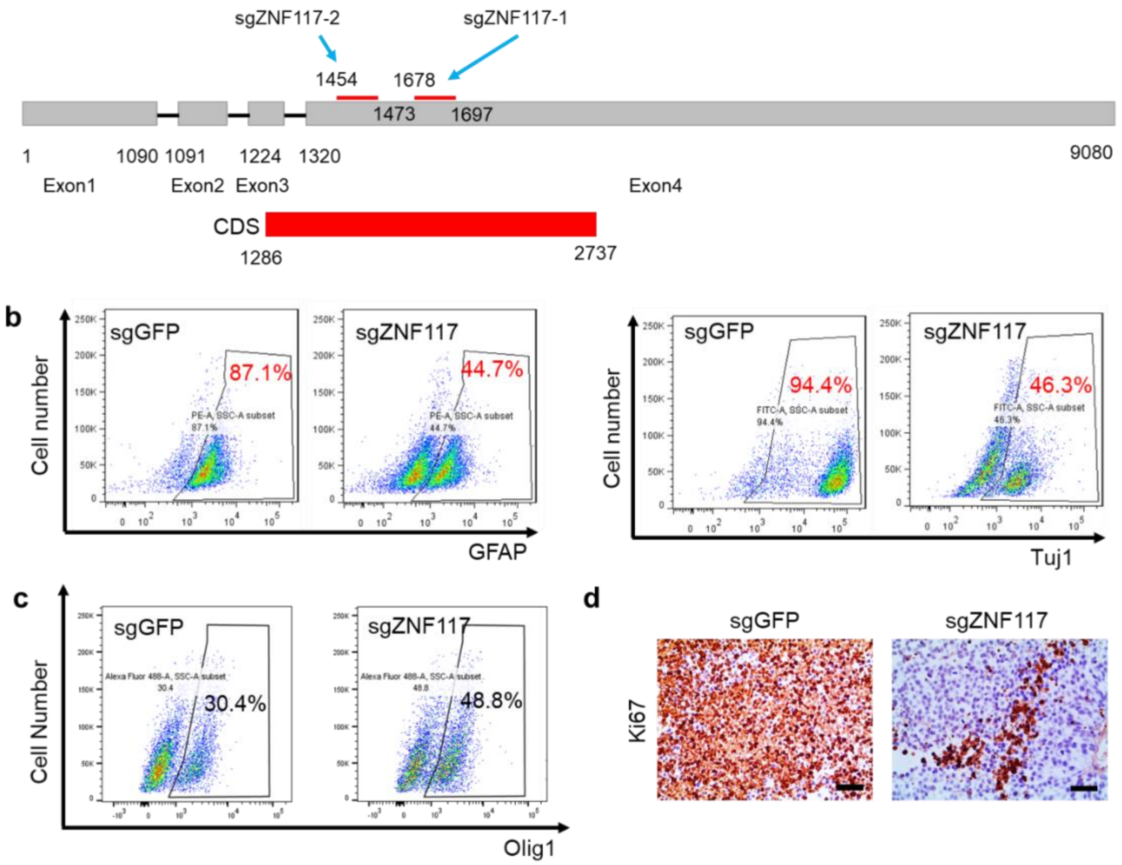

Extended Data Fig. 4. ***ZNF117* regulates GSC differentiation towards oligodendroglial lineage. a**, Schematic of sgRNA locations in *ZNF117* gene. **b,c**, Flow cytometry analyses of GFAP+ and Tuj1+ (b) and Olig1+ (c) cell populations in GS5 cells after treatment with Cas9 and the indicated sgRNAs. **d**, Ki67 staining of residual tumors isolated from mice received inoculation of the indicated cells. Images are representative of 3 independent experiments. Scale bar, 50 μm.

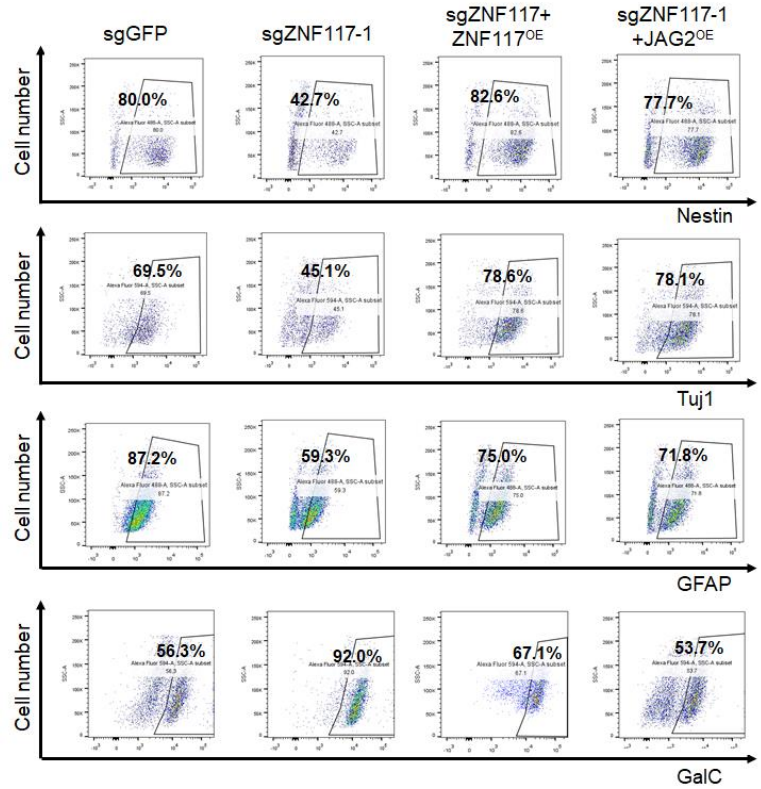

Extended Data Fig. 5. Flow cytometry analysis of genetically engineered GS5 cells. Cells treated with Cas9/sgGFP, Cas9/sgZNF117, Cas9/sgZNF117 following with *ZNF117* overexpression, and Cas9/sgZNF117 following with *JAG2* overexpression were stained with the indicated antibodies and subjected to flow cytometry analysis. *Jagged 2* (human, BC032053) cDNA and *ZNF117* (Human, BC034021) cDNA were purchased from Horizon Discovery and subcloned into vector pLenti CMV Blast (a gift from Eric Campeau & Paul Kaufman, Addgene plasmid# 17486) [15]. Overexpression was performed through lentiviral transduction.

a

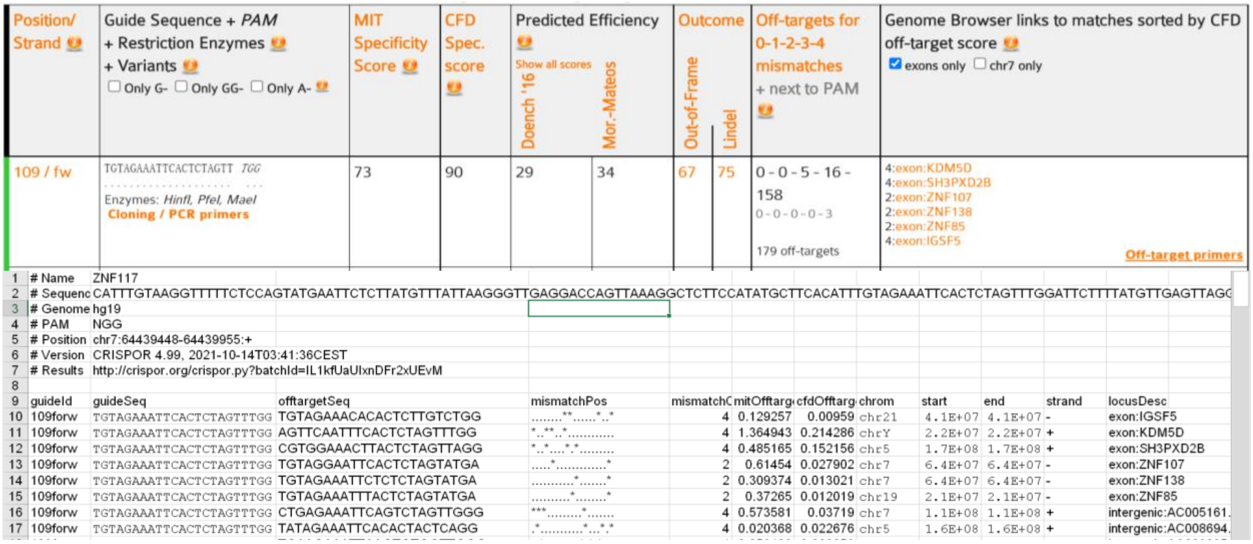
Position/ Strand
Guide Sequence + PAM + Restriction Enzymes + Variants
MIT Specificity Score
CFD Spec. score
Predicted Efficiency
Doench '16
Mor.-Mateos
Outcome
Out-of-Frame
Lindel
Off-targets for 0-1-2-3-4 mismatches + next to PAM
Genome Browser links to matches sorted by CFD off-target score
109 / fw
TGTAGAAATTCACTCTAGTT TGG
Enzymes: HinfI, PfeI, MaeI
Cloning / PCR primers
73
90
29
34
67
75
0 - 0 - 5 - 16 - 158
0 - 0 - 0 - 0 - 3
179 off-targets
4:exon:KDM5D
4:exon:SH3PXD2B
2:exon:ZNF107
2:exon:ZNF138
2:exon:ZNF85
4:exon:IGSF5
Off-target primers
Name ZNF117
Genome hg19
PAM NGG
Position chr7:64439448-64439955:+
Version CRISPOR 4.99, 2021-10-14T03:41:36CEST
Results http://crispor.org/crispor.py?batchId=IL1kfUaUlxnDFr2xUEvM
guideId guideSeq offtargetSeq mismatchPos mitOfftarget cfdOfftarget chrom start end strand locusDesc

b

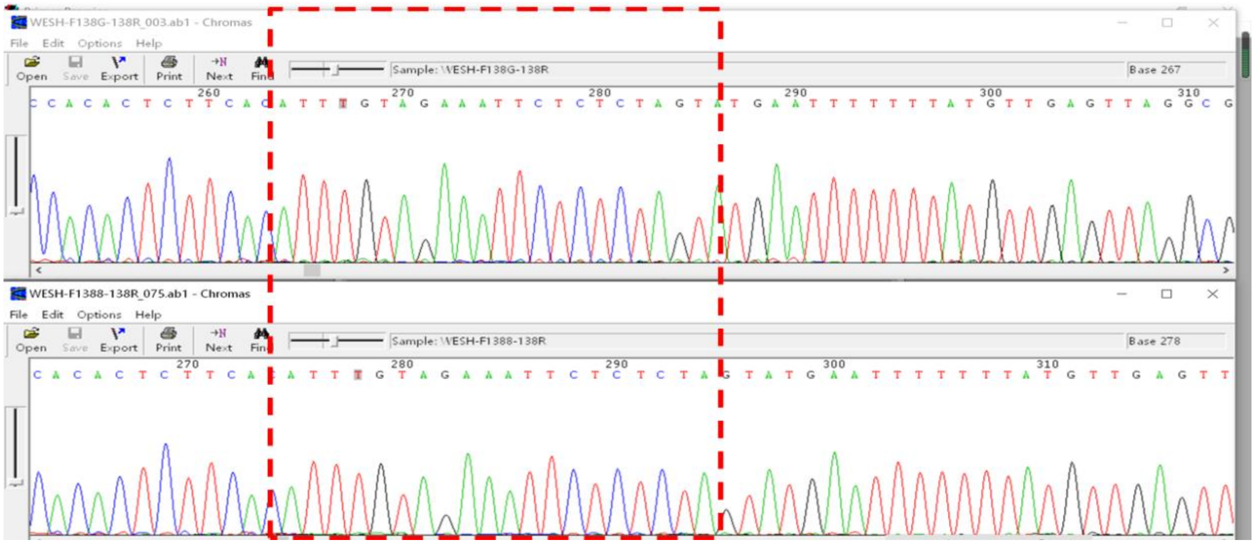
WESH-F138G-138R_003.ab1 - Chromas
Sample: WESH-F138G-138R
Base 267
WESH-F1388-138R_075.ab1 - Chromas
Sample: WESH-F1388-138R
Base 278

c

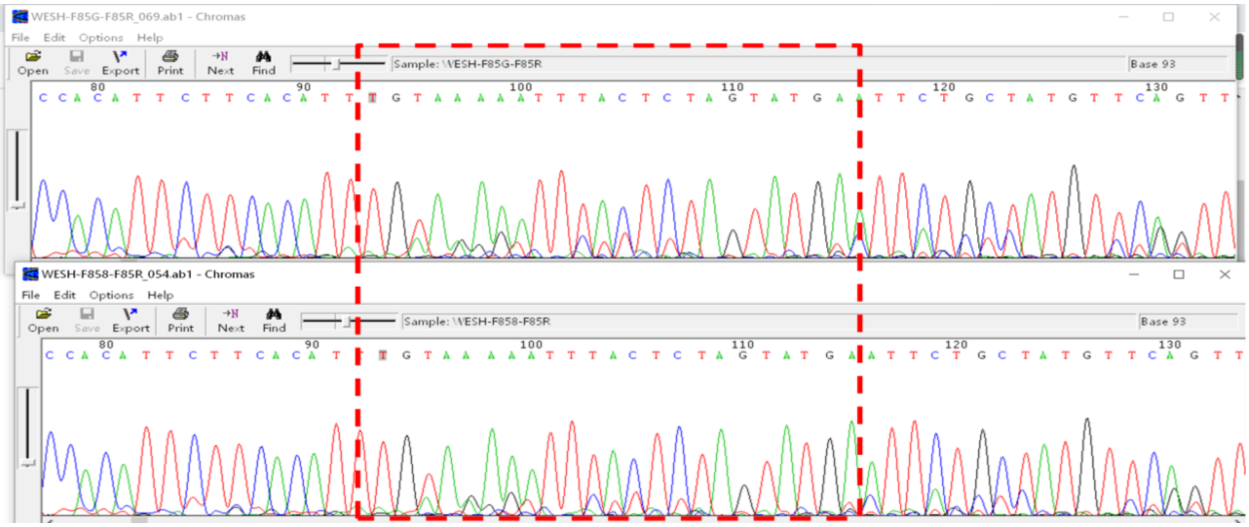
WESH-F85G-F85R_069.ab1 - Chromas
Sample: WESH-F85G-F85R
Base 93
WESH-F858-F85R_054.ab1 - Chromas
Sample: WESH-F858-F85R
Base 93

d

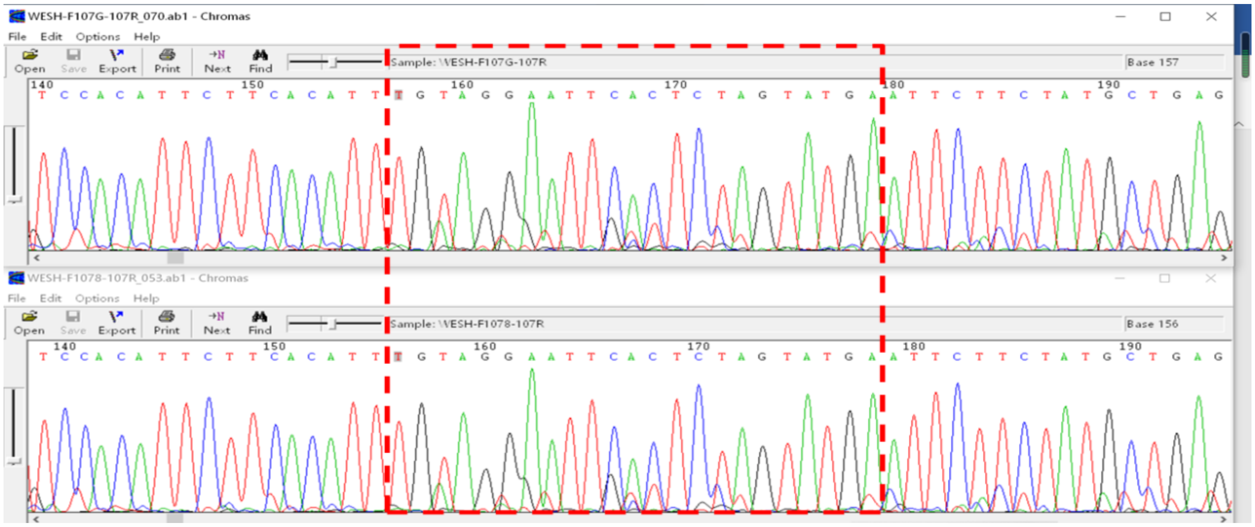
WESH-F107G-107R_070.ab1 - Chromas
Sample: \WESH-F107G-107R
Base 157
WESH-F1078-107R_053.ab1 - Chromas
Sample: \WESH-F1078-107R
Base 156

e

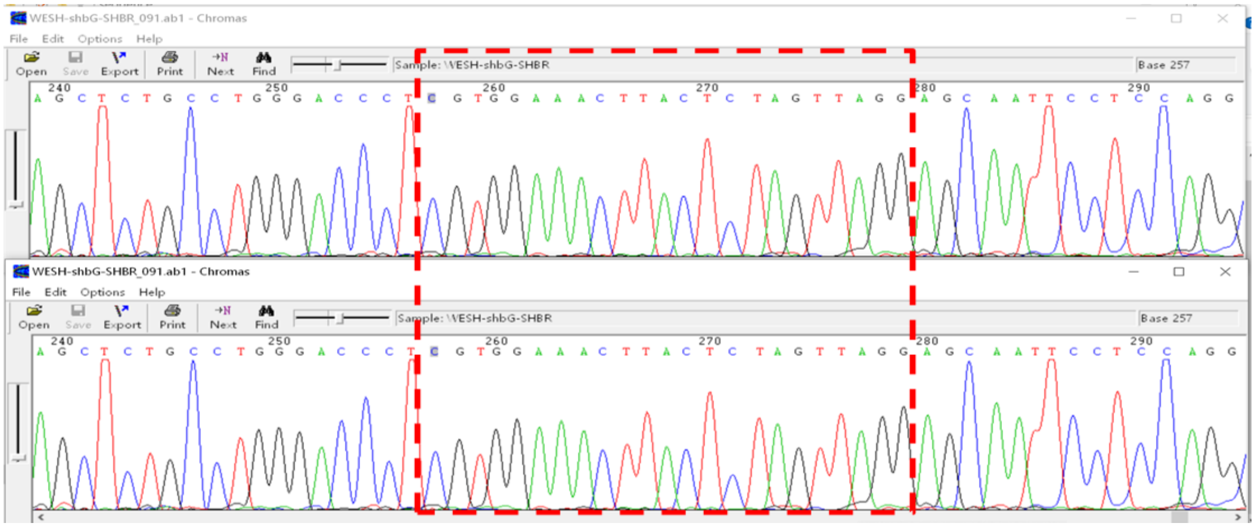
WESH-shbG-SHBR_091.ab1 - Chromas
Sample: \WESH-shbG-SHBR
Base 257
WESH-shbG-SHBR_091.ab1 - Chromas
Sample: \WESH-shbG-SHBR
Base 257

f

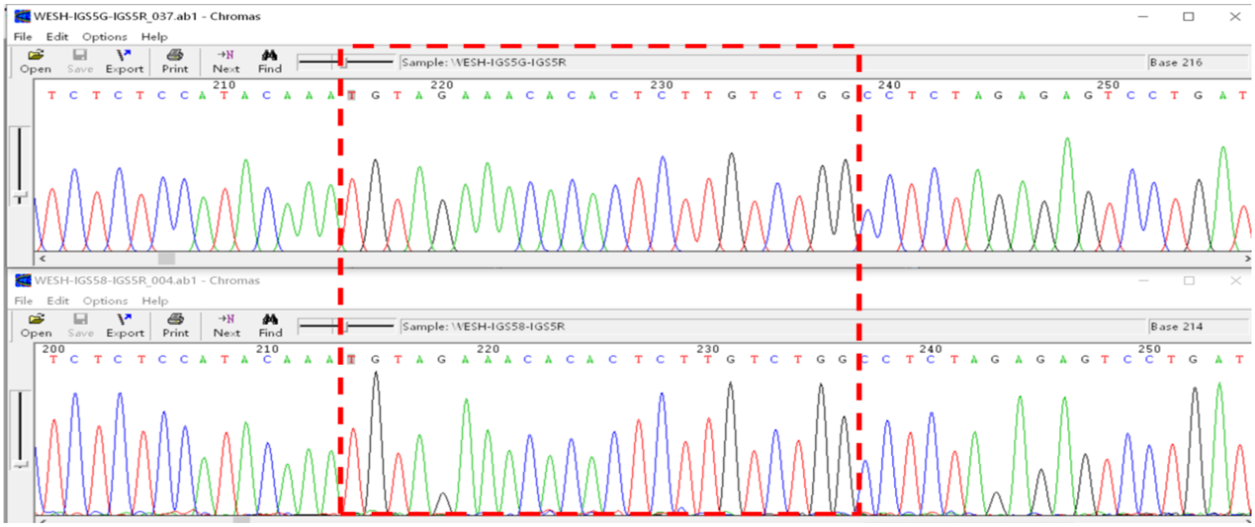
WESH-IGS5G-IGS5R_037.ab1 - Chromas
Sample: \WESH-IGS5G-IGS5R
Base 216
WESH-IGS58-IGS5R_004.ab1 - Chromas
Sample: \WESH-IGS58-IGS5R
Base 214

Extended Data Fig. 6. Characterization of potential off-target effects of *ZNF117* sgRNA-1. a, Bioinformatic analysis of candidate genes carrying mismatched sequences in exons. Analysis was carried out using CRISPOR (http://crispor.tefor.net/crispor.py) [16]. **b-f**, Sequencing analysis did not find significant activities in *ZNF138* (b), *ZNF85* (c), *ZNF107* (d), *SH3PXD2B* (e) and *IGSF5* (f). Upper and bottom panels illustrate the sequences in $GS5^{sgGFP}$ cells and $GS5^{sgZNF117\text{-}1}$ cells, respectively. Sequencing analysis did not find expression of *KDM5D* and thus was not included.

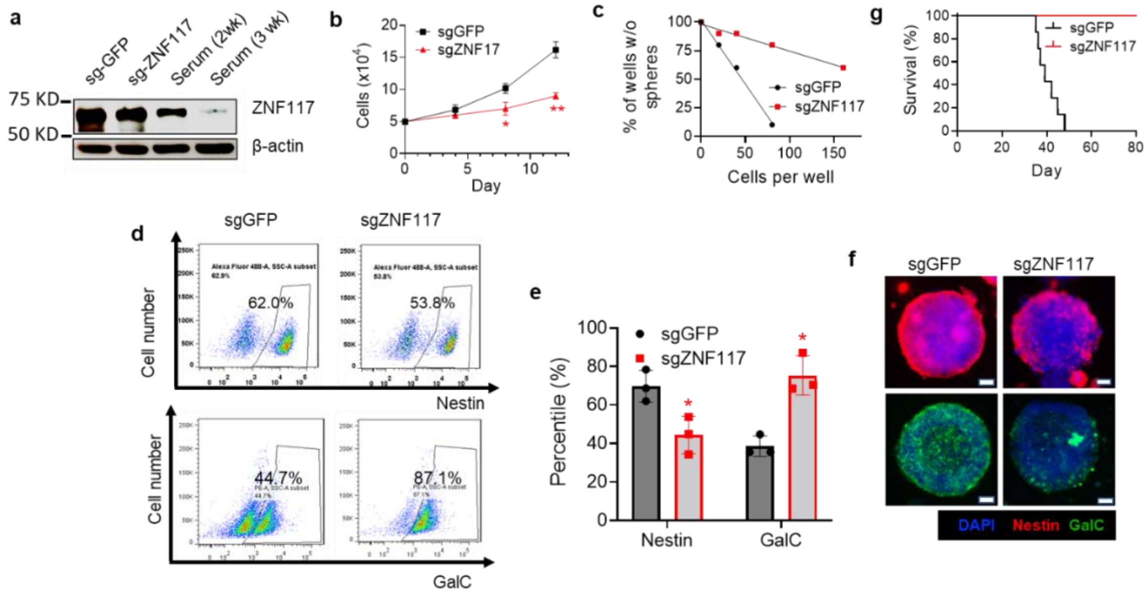

Extended Data Fig. 7. **Validation of the differentiation effect of *ZNF117* in PS24 cells. a**, WB analysis of the expression of *ZNF117* in PS24 cells cultured in serum-containing medium or treated with Cas9 and the indicated sgRNAs. **b,c**, Proliferation (n=3 biologically independent samples) (b) and stem cell frequency (c) of PS24 cells treated with Cas9 and the indicated sgRNAs **d-f**, Representative flow cytometry images (d) and corresponding quantification (n=3 biologically independent samples) (e), and immunostaining (f) of Nestin+ and GalC+ cell populations in PS24 cells after treatment with Cas9 and the indicated sgRNAs. Scale bar, 50 µm. **g**, Kaplan Meier survival analysis of the mice received inoculation of the indicated cells. All data are presented as mean ± SD. **P*-value < 0.05; ***P*-value < 0.01. Statistical differences were determined by two-tailed student's t test. Images are representative of 3 independent experiments.

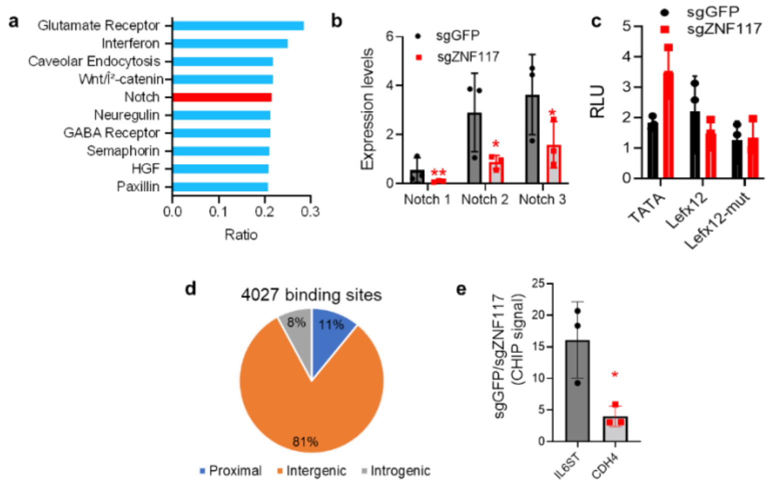

Extended Data Fig. 8. **Characterization of signaling regulated by *ZNF117*. a**, Analysis of pathways regulated by *ZNF117* based on whole-transcript expression analysis. **b**, Validation of the expression of Notch 1, 2, 3 in GS5 cells treated with Cas9 and sgGFP or sgZNF117 (n=3 biologically independent samples). **c**, Pathway reporter assay suggests that ZNF117 doesn't modulate Wnt activity (n=3 biologically independent samples). **d**, Distribution of the *ZNF117* binding regions identified by ChIP-Seq. **e**, ChIP-PCR analysis confirmed *ZNF117* binds with the indicated genes (n=3 biologically independent samples). All data are presented as mean ± SD. **P*-value < 0.05. Statistical differences were determined by two-tailed student's t test. Source data are provided as a Source Data file.

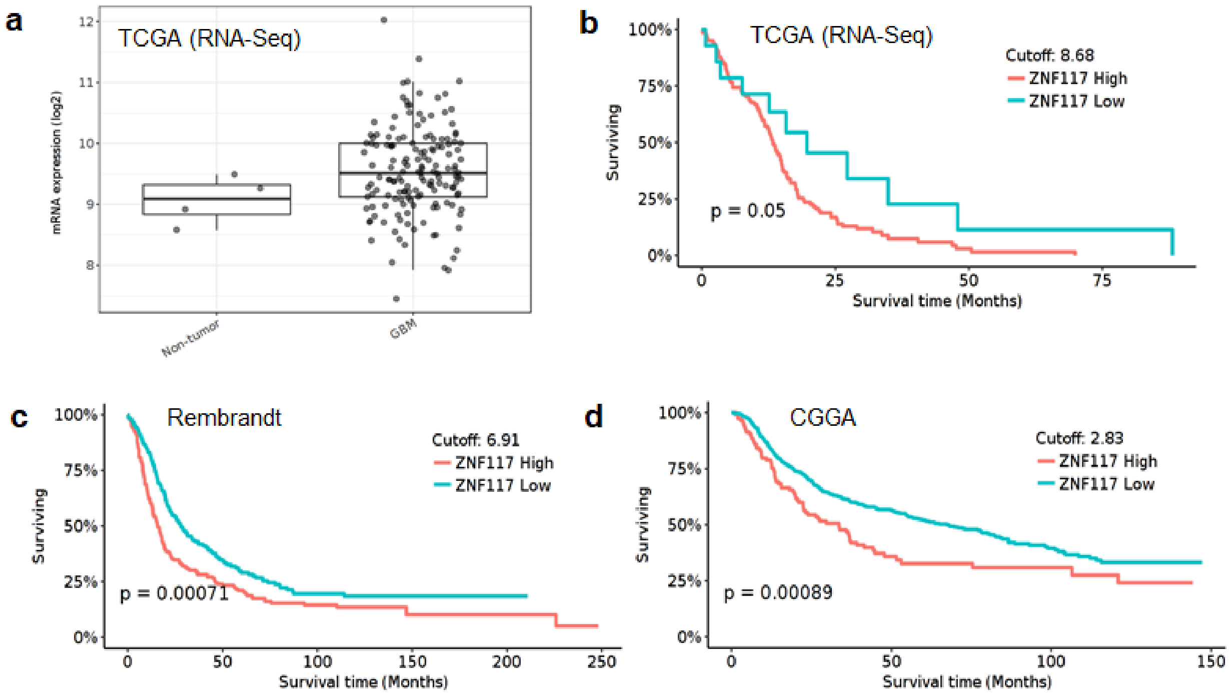

Extended Data Fig. 9. Database analysis of *ZNF117*. **a**, Analysis of TCGA RNA-seq database suggests that *ZNF117* expresses in a higher level in GBM than normal brain tissues (the middle, bottom, and top lines correspond to the median, bottom, and top quartile). **b-d**, Analyses of TCGA RNA-seq database (b), Rembrandt database (c) and CGGA (d) databased suggests that *ZNF117* is negatively correlated with patient survival. Maximally selected rank statistics was used to determine the optimal cutoff for Kaplan-Meier survival analysis as provided in the 'survminer' package in GlioVis (GlioVis data portal for visualization and analysis of brain tumor expression datasets, Neuro Oncol. 2017; 19(1):139-141). Specific parameters used for the analyses include: a&b: Dataset: adult “TCGA_GBM”, Platform: “RNA-seq”, Cutoff value: “optimal cutoff”; c, Dataset: adult “Rembrandt”, Cutoff value: “optimal cutoff”; d, Dataset: adult “CGGA”, Tumor type: “Primary”, Histology: “All”; Cutoff value: “optimal cutoff”.

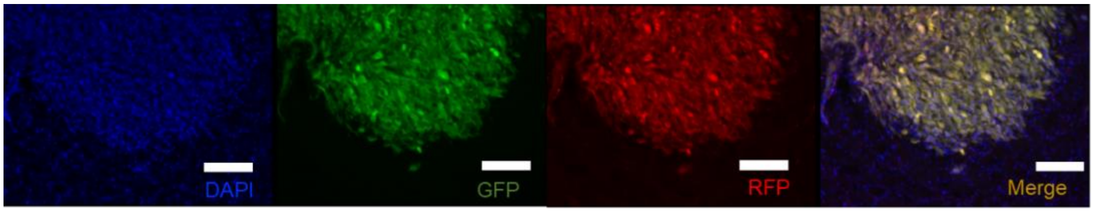

Extended Data Fig. 10. Imaging of tumor transfection by LHNPs. PS30 cells were engineered to expression GFP and inoculated into the brains of nude mice. Three weeks later, the mice were treated with LNHPs encapsulated with RFP-expression plasmid (pPRIME-CMV-dsRed, Addgene plasmid # 11658)[17]. Three days later, the mice were euthanized. The brains were isolated, sectioned, and imaged. Images are representative of 3 independent experiments. Scale bar, 50 μm.

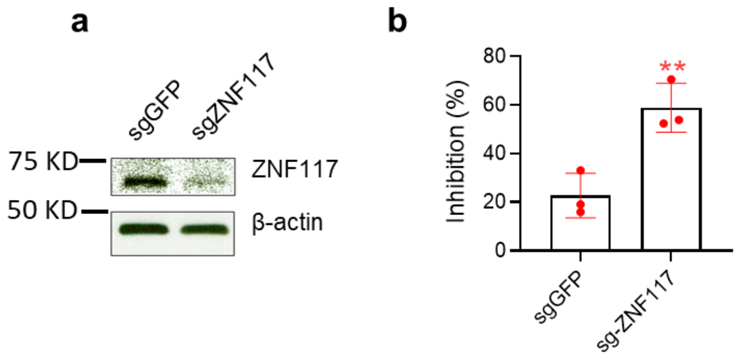

Extended Data Fig. 11. Characterization of *ZNF117* as a therapeutic target. a, WB analysis of *ZNF117* expression in GS5 cells treated with LHNPs loaded with Cas9 and sgGFP or sgZNF117. **b**, Inhibition of the proliferation of GS5 cells treated with Cas9 with the indicated sgRNA by TMZ (n=3 biologically independent samples). All data are presented as mean ± SD. ***P*-value < 0.01. Statistical differences were determined by two-tailed student's t test.

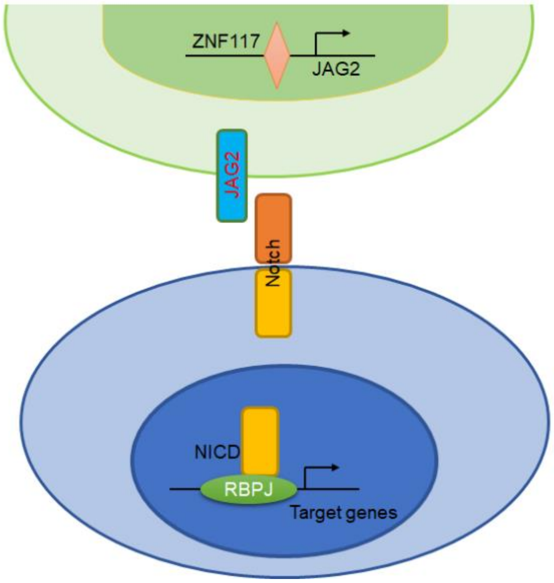

Extended Data Fig. 12. Schematic representation of *ZNF117*-mediated GSC differentiation through interaction with *JAG2*.

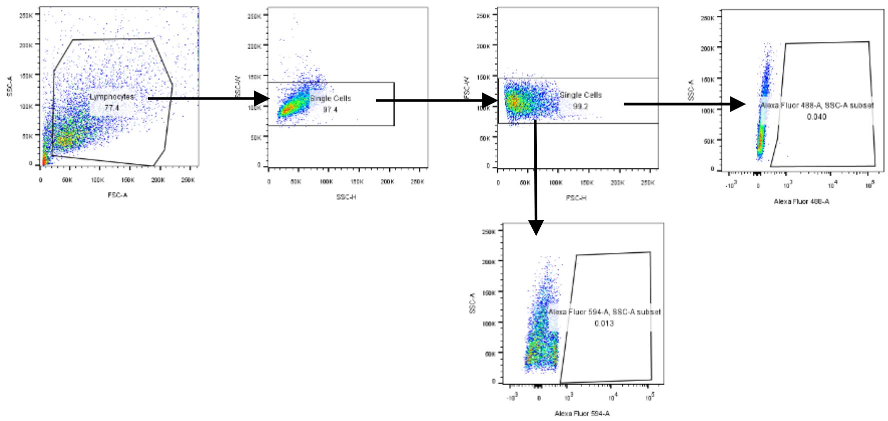

Extended Data Fig. 13. A representative flow cytometry gating strategy.

Table S1. Validation of top 100 genes.

siRNA ID	Gene Symbol	Plate	PlateID	WellRef	Cells Analysed	Nes+ (%)	Growth inhibition (%)
AB00477535	ACVRL1	1	PY00001012	A003	1193.00	17.59	3.07
AB00477535	ACVRL1	1	PY00001019	A003	483.00	6.51	15.08
AB00477535	ACVRL1	1	PY00000207	A003	525.00	53.13	-12.88
AB00477536	ACVRL1	1	PY00001012	A004	1263.00	24.54	-1.06
AB00477536	ACVRL1	1	PY00001019	A004	443.00	4.85	17.59
AB00477536	ACVRL1	1	PY00000207	A004	546.00	-5.62	-8.23
AB00477537	ACVRL1	1	PY00001012	A005	1299.00	-8.24	-3.18
AB00477537	ACVRL1	1	PY00001019	A005	497.00	-12.92	14.07
AB00477537	ACVRL1	1	PY00000207	A005	570.00	10.85	-26.51
AB00477538	ACVRL1	1	PY00001012	A006	1077.00	47.54	11.45
AB00477538	ACVRL1	1	PY00001019	A006	567.00	45.61	-6.78
AB00477538	ACVRL1	1	PY00000207	A006	638.00	29.60	-47.99
AB00477539	AD-003	1	PY00001012	A007	1171.00	-19.47	8.08
AB00477539	AD-003	1	PY00001019	A007	431.00	-12.11	37.19
AB00477539	AD-003	1	PY00000207	A007	353.00	-26.08	37.32
AB00477540	AD-003	1	PY00001012	A008	1260.00	23.75	0.57
AB00477540	AD-003	1	PY00001019	A008	493.00	33.91	14.32
AB00477540	AD-003	1	PY00000207	A008	572.00	27.01	-20.71
AB00477541	AD-003	1	PY00001012	A009	1144.00	31.63	11.74
AB00477541	AD-003	1	PY00001019	A009	440.00	9.36	27.89
AB00477541	AD-003	1	PY00000207	A009	557.00	23.91	-13.75
AB00477542	AD-003	1	PY00001012	A010	1402.00	34.70	-16.85
AB00477542	AD-003	1	PY00001019	A010	487.00	-12.29	11.81
AB00477542	AD-003	1	PY00000207	A010	498.00	-15.14	1.92
AB00477543	AKT1	1	PY00001012	A011	1179.00	50.03	4.33
AB00477543	AKT1	1	PY00001019	A011	603.00	35.24	-6.78
AB00477543	AKT1	1	PY00000207	A011	485.00	81.71	2.79
AB00477544	AKT1	1	PY00001012	A012	1110.00	17.53	13.95
AB00477544	AKT1	1	PY00001019	A012	458.00	-3.81	21.86
AB00477544	AKT1	1	PY00000207	A012	520.00	-10.31	-5.04
AB00477545	AKT1	1	PY00001012	A013	1224.00	33.06	5.67
AB00477545	AKT1	1	PY00001019	A013	407.00	-5.80	31.66
AB00477545	AKT1	1	PY00000207	A013	502.00	-10.84	3.37
AB00477546	AKT1	1	PY00001012	A014	1409.00	44.37	-16.56
AB00477546	AKT1	1	PY00001019	A014	502.00	73.06	8.29
AB00477546	AKT1	1	PY00000207	A014	387.00	85.61	3.66
AB00477547	ALDOA	1	PY00001012	A015	1175.00	34.12	1.92
AB00477547	ALDOA	1	PY00001019	A015	421.00	9.51	32.66
AB00477547	ALDOA	1	PY00000207	A015	593.00	46.18	-26.22
AB00477548	ALDOA	1	PY00001012	A016	1068.00	-0.09	18.19
AB00477548	ALDOA	1	PY00001019	A016	422.00	-4.12	29.15
AB00477548	ALDOA	1	PY00000207	A016	474.00	-2.86	11.79

AB00477549	ALDOA	1	PY00001012	A017	1124.00	0.30	10.20
AB00477549	ALDOA	1	PY00001019	A017	341.00	-24.44	55.78
AB00477549	ALDOA	1	PY00000207	A017	361.00	-29.59	42.26
AB00477550	ALDOA	1	PY00001012	A018	1477.00	59.67	-19.74
AB00477550	ALDOA	1	PY00001019	A018	524.00	22.92	9.55
AB00477550	ALDOA	1	PY00000207	A018	673.00	47.82	-47.12
AB00477551	ARRB1	1	PY00001012	A019	1299.00	26.11	-6.55
AB00477551	ARRB1	1	PY00001019	A019	486.00	14.22	20.35
AB00477551	ARRB1	1	PY00000207	A019	567.00	10.79	-16.36
AB00477552	ARRB1	1	PY00001012	A020	1503.00	38.86	-23.68
AB00477552	ARRB1	1	PY00001019	A020	421.00	11.67	23.62
AB00477552	ARRB1	1	PY00000207	A020	682.00	105.44	-45.96
AB00477553	ARRB1	1	PY00001012	A021	1183.00	47.00	3.07
AB00477553	ARRB1	1	PY00001019	A021	543.00	32.04	2.76
AB00477553	ARRB1	1	PY00000207	A021	414.00	89.40	14.11
AB00477554	ARRB1	1	PY00001012	A022	1503.00	58.70	-23.88
AB00477554	ARRB1	1	PY00001019	A022	481.00	-4.05	12.56
AB00477554	ARRB1	1	PY00000207	A022	347.00	5.95	36.16
AB00477555	ARRB2	1	PY00001012	B003	1080.00	-7.34	19.63
AB00477555	ARRB2	1	PY00001019	B003	474.00	-25.53	23.62
AB00477555	ARRB2	1	PY00000207	B003	593.00	-3.66	-26.22
AB00477556	ARRB2	1	PY00001012	B004	1204.00	15.16	0.19
AB00477556	ARRB2	1	PY00001019	B004	712.00	7.68	-35.93
AB00477556	ARRB2	1	PY00000207	B004	594.00	6.44	-23.61
AB00477557	ARRB2	1	PY00001012	B005	1416.00	19.89	-18.58
AB00477557	ARRB2	1	PY00001019	B005	721.00	13.71	-33.17
AB00477557	ARRB2	1	PY00000207	B005	478.00	34.17	7.15
AB00477558	ARRB2	1	PY00001012	B006	1182.00	8.60	4.42
AB00477558	ARRB2	1	PY00001019	B006	532.00	66.82	-0.25
AB00477558	ARRB2	1	PY00000207	B006	452.00	52.49	11.21
AB00477559	AURKB	1	PY00001012	B007	1308.00	22.92	-12.33
AB00477559	AURKB	1	PY00001019	B007	619.00	-21.42	-8.29
AB00477559	AURKB	1	PY00000207	B007	600.00	28.11	-22.74
AB00477560	AURKB	1	PY00001012	B008	1148.00	3.35	3.94
AB00477560	AURKB	1	PY00001019	B008	624.00	15.49	-1.26
AB00477560	AURKB	1	PY00000207	B008	430.00	37.99	15.56
AB00477561	AURKB	1	PY00001012	B009	1120.00	4.53	10.29
AB00477561	AURKB	1	PY00001019	B009	648.00	12.52	-9.80
AB00477561	AURKB	1	PY00000207	B009	470.00	-19.98	12.66
AB00477562	AURKB	1	PY00001012	B010	1274.00	4.25	-4.24
AB00477562	AURKB	1	PY00001019	B010	641.00	12.26	-11.81
AB00477562	AURKB	1	PY00000207	B010	461.00	-17.20	7.15
AB00477563	BLOC1S1	1	PY00001012	B011	1277.00	2.73	-5.20
AB00477563	BLOC1S1	1	PY00001019	B011	573.00	-22.47	1.76
AB00477563	BLOC1S1	1	PY00000207	B011	458.00	26.88	4.24
AB00477564	BLOC1S1	1	PY00001012	B012	1225.00	8.91	-2.32

AB00477564	BLOC1S1	1	PY00001019	B012	638.00	8.51	-9.30
AB00477564	BLOC1S1	1	PY00000207	B012	526.00	-5.05	-10.26
AB00477565	BLOC1S1	1	PY00001012	B013	1305.00	33.79	-9.15
AB00477565	BLOC1S1	1	PY00001019	B013	671.00	53.54	-22.11
AB00477565	BLOC1S1	1	PY00000207	B013	522.00	59.16	-5.62
AB00477566	BLOC1S1	1	PY00001012	B014	735.00	-8.09	43.98
AB00477566	BLOC1S1	1	PY00001019	B014	589.00	56.81	-3.52
AB00477566	BLOC1S1	1	PY00000207	B014	471.00	-0.83	3.37
AB00477567	BMP4	1	PY00001012	B015	1351.00	14.73	-10.69
AB00477567	BMP4	1	PY00001019	B015	541.00	6.91	11.31
AB00477567	BMP4	1	PY00000207	B015	450.00	4.58	12.08
AB00477568	BMP4	1	PY00001012	B016	1178.00	-2.77	6.73
AB00477568	BMP4	1	PY00001019	B016	589.00	18.20	-3.52
AB00477568	BMP4	1	PY00000207	B016	396.00	-38.53	23.69
AB00477569	BMP4	1	PY00001012	B017	1348.00	25.49	-13.10
AB00477569	BMP4	1	PY00001019	B017	682.00	53.31	-26.13
AB00477569	BMP4	1	PY00000207	B017	565.00	7.24	-19.26
AB00477570	BMP4	1	PY00001012	B018	1331.00	34.94	-10.11
AB00477570	BMP4	1	PY00001019	B018	640.00	21.36	-17.34
AB00477570	BMP4	1	PY00000207	B018	548.00	-27.30	-18.10
AB00477571	C1ORF40	1	PY00001012	B019	1284.00	21.42	-5.68
AB00477571	C1ORF40	1	PY00001019	B019	728.00	64.59	-39.20
AB00477571	C1ORF40	1	PY00000207	B019	514.00	11.92	-9.10
AB00477572	C1ORF40	1	PY00001012	B020	1012.00	-2.78	20.88
AB00477572	C1ORF40	1	PY00001019	B020	555.00	34.67	2.01
AB00477572	C1ORF40	1	PY00000207	B020	527.00	61.64	-11.72
AB00477573	C1ORF40	1	PY00001012	B021	1041.00	-21.03	19.63
AB00477573	C1ORF40	1	PY00001019	B021	608.00	14.06	-12.81
AB00477573	C1ORF40	1	PY00000207	B021	563.00	77.07	-21.29
AB00477574	C1ORF40	1	PY00001012	B022	1106.00	11.02	10.20
AB00477574	C1ORF40	1	PY00001019	B022	619.00	8.63	-10.30
AB00477574	C1ORF40	1	PY00000207	B022	454.00	27.30	4.53
AB00477575	C20ORF32	1	PY00001012	C003	1154.00	78.46	4.52
AB00477575	C20ORF32	1	PY00001019	C003	551.00	93.98	-7.29
AB00477575	C20ORF32	1	PY00000207	C003	548.00	76.80	-19.26
AB00477576	C20ORF32	1	PY00001012	C004	1185.00	14.34	4.13
AB00477576	C20ORF32	1	PY00001019	C004	185.00	57.05	64.57
AB00477576	C20ORF32	1	PY00000207	C004	476.00	5.56	5.11
AB00477577	C20ORF32	1	PY00001012	C005	1353.00	1.34	-11.46
AB00477577	C20ORF32	1	PY00001019	C005	521.00	24.46	7.04
AB00477577	C20ORF32	1	PY00000207	C005	655.00	43.23	-45.67
AB00477578	C20ORF32	1	PY00001012	C006	1668.00	41.96	-45.63
AB00477578	C20ORF32	1	PY00001019	C006	694.00	59.67	-29.15
AB00477578	C20ORF32	1	PY00000207	C006	514.00	-10.11	-6.20
AB00477579	C2ORF28	1	PY00001012	C007	1069.00	-4.23	14.91
AB00477579	C2ORF28	1	PY00001019	C007	449.00	-2.97	26.88

AB00477579	C2ORF28	1	PY00000207	C007	520.00	-7.19	-15.20
AB00477580	C2ORF28	1	PY00001012	C008	1434.00	31.85	-23.49
AB00477580	C2ORF28	1	PY00001019	C008	602.00	10.71	-9.30
AB00477580	C2ORF28	1	PY00000207	C008	414.00	-14.83	21.65
AB00477581	C2ORF28	1	PY00001012	C009	870.00	-10.61	38.30
AB00477581	C2ORF28	1	PY00001019	C009	376.00	29.89	42.21
AB00477581	C2ORF28	1	PY00000207	C009	437.00	36.94	8.89
AB00477582	C2ORF28	1	PY00001012	C010	1112.00	3.89	11.26
AB00477582	C2ORF28	1	PY00001019	C010	416.00	-22.24	34.17
AB00477582	C2ORF28	1	PY00000207	C010	545.00	-1.14	-12.01
AB00477583	CARD9	1	PY00001012	C011	1107.00	5.24	12.80
AB00477583	CARD9	1	PY00001019	C011	495.00	-12.49	17.84
AB00477583	CARD9	1	PY00000207	C011	556.00	35.03	-20.71
AB00477584	CARD9	1	PY00001012	C012	1238.00	-12.46	-2.41
AB00477584	CARD9	1	PY00001019	C012	455.00	-2.74	22.36
AB00477584	CARD9	1	PY00000207	C012	567.00	14.99	-20.71
AB00477585	CARD9	1	PY00001012	C013	963.00	-40.25	35.22
AB00477585	CARD9	1	PY00001019	C013	429.00	-10.51	32.66
AB00477585	CARD9	1	PY00000207	C013	437.00	8.90	11.79
AB00477586	CARD9	1	PY00001012	C014	1600.00	27.70	-36.77
AB00477586	CARD9	1	PY00001019	C014	579.00	-6.11	-2.26
AB00477586	CARD9	1	PY00000207	C014	553.00	47.82	-21.87
AB00477587	CASP8AP2	1	PY00001012	C015	1284.00	21.01	-6.45
AB00477587	CASP8AP2	1	PY00001019	C015	539.00	7.02	1.26
AB00477587	CASP8AP2	1	PY00000207	C015	542.00	-1.05	-13.17
AB00477588	CASP8AP2	1	PY00001012	C016	1365.00	26.98	-13.19
AB00477588	CASP8AP2	1	PY00001019	C016	533.00	51.74	0.75
AB00477588	CASP8AP2	1	PY00000207	C016	505.00	-33.47	1.92
AB00477589	CASP8AP2	1	PY00001012	C017	1319.00	13.97	-10.50
AB00477589	CASP8AP2	1	PY00001019	C017	551.00	-15.03	0.25
AB00477589	CASP8AP2	1	PY00000207	C017	516.00	-22.50	-3.88
AB00477590	CASP8AP2	1	PY00001012	C018	1658.00	49.91	-40.62
AB00477590	CASP8AP2	1	PY00001019	C018	475.00	-6.34	15.33
AB00477590	CASP8AP2	1	PY00000207	C018	571.00	52.95	-25.64
AB00477591	CCNB1	1	PY00001012	C019	867.00	-17.93	34.55
AB00477591	CCNB1	1	PY00001019	C019	356.00	-43.61	46.73
AB00477591	CCNB1	1	PY00000207	C019	293.00	-51.05	56.18
AB00477592	CCNB1	1	PY00001012	C020	979.00	-19.78	29.54
AB00477592	CCNB1	1	PY00001019	C020	301.00	-37.12	61.56
AB00477592	CCNB1	1	PY00000207	C020	367.00	-19.79	35.58
AB00477593	CCNB1	1	PY00001012	C021	844.00	-32.77	39.36
AB00477593	CCNB1	1	PY00001019	C021	279.00	-20.83	61.56
AB00477593	CCNB1	1	PY00000207	C021	307.00	-39.11	50.96
AB00477594	CCNB1	1	PY00001012	C022	1474.00	25.85	-25.03
AB00477594	CCNB1	1	PY00001019	C022	524.00	-10.60	7.29
AB00477594	CCNB1	1	PY00000207	C022	638.00	-1.94	-37.54

AB00477595	CCNB1IP1	1	PY00001012	D003	1428.00	20.60	-22.43
AB00477595	CCNB1IP1	1	PY00001019	D003	725.00	41.19	-33.17
AB00477595	CCNB1IP1	1	PY00000207	D003	602.00	20.08	-27.09
AB00477596	CCNB1IP1	1	PY00001012	D004	1415.00	29.71	-20.51
AB00477596	CCNB1IP1	1	PY00001019	D004	722.00	4.88	-33.42
AB00477596	CCNB1IP1	1	PY00000207	D004	540.00	4.52	-15.49
AB00477597	CCNB1IP1	1	PY00001012	D005	1227.00	-9.95	0.38
AB00477597	CCNB1IP1	1	PY00001019	D005	576.00	-21.67	-6.03
AB00477597	CCNB1IP1	1	PY00000207	D005	580.00	-45.66	-19.55
AB00477598	CCNB1IP1	1	PY00001012	D006	1440.00	12.61	-21.28
AB00477598	CCNB1IP1	1	PY00001019	D006	612.00	-11.10	-4.27
AB00477598	CCNB1IP1	1	PY00000207	D006	423.00	-18.58	13.24
AB00477599	CNTNAP5	1	PY00001012	D007	1306.00	33.89	-10.50
AB00477599	CNTNAP5	1	PY00001019	D007	618.00	12.58	-8.04
AB00477599	CNTNAP5	1	PY00000207	D007	562.00	21.23	-29.13
AB00477600	CNTNAP5	1	PY00001012	D008	1172.00	12.61	3.46
AB00477600	CNTNAP5	1	PY00001019	D008	674.00	83.18	-14.07
AB00477600	CNTNAP5	1	PY00000207	D008	452.00	42.41	6.27
AB00477601	CNTNAP5	1	PY00001012	D009	1325.00	-8.07	-7.42
AB00477601	CNTNAP5	1	PY00001019	D009	648.00	11.20	-20.60
AB00477601	CNTNAP5	1	PY00000207	D009	526.00	-15.59	-12.30
AB00477602	CNTNAP5	1	PY00001012	D010	1388.00	13.22	-18.10
AB00477602	CNTNAP5	1	PY00001019	D010	628.00	51.97	-15.58
AB00477602	CNTNAP5	1	PY00000207	D010	566.00	59.87	-26.22
AB00477603	COMMD1	1	PY00001012	D011	1027.00	-3.40	17.22
AB00477603	COMMD1	1	PY00001019	D011	484.00	-41.50	23.12
AB00477603	COMMD1	1	PY00000207	D011	205.00	49.47	58.22
AB00477604	COMMD1	1	PY00001012	D012	1000.00	-3.82	19.05
AB00477604	COMMD1	1	PY00001019	D012	645.00	34.79	-12.56
AB00477604	COMMD1	1	PY00000207	D012	422.00	-20.44	15.27
AB00477605	COMMD1	1	PY00001012	D013	1222.00	1.11	2.02
AB00477605	COMMD1	1	PY00001019	D013	702.00	36.68	-36.43
AB00477605	COMMD1	1	PY00000207	D013	543.00	44.11	-18.10
AB00477606	COMMD1	1	PY00001012	D014	1205.00	-5.38	1.53
AB00477606	COMMD1	1	PY00001019	D014	680.00	31.93	-31.16
AB00477606	COMMD1	1	PY00000207	D014	357.00	82.00	14.40
AB00477607	COMMD3	1	PY00001012	D015	1144.00	-0.32	8.27
AB00477607	COMMD3	1	PY00001019	D015	461.00	-15.43	26.13
AB00477607	COMMD3	1	PY00000207	D015	533.00	38.11	-16.36
AB00477608	COMMD3	1	PY00001012	D016	1148.00	-8.57	6.15
AB00477608	COMMD3	1	PY00001019	D016	552.00	18.06	5.28
AB00477608	COMMD3	1	PY00000207	D016	415.00	8.94	16.43
AB00477609	COMMD3	1	PY00001012	D017	1297.00	12.88	-8.09
AB00477609	COMMD3	1	PY00001019	D017	711.00	36.70	-32.91
AB00477609	COMMD3	1	PY00000207	D017	472.00	5.64	2.21
AB00477610	COMMD3	1	PY00001012	D018	1001.00	-33.47	20.50

AB00477610	COMMD3	1	PY00001019	D018	511.00	1.31	14.32
AB00477610	COMMD3	1	PY00000207	D018	445.00	-21.37	12.08
AB00477611	CRYAA	1	PY00001012	D019	1530.00	34.31	-30.71
AB00477611	CRYAA	1	PY00001019	D019	675.00	29.23	-26.63
AB00477611	CRYAA	1	PY00000207	D019	511.00	-8.43	-4.46
AB00477612	CRYAA	1	PY00001012	D020	1196.00	2.31	2.02
AB00477612	CRYAA	1	PY00001019	D020	676.00	52.96	-29.15
AB00477612	CRYAA	1	PY00000207	D020	423.00	1.11	11.21
AB00477613	CRYAA	1	PY00001012	D021	1089.00	14.28	17.61
AB00477613	CRYAA	1	PY00001019	D021	527.00	20.23	10.55
AB00477613	CRYAA	1	PY00000207	D021	446.00	29.53	8.02
AB00477614	CRYAA	1	PY00001012	D022	1112.00	2.12	10.29
AB00477614	CRYAA	1	PY00001019	D022	560.00	55.70	-5.53
AB00477614	CRYAA	1	PY00000207	D022	539.00	44.34	-19.26
AB00477615	CSNK1A1	1	PY00001012	E003	1364.00	43.55	-14.25
AB00477615	CSNK1A1	1	PY00001019	E003	488.00	20.55	8.29
AB00477615	CSNK1A1	1	PY00000207	E003	544.00	-20.16	-14.91
AB00477616	CSNK1A1	1	PY00001012	E004	1188.00	-10.51	3.65
AB00477616	CSNK1A1	1	PY00001019	E004	484.00	-2.98	14.57
AB00477616	CSNK1A1	1	PY00000207	E004	533.00	-8.99	-7.07
AB00477617	CSNK1A1	1	PY00001012	E005	1387.00	21.27	-16.56
AB00477617	CSNK1A1	1	PY00001019	E005	384.00	-19.78	30.90
AB00477617	CSNK1A1	1	PY00000207	E005	450.00	-7.59	9.76
AB00477618	CSNK1A1	1	PY00001012	E006	1149.00	-13.44	10.29
AB00477618	CSNK1A1	1	PY00001019	E006	586.00	-44.00	0.25
AB00477618	CSNK1A1	1	PY00000207	E006	505.00	-10.41	-4.46
AB00477619	CSPG3	1	PY00001012	E007	1316.00	-1.11	-4.72
AB00477619	CSPG3	1	PY00001019	E007	493.00	54.15	10.55
AB00477619	CSPG3	1	PY00000207	E007	297.00	48.12	23.10
AB00477620	CSPG3	1	PY00001012	E008	1242.00	8.46	-1.55
AB00477620	CSPG3	1	PY00001019	E008	582.00	-5.13	-1.51
AB00477620	CSPG3	1	PY00000207	E008	617.00	-9.50	-31.45
AB00477621	CSPG3	1	PY00001012	E009	1393.00	37.63	-16.75
AB00477621	CSPG3	1	PY00001019	E009	509.00	-1.12	6.78
AB00477621	CSPG3	1	PY00000207	E009	514.00	-9.47	-4.17
AB00477622	CSPG3	1	PY00001012	E010	1337.00	16.03	-9.82
AB00477622	CSPG3	1	PY00001019	E010	614.00	41.14	-16.83
AB00477622	CSPG3	1	PY00000207	E010	587.00	24.97	-27.09
AB00477623	CTNNB1	1	PY00001012	E011	1024.00	-21.07	19.05
AB00477623	CTNNB1	1	PY00001019	E011	526.00	10.25	2.76
AB00477623	CTNNB1	1	PY00000207	E011	522.00	-20.12	-6.49
AB00477624	CTNNB1	1	PY00001012	E012	1295.00	16.95	-9.53
AB00477624	CTNNB1	1	PY00001019	E012	538.00	-1.87	-0.25
AB00477624	CTNNB1	1	PY00000207	E012	577.00	-43.88	-25.64
AB00477625	CTNNB1	1	PY00001012	E013	1539.00	-6.58	-25.13
AB00477625	CTNNB1	1	PY00001019	E013	599.00	3.99	-12.56

AB00477625	CTNNB1	1	PY00000207	E013	559.00	-28.56	-21.58
AB00477626	CTNNB1	1	PY00001012	E014	1092.00	39.15	7.31
AB00477626	CTNNB1	1	PY00001019	E014	586.00	23.69	-10.55
AB00477626	CTNNB1	1	PY00000207	E014	578.00	63.85	-26.51
AB00477627	DDB1	1	PY00001012	E015	1422.00	13.09	-18.58
AB00477627	DDB1	1	PY00001019	E015	612.00	19.15	-19.10
AB00477627	DDB1	1	PY00000207	E015	542.00	3.82	-12.59
AB00477628	DDB1	1	PY00001012	E016	1491.00	22.11	-23.78
AB00477628	DDB1	1	PY00001019	E016	486.00	22.39	18.09
AB00477628	DDB1	1	PY00000207	E016	496.00	46.24	-2.43
AB00477629	DDB1	1	PY00001012	E017	1103.00	-8.96	12.41
AB00477629	DDB1	1	PY00001019	E017	489.00	-8.60	16.08
AB00477629	DDB1	1	PY00000207	E017	473.00	3.98	-0.40
AB00477630	DDB1	1	PY00001012	E018	1297.00	39.65	-9.63
AB00477630	DDB1	1	PY00001019	E018	456.00	5.14	24.62
AB00477630	DDB1	1	PY00000207	E018	428.00	10.94	14.40
AB00477631	DISP2	1	PY00001012	E019	1317.00	-0.76	-8.09
AB00477631	DISP2	1	PY00001019	E019	538.00	-16.69	3.27
AB00477631	DISP2	1	PY00000207	E019	523.00	-34.60	-14.91
AB00477632	DISP2	1	PY00001012	E020	1587.00	34.71	-31.00
AB00477632	DISP2	1	PY00001019	E020	563.00	31.95	-8.29
AB00477632	DISP2	1	PY00000207	E020	623.00	20.89	-32.61
AB00477633	DISP2	1	PY00001012	E021	1319.00	13.39	-7.61
AB00477633	DISP2	1	PY00001019	E021	510.00	-15.27	7.29
AB00477633	DISP2	1	PY00000207	E021	488.00	30.69	0.47
AB00477634	DISP2	1	PY00001012	E022	1193.00	6.11	2.59
AB00477634	DISP2	1	PY00001019	E022	452.00	23.53	16.08
AB00477634	DISP2	1	PY00000207	E022	462.00	61.01	9.18
AB00477635	DKFZP564O243	1	PY00001012	F003	1218.00	11.55	0.09
AB00477635	DKFZP564O243	1	PY00001019	F003	599.00	8.00	-8.79
AB00477635	DKFZP564O243	1	PY00000207	F003	522.00	-5.26	-8.52
AB00477636	DKFZP564O243	1	PY00001012	F004	1117.00	-23.70	8.75
AB00477636	DKFZP564O243	1	PY00001019	F004	665.00	41.71	-24.62
AB00477636	DKFZP564O243	1	PY00000207	F004	391.00	-34.51	13.24
AB00477637	DKFZP564O243	1	PY00001012	F005	1267.00	21.82	-4.43
AB00477637	DKFZP564O243	1	PY00001019	F005	678.00	25.60	-27.39
AB00477637	DKFZP564O243	1	PY00000207	F005	506.00	-34.01	-14.04
AB00477638	DKFZP564O243	1	PY00001012	F006	1280.00	2.21	-7.03
AB00477638	DKFZP564O243	1	PY00001019	F006	594.00	12.55	-5.28
AB00477638	DKFZP564O243	1	PY00000207	F006	511.00	0.09	-7.65
AB00477639	DPP4	1	PY00001012	F007	1398.00	55.15	-19.83
AB00477639	DPP4	1	PY00001019	F007	691.00	19.93	-27.39
AB00477639	DPP4	1	PY00000207	F007	537.00	17.99	-12.01
AB00477640	DPP4	1	PY00001012	F008	1322.00	14.81	-12.42
AB00477640	DPP4	1	PY00001019	F008	675.00	37.93	-22.36
AB00477640	DPP4	1	PY00000207	F008	510.00	16.98	-9.10

AB00477641	DPP4	1	PY00001012	F009	1196.00	28.26	-1.26
AB00477641	DPP4	1	PY00001019	F009	600.00	14.55	-4.52
AB00477641	DPP4	1	PY00000207	F009	553.00	17.14	-14.91
AB00477642	DPP4	1	PY00001012	F010	1128.00	5.29	9.23
AB00477642	DPP4	1	PY00001019	F010	644.00	6.23	-8.79
AB00477642	DPP4	1	PY00000207	F010	417.00	-26.78	13.82
AB00477643	DUB3	1	PY00001012	F011	1656.00	76.29	-42.84
AB00477643	DUB3	1	PY00001019	F011	686.00	56.93	-23.37
AB00477643	DUB3	1	PY00000207	F011	607.00	11.97	-34.35
AB00477644	DUB3	1	PY00001012	F012	1050.00	83.76	14.62
AB00477644	DUB3	1	PY00001019	F012	691.00	72.72	-29.15
AB00477644	DUB3	1	PY00000207	F012	400.00	81.56	9.18
AB00477645	DUB3	1	PY00001012	F013	1362.00	16.66	-11.75
AB00477645	DUB3	1	PY00001019	F013	584.00	26.78	-9.55
AB00477645	DUB3	1	PY00000207	F013	529.00	7.49	-10.85
AB00477646	DUB3	1	PY00001012	F014	909.00	-7.95	33.87
AB00477646	DUB3	1	PY00001019	F014	493.00	13.56	20.85
AB00477646	DUB3	1	PY00000207	F014	379.00	51.80	24.85
AB00477647	DUSP8	1	PY00001012	F015	1391.00	39.96	-15.21
AB00477647	DUSP8	1	PY00001019	F015	683.00	58.05	-20.85
AB00477647	DUSP8	1	PY00000207	F015	597.00	67.25	-24.48
AB00477648	DUSP8	1	PY00001012	F016	948.00	-7.63	25.60
AB00477648	DUSP8	1	PY00001019	F016	568.00	16.38	7.79
AB00477648	DUSP8	1	PY00000207	F016	420.00	35.43	8.89
AB00477649	DUSP8	1	PY00001012	F017	1549.00	64.12	-27.82
AB00477649	DUSP8	1	PY00001019	F017	629.00	19.92	-7.29
AB00477649	DUSP8	1	PY00000207	F017	510.00	45.69	-8.52
AB00477650	DUSP8	1	PY00001012	F018	1526.00	42.13	-29.36
AB00477650	DUSP8	1	PY00001019	F018	664.00	14.23	-18.09
AB00477650	DUSP8	1	PY00000207	F018	489.00	20.76	-3.88
AB00477651	DVL2	1	PY00001012	F019	1308.00	37.19	-6.94
AB00477651	DVL2	1	PY00001019	F019	667.00	22.18	-19.35
AB00477651	DVL2	1	PY00000207	F019	538.00	91.38	-19.55
AB00477652	DVL2	1	PY00001012	F020	419.00	-31.00	71.89
AB00477652	DVL2	1	PY00001019	F020	408.00	-18.89	32.91
AB00477652	DVL2	1	PY00000207	F020	344.00	-1.21	33.26
AB00477653	DVL2	1	PY00001012	F021	1486.00	44.07	-25.99
AB00477653	DVL2	1	PY00001019	F021	592.00	12.87	-3.77
AB00477653	DVL2	1	PY00000207	F021	421.00	19.40	17.30
AB00477654	DVL2	1	PY00001012	F022	933.00	-24.21	29.06
AB00477654	DVL2	1	PY00001019	F022	443.00	6.75	29.15
AB00477654	DVL2	1	PY00000207	F022	354.00	-9.81	34.42
AB00477655	EDG5	1	PY00001012	G003	1463.00	33.31	-23.88
AB00477655	EDG5	1	PY00001019	G003	615.00	26.22	-13.07
AB00477655	EDG5	1	PY00000207	G003	581.00	16.24	-23.32
AB00477656	EDG5	1	PY00001012	G004	1222.00	18.01	-0.20

AB00477656	EDG5	1	PY00001019	G004	547.00	41.84	-0.25
AB00477656	EDG5	1	PY00000207	G004	621.00	25.73	-31.45
AB00477657	EDG5	1	PY00001012	G005	1311.00	20.20	-8.28
AB00477657	EDG5	1	PY00001019	G005	480.00	-23.93	17.34
AB00477657	EDG5	1	PY00000207	G005	510.00	-35.07	0.76
AB00477658	EDG5	1	PY00001012	G006	1223.00	-1.59	4.90
AB00477658	EDG5	1	PY00001019	G006	538.00	-19.64	6.03
AB00477658	EDG5	1	PY00000207	G006	523.00	39.90	-7.65
AB00477659	ELK3	1	PY00001012	G007	1253.00	18.61	-0.68
AB00477659	ELK3	1	PY00001019	G007	559.00	37.44	-0.75
AB00477659	ELK3	1	PY00000207	G007	562.00	28.63	-21.29
AB00477660	ELK3	1	PY00001012	G008	1152.00	75.61	1.34
AB00477660	ELK3	1	PY00001019	G008	517.00	-27.26	9.30
AB00477660	ELK3	1	PY00000207	G008	489.00	-21.66	-2.14
AB00477661	ELK3	1	PY00001012	G009	1288.00	32.31	-1.93
AB00477661	ELK3	1	PY00001019	G009	487.00	30.57	17.59
AB00477661	ELK3	1	PY00000207	G009	495.00	34.70	-0.11
AB00477662	ELK3	1	PY00001012	G010	1372.00	18.58	-14.54
AB00477662	ELK3	1	PY00001019	G010	544.00	9.48	-4.52
AB00477662	ELK3	1	PY00000207	G010	431.00	10.58	15.85
AB00477663	EPAS1	1	PY00001012	G011	1387.00	31.61	-17.14
AB00477663	EPAS1	1	PY00001019	G011	524.00	-14.23	7.29
AB00477663	EPAS1	1	PY00000207	G011	617.00	35.25	-37.54
AB00477664	EPAS1	1	PY00001012	G012	1523.00	36.58	-32.54
AB00477664	EPAS1	1	PY00001019	G012	648.00	4.70	-23.87
AB00477664	EPAS1	1	PY00000207	G012	626.00	-28.94	-30.58
AB00477665	EPAS1	1	PY00001012	G013	1240.00	-1.47	1.05
AB00477665	EPAS1	1	PY00001019	G013	516.00	-0.78	1.26
AB00477665	EPAS1	1	PY00000207	G013	562.00	-19.24	-21.29
AB00477666	EPAS1	1	PY00001012	G014	1486.00	56.95	-26.57
AB00477666	EPAS1	1	PY00001019	G014	601.00	20.81	-7.29
AB00477666	EPAS1	1	PY00000207	G014	638.00	64.31	-28.55
AB00477667	ETS2	1	PY00001012	G015	1197.00	6.70	3.46
AB00477667	ETS2	1	PY00001019	G015	436.00	-21.00	25.63
AB00477667	ETS2	1	PY00000207	G015	388.00	-59.75	27.17
AB00477668	ETS2	1	PY00001012	G016	1620.00	37.58	-43.90
AB00477668	ETS2	1	PY00001019	G016	529.00	-15.14	-0.75
AB00477668	ETS2	1	PY00000207	G016	447.00	-5.55	11.50
AB00477669	ETS2	1	PY00001012	G017	1270.00	4.82	-4.53
AB00477669	ETS2	1	PY00001019	G017	485.00	-8.15	20.35
AB00477669	ETS2	1	PY00000207	G017	552.00	27.57	-17.23
AB00477670	ETS2	1	PY00001012	G018	1140.00	75.54	8.27
AB00477670	ETS2	1	PY00001019	G018	457.00	18.06	22.11
AB00477670	ETS2	1	PY00000207	G018	546.00	109.74	-14.62
AB00477671	EYA1	1	PY00001012	G019	1473.00	22.26	-24.74
AB00477671	EYA1	1	PY00001019	G019	546.00	0.41	-3.02

AB00477671	EYA1	1	PY00000207	G019	485.00	47.17	-6.20
AB00477672	EYA1	1	PY00001012	G020	1104.00	1.06	14.24
AB00477672	EYA1	1	PY00001019	G020	418.00	-28.39	32.66
AB00477672	EYA1	1	PY00000207	G020	510.00	-14.33	-0.11
AB00477673	EYA1	1	PY00001012	G021	1442.00	23.16	-23.40
AB00477673	EYA1	1	PY00001019	G021	546.00	0.75	-1.26
AB00477673	EYA1	1	PY00000207	G021	540.00	49.41	-15.49
AB00477674	EYA1	1	PY00001012	G022	1435.00	11.84	-21.76
AB00477674	EYA1	1	PY00001019	G022	528.00	27.27	6.28
AB00477674	EYA1	1	PY00000207	G022	612.00	65.46	-33.19
AB00477675	FOSB	1	PY00001012	H003	1040.00	4.55	16.45
AB00477675	FOSB	1	PY00001019	H003	538.00	-26.16	4.27
AB00477675	FOSB	1	PY00000207	H003	527.00	-39.96	-12.88
AB00477676	FOSB	1	PY00001012	H004	1049.00	-13.47	14.91
AB00477676	FOSB	1	PY00001019	H004	532.00	-23.56	8.29
AB00477676	FOSB	1	PY00000207	H004	475.00	-26.39	6.86
AB00477677	FOSB	1	PY00001012	H005	1115.00	-32.85	11.35
AB00477677	FOSB	1	PY00001019	H005	522.00	-18.61	11.31
AB00477677	FOSB	1	PY00000207	H005	555.00	-12.32	-18.39
AB00477678	FOSB	1	PY00001012	H006	1165.00	15.27	5.58
AB00477678	FOSB	1	PY00001019	H006	608.00	2.77	-6.78
AB00477678	FOSB	1	PY00000207	H006	472.00	5.96	3.66
AB00477679	FRAP1	1	PY00001012	H007	1232.00	41.10	-2.99
AB00477679	FRAP1	1	PY00001019	H007	572.00	6.64	-7.04
AB00477679	FRAP1	1	PY00000207	H007	469.00	52.53	-2.72
AB00477680	FRAP1	1	PY00001012	H008	887.00	9.23	28.97
AB00477680	FRAP1	1	PY00001019	H008	546.00	22.39	2.01
AB00477680	FRAP1	1	PY00000207	H008	383.00	-7.62	23.10
AB00477681	FRAP1	1	PY00001012	H009	1073.00	-17.92	13.37
AB00477681	FRAP1	1	PY00001019	H009	667.00	15.36	-30.15
AB00477681	FRAP1	1	PY00000207	H009	528.00	14.45	-14.62
AB00477682	FRAP1	1	PY00001012	H010	1032.00	-2.78	15.01
AB00477682	FRAP1	1	PY00001019	H010	490.00	-21.38	15.83
AB00477682	FRAP1	1	PY00000207	H010	440.00	-30.83	8.60
AB00477683	GNG2	1	PY00001012	H011	1048.00	25.78	15.59
AB00477683	GNG2	1	PY00001019	H011	631.00	71.92	-11.31
AB00477683	GNG2	1	PY00000207	H011	498.00	51.56	1.34
AB00477684	GNG2	1	PY00001012	H012	1161.00	-14.05	3.07
AB00477684	GNG2	1	PY00001019	H012	700.00	-14.36	-30.40
AB00477684	GNG2	1	PY00000207	H012	395.00	-68.52	25.14
AB00477685	GNG2	1	PY00001012	H013	1361.00	10.68	-12.04
AB00477685	GNG2	1	PY00001019	H013	648.00	22.80	-15.58
AB00477685	GNG2	1	PY00000207	H013	574.00	-41.91	-25.06
AB00477686	GNG2	1	PY00001012	H014	1003.00	-25.43	21.94
AB00477686	GNG2	1	PY00001019	H014	602.00	-8.99	-12.06
AB00477686	GNG2	1	PY00000207	H014	539.00	-10.56	-12.88

AB00477687	GSH-2	1	PY00001012	H015	1232.00	1.53	-0.20
AB00477687	GSH-2	1	PY00001019	H015	596.00	-1.46	-5.03
AB00477687	GSH-2	1	PY00000207	H015	502.00	4.84	-5.62
AB00477688	GSH-2	1	PY00001012	H016	1106.00	-20.67	10.29
AB00477688	GSH-2	1	PY00001019	H016	518.00	2.20	5.78
AB00477688	GSH-2	1	PY00000207	H016	418.00	41.47	12.95
AB00477689	GSH-2	1	PY00001012	H017	1312.00	21.87	-10.88
AB00477689	GSH-2	1	PY00001019	H017	655.00	12.68	-19.10
AB00477689	GSH-2	1	PY00000207	H017	522.00	-39.53	-13.17
AB00477690	GSH-2	1	PY00001012	H018	1133.00	7.59	10.58
AB00477690	GSH-2	1	PY00001019	H018	524.00	-28.25	11.56
AB00477690	GSH-2	1	PY00000207	H018	400.00	-12.60	19.91
AB00477691	HBM	1	PY00001012	H019	1243.00	27.60	-3.37
AB00477691	HBM	1	PY00001019	H019	604.00	51.18	-12.06
AB00477691	HBM	1	PY00000207	H019	485.00	43.15	1.34
AB00477692	HBM	1	PY00001012	H020	1269.00	116.08	-7.03
AB00477692	HBM	1	PY00001019	H020	558.00	39.99	1.51
AB00477692	HBM	1	PY00000207	H020	470.00	-19.37	1.92
AB00477693	HBM	1	PY00001012	H021	1247.00	29.29	-3.18
AB00477693	HBM	1	PY00001019	H021	622.00	4.78	-10.80
AB00477693	HBM	1	PY00000207	H021	480.00	-9.11	5.69
AB00477694	HBM	1	PY00001012	H022	1340.00	27.32	-11.85
AB00477694	HBM	1	PY00001019	H022	647.00	24.20	-19.35
AB00477694	HBM	1	PY00000207	H022	489.00	45.06	-8.52
AB00477695	HIF1A	1	PY00001012	I003	1352.00	22.36	-12.62
AB00477695	HIF1A	1	PY00001019	I003	567.00	-6.50	-5.03
AB00477695	HIF1A	1	PY00000207	I003	559.00	-5.88	-21.00
AB00477696	HIF1A	1	PY00001012	I004	1494.00	34.95	-26.09
AB00477696	HIF1A	1	PY00001019	I004	323.00	-20.07	40.45
AB00477696	HIF1A	1	PY00000207	I004	566.00	31.49	-24.19
AB00477697	HIF1A	1	PY00001012	I005	1031.00	32.06	16.65
AB00477697	HIF1A	1	PY00001019	I005	507.00	-4.16	4.77
AB00477697	HIF1A	1	PY00000207	I005	607.00	1.41	-34.06
AB00477698	HIF1A	1	PY00001012	I006	1661.00	49.33	-43.90
AB00477698	HIF1A	1	PY00001019	I006	647.00	-0.56	-24.87
AB00477698	HIF1A	1	PY00000207	I006	588.00	8.39	-24.48
AB00477699	HOXB3	1	PY00001012	I007	1215.00	21.12	-1.93
AB00477699	HOXB3	1	PY00001019	I007	367.00	-37.92	41.71
AB00477699	HOXB3	1	PY00000207	I007	553.00	-22.79	-15.20
AB00477700	HOXB3	1	PY00001012	I008	1308.00	5.65	-9.53
AB00477700	HOXB3	1	PY00001019	I008	483.00	-15.09	15.83
AB00477700	HOXB3	1	PY00000207	I008	555.00	15.12	-23.32
AB00477701	HOXB3	1	PY00001012	I009	1200.00	1.04	6.44
AB00477701	HOXB3	1	PY00001019	I009	465.00	-24.14	21.86
AB00477701	HOXB3	1	PY00000207	I009	518.00	-21.07	-16.07
AB00477702	HOXB3	1	PY00001012	I010	1214.00	38.17	-1.45

AB00477702	HOXB3	1	PY00001019	I010	526.00	10.63	7.79
AB00477702	HOXB3	1	PY00000207	I010	601.00	13.76	-28.55
AB00477703	H-PLK	1	PY00001012	I011	901.00	47.98	32.62
AB00477703	H-PLK	1	PY00001019	I011	358.00	79.40	40.45
AB00477703	H-PLK	1	PY00000207	I011	527.00	55.02	-12.01
AB00477704	H-PLK	1	PY00001012	I012	1423.00	42.60	-14.73
AB00477704	H-PLK	1	PY00001019	I012	542.00	28.27	10.05
AB00477704	H-PLK	1	PY00000207	I012	479.00	40.39	10.05
AB00477705	H-PLK	1	PY00001012	I013	1378.00	50.53	-12.90
AB00477705	H-PLK	1	PY00001019	I013	456.00	8.69	22.61
AB00477705	H-PLK	1	PY00000207	I013	532.00	74.76	-14.62
AB00477706	H-PLK	1	PY00001012	I014	1321.00	58.95	-7.71
AB00477706	H-PLK	1	PY00001019	I014	581.00	42.70	-9.05
AB00477706	H-PLK	1	PY00000207	I014	472.00	-16.94	10.34
AB00477707	HSP90B1	1	PY00001012	I015	1406.00	3.24	-18.10
AB00477707	HSP90B1	1	PY00001019	I015	510.00	-13.65	9.80
AB00477707	HSP90B1	1	PY00000207	I015	559.00	52.59	-11.14
AB00477708	HSP90B1	1	PY00001012	I016	1444.00	23.30	-22.24
AB00477708	HSP90B1	1	PY00001019	I016	556.00	3.60	0.00
AB00477708	HSP90B1	1	PY00000207	I016	434.00	11.13	8.31
AB00477709	HSP90B1	1	PY00001012	I017	1556.00	17.46	-27.92
AB00477709	HSP90B1	1	PY00001019	I017	541.00	-31.71	12.31
AB00477709	HSP90B1	1	PY00000207	I017	658.00	67.29	-37.25
AB00477710	HSP90B1	1	PY00001012	I018	1360.00	48.64	-10.88
AB00477710	HSP90B1	1	PY00001019	I018	455.00	-10.82	23.87
AB00477710	HSP90B1	1	PY00000207	I018	555.00	24.53	-15.78
AB00477711	HSPA5BP1	1	PY00001012	I019	1490.00	60.03	-27.92
AB00477711	HSPA5BP1	1	PY00001019	I019	512.00	22.65	8.29
AB00477711	HSPA5BP1	1	PY00000207	I019	546.00	15.82	-16.65
AB00477712	HSPA5BP1	1	PY00001012	I020	1551.00	47.37	-31.48
AB00477712	HSPA5BP1	1	PY00001019	I020	332.00	3.88	41.46
AB00477712	HSPA5BP1	1	PY00000207	I020	532.00	54.39	-15.78
AB00477713	HSPA5BP1	1	PY00001012	I021	1272.00	8.14	-3.18
AB00477713	HSPA5BP1	1	PY00001019	I021	399.00	-14.69	31.16
AB00477713	HSPA5BP1	1	PY00000207	I021	427.00	-1.74	15.85
AB00477714	HSPA5BP1	1	PY00001012	I022	1428.00	46.36	-20.12
AB00477714	HSPA5BP1	1	PY00001019	I022	613.00	45.20	-16.08
AB00477714	HSPA5BP1	1	PY00000207	I022	612.00	64.90	-30.87
AB00477715	HSPA6	1	PY00001012	J003	1095.00	-28.26	13.66
AB00477715	HSPA6	1	PY00001019	J003	515.00	16.33	12.56
AB00477715	HSPA6	1	PY00000207	J003	506.00	-17.08	-11.14
AB00477716	HSPA6	1	PY00001012	J004	1106.00	-33.61	17.03
AB00477716	HSPA6	1	PY00001019	J004	633.00	-23.30	-13.32
AB00477716	HSPA6	1	PY00000207	J004	437.00	-33.07	6.86
AB00477717	HSPA6	1	PY00001012	J005	1334.00	-4.19	-10.88
AB00477717	HSPA6	1	PY00001019	J005	580.00	-28.74	-1.26

AB00477717	HSPA6	1	PY00000207	J005	500.00	-27.52	-7.65
AB00477718	HSPA6	1	PY00001012	J006	1175.00	-26.87	6.54
AB00477718	HSPA6	1	PY00001019	J006	580.00	-11.22	-6.03
AB00477718	HSPA6	1	PY00000207	J006	434.00	-0.15	-0.40
AB00477719	IRAK2	1	PY00001012	J007	1298.00	15.23	-7.32
AB00477719	IRAK2	1	PY00001019	J007	570.00	-8.15	-2.51
AB00477719	IRAK2	1	PY00000207	J007	523.00	-28.83	-8.52
AB00477720	IRAK2	1	PY00001012	J008	1195.00	4.37	0.86
AB00477720	IRAK2	1	PY00001019	J008	614.00	-6.36	-10.55
AB00477720	IRAK2	1	PY00000207	J008	502.00	-23.41	-4.17
AB00477721	IRAK2	1	PY00001012	J009	1206.00	-12.93	0.76
AB00477721	IRAK2	1	PY00001019	J009	507.00	-14.69	15.58
AB00477721	IRAK2	1	PY00000207	J009	465.00	32.08	3.37
AB00477722	IRAK2	1	PY00001012	J010	1311.00	17.85	-8.76
AB00477722	IRAK2	1	PY00001019	J010	674.00	-6.91	-27.39
AB00477722	IRAK2	1	PY00000207	J010	467.00	-63.49	2.50
AB00477723	KIF15	1	PY00001012	J011	984.00	-12.55	21.17
AB00477723	KIF15	1	PY00001019	J011	605.00	20.74	-2.26
AB00477723	KIF15	1	PY00000207	J011	446.00	-22.74	11.50
AB00477724	KIF15	1	PY00001012	J012	711.00	-25.01	47.83
AB00477724	KIF15	1	PY00001019	J012	550.00	11.11	14.57
AB00477724	KIF15	1	PY00000207	J012	412.00	-15.71	17.30
AB00477725	KIF15	1	PY00001012	J013	1285.00	16.91	-4.72
AB00477725	KIF15	1	PY00001019	J013	673.00	53.57	-27.39
AB00477725	KIF15	1	PY00000207	J013	495.00	-45.26	2.21
AB00477726	KIF15	1	PY00001012	J014	1252.00	19.62	-5.11
AB00477726	KIF15	1	PY00001019	J014	566.00	-7.99	-2.26
AB00477726	KIF15	1	PY00000207	J014	428.00	15.91	6.86
AB00477727	KIF3A	1	PY00001012	J015	1091.00	-12.53	12.03
AB00477727	KIF3A	1	PY00001019	J015	534.00	18.95	2.01
AB00477727	KIF3A	1	PY00000207	J015	387.00	55.67	23.69
AB00477728	KIF3A	1	PY00001012	J016	1184.00	7.66	1.63
AB00477728	KIF3A	1	PY00001019	J016	727.00	14.53	-37.94
AB00477728	KIF3A	1	PY00000207	J016	494.00	-9.93	-5.33
AB00477729	KIF3A	1	PY00001012	J017	1259.00	11.40	-2.60
AB00477729	KIF3A	1	PY00001019	J017	601.00	37.56	-6.28
AB00477729	KIF3A	1	PY00000207	J017	464.00	-25.71	5.98
AB00477730	KIF3A	1	PY00001012	J018	1446.00	67.52	-21.47
AB00477730	KIF3A	1	PY00001019	J018	639.00	49.54	-20.60
AB00477730	KIF3A	1	PY00000207	J018	513.00	13.03	-12.59
AB00477731	MAP3K2	1	PY00001012	J019	1360.00	46.20	-14.93
AB00477731	MAP3K2	1	PY00001019	J019	783.00	50.96	-52.51
AB00477731	MAP3K2	1	PY00000207	J019	594.00	-8.44	-29.42
AB00477732	MAP3K2	1	PY00001012	J020	1559.00	27.42	-30.23
AB00477732	MAP3K2	1	PY00001019	J020	631.00	71.41	-19.35
AB00477732	MAP3K2	1	PY00000207	J020	461.00	-3.84	10.34

AB00477733	MAP3K2	1	PY00001012	J021	1239.00	17.62	-2.99
AB00477733	MAP3K2	1	PY00001019	J021	509.00	7.88	7.04
AB00477733	MAP3K2	1	PY00000207	J021	504.00	43.30	-7.07
AB00477734	MAP3K2	1	PY00001012	J022	1285.00	22.19	-8.76
AB00477734	MAP3K2	1	PY00001019	J022	603.00	9.98	-12.56
AB00477734	MAP3K2	1	PY00000207	J022	473.00	36.97	6.57
AB00477735	MAP3K6	1	PY00001012	K003	1306.00	-27.19	-1.64
AB00477735	MAP3K6	1	PY00001019	K003	481.00	6.80	16.58
AB00477735	MAP3K6	1	PY00000207	K003	414.00	-42.10	16.72
AB00477736	MAP3K6	1	PY00001012	K004	1112.00	0.53	10.87
AB00477736	MAP3K6	1	PY00001019	K004	385.00	-13.74	34.67
AB00477736	MAP3K6	1	PY00000207	K004	549.00	11.14	-14.33
AB00477737	MAP3K6	1	PY00001012	K005	1319.00	41.99	-10.21
AB00477737	MAP3K6	1	PY00001019	K005	518.00	8.69	10.30
AB00477737	MAP3K6	1	PY00000207	K005	522.00	14.42	-9.68
AB00477738	MAP3K6	1	PY00001012	K006	1204.00	3.05	4.42
AB00477738	MAP3K6	1	PY00001019	K006	610.00	21.74	-12.81
AB00477738	MAP3K6	1	PY00000207	K006	559.00	48.64	-12.59
AB00477739	MAPK12	1	PY00001012	K007	1443.00	11.80	-21.28
AB00477739	MAPK12	1	PY00001019	K007	543.00	-22.80	3.02
AB00477739	MAPK12	1	PY00000207	K007	425.00	22.47	3.66
AB00477740	MAPK12	1	PY00001012	K008	1496.00	31.90	-26.86
AB00477740	MAPK12	1	PY00001019	K008	435.00	31.21	23.62
AB00477740	MAPK12	1	PY00000207	K008	675.00	64.63	-53.50
AB00477741	MAPK12	1	PY00001012	K009	791.00	-37.63	45.62
AB00477741	MAPK12	1	PY00001019	K009	425.00	-34.27	31.16
AB00477741	MAPK12	1	PY00000207	K009	424.00	-18.92	18.17
AB00477742	MAPK12	1	PY00001012	K010	924.00	13.05	25.40
AB00477742	MAPK12	1	PY00001019	K010	671.00	44.09	-25.38
AB00477742	MAPK12	1	PY00000207	K010	774.00	7.92	-73.81
AB00477743	MAPK13	1	PY00001012	K011	1086.00	2.38	15.11
AB00477743	MAPK13	1	PY00001019	K011	480.00	14.97	18.84
AB00477743	MAPK13	1	PY00000207	K011	485.00	25.13	8.60
AB00477744	MAPK13	1	PY00001012	K012	1400.00	19.73	-17.33
AB00477744	MAPK13	1	PY00001019	K012	687.00	24.54	-34.17
AB00477744	MAPK13	1	PY00000207	K012	514.00	33.49	-15.78
AB00477745	MAPK13	1	PY00001012	K013	1432.00	12.16	-16.56
AB00477745	MAPK13	1	PY00001019	K013	479.00	-13.36	19.85
AB00477745	MAPK13	1	PY00000207	K013	473.00	-0.10	-0.98
AB00477746	MAPK13	1	PY00001012	K014	1348.00	16.30	-9.44
AB00477746	MAPK13	1	PY00001019	K014	559.00	22.87	1.01
AB00477746	MAPK13	1	PY00000207	K014	547.00	4.35	-10.26
AB00477747	MGC54289	1	PY00001012	K015	913.00	-32.85	35.51
AB00477747	MGC54289	1	PY00001019	K015	334.00	-41.82	49.75
AB00477747	MGC54289	1	PY00000207	K015	389.00	-39.82	27.17
AB00477748	MGC54289	1	PY00001012	K016	1322.00	-5.47	-2.99

AB00477748	MGC54289	1	PY00001019	K016	578.00	37.62	-2.76
AB00477748	MGC54289	1	PY00000207	K016	592.00	37.15	-27.38
AB00477749	MGC54289	1	PY00001012	K017	1483.00	44.65	-24.17
AB00477749	MGC54289	1	PY00001019	K017	488.00	-6.52	10.05
AB00477749	MGC54289	1	PY00000207	K017	618.00	45.10	-43.92
AB00477750	MGC54289	1	PY00001012	K018	955.00	-30.46	25.31
AB00477750	MGC54289	1	PY00001019	K018	606.00	29.82	-13.32
AB00477750	MGC54289	1	PY00000207	K018	611.00	21.04	-23.03
AB00477751	MMP7	1	PY00001012	K019	1111.00	-9.61	15.20
AB00477751	MMP7	1	PY00001019	K019	421.00	-12.03	34.92
AB00477751	MMP7	1	PY00000207	K019	496.00	-2.47	-4.46
AB00477752	MMP7	1	PY00001012	K020	1147.00	1.53	3.65
AB00477752	MMP7	1	PY00001019	K020	480.00	5.60	11.06
AB00477752	MMP7	1	PY00000207	K020	666.00	32.12	-46.83
AB00477753	MMP7	1	PY00001012	K021	1352.00	-5.13	-9.92
AB00477753	MMP7	1	PY00001019	K021	564.00	-15.40	3.77
AB00477753	MMP7	1	PY00000207	K021	500.00	19.34	4.53
AB00477754	MMP7	1	PY00001012	K022	1302.00	3.96	-7.80
AB00477754	MMP7	1	PY00001019	K022	570.00	-8.31	-2.76
AB00477754	MMP7	1	PY00000207	K022	533.00	31.40	-2.43
AB00477755	MRLC2	1	PY00001012	L003	1212.00	46.96	-1.93
AB00477755	MRLC2	1	PY00001019	L003	587.00	41.17	-1.26
AB00477755	MRLC2	1	PY00000207	L003	486.00	38.92	-0.98
AB00477756	MRLC2	1	PY00001012	L004	1294.00	38.67	-9.73
AB00477756	MRLC2	1	PY00001019	L004	611.00	35.47	-14.07
AB00477756	MRLC2	1	PY00000207	L004	543.00	-1.79	-22.16
AB00477757	MRLC2	1	PY00001012	L005	1320.00	21.36	-14.73
AB00477757	MRLC2	1	PY00001019	L005	637.00	44.74	-8.79
AB00477757	MRLC2	1	PY00000207	L005	520.00	17.23	-9.97
AB00477758	MRLC2	1	PY00001012	L006	1183.00	39.97	4.33
AB00477758	MRLC2	1	PY00001019	L006	702.00	52.05	-33.92
AB00477758	MRLC2	1	PY00000207	L006	454.00	62.26	13.82
AB00477759	NOTCH3	1	PY00001012	L007	1318.00	40.64	-9.92
AB00477759	NOTCH3	1	PY00001019	L007	697.00	44.85	-28.64
AB00477759	NOTCH3	1	PY00000207	L007	570.00	62.21	-23.32
AB00477760	NOTCH3	1	PY00001012	L008	1270.00	1.59	-7.80
AB00477760	NOTCH3	1	PY00001019	L008	691.00	11.04	-23.12
AB00477760	NOTCH3	1	PY00000207	L008	385.00	-69.11	20.78
AB00477761	NOTCH3	1	PY00001012	L009	1146.00	-35.32	7.98
AB00477761	NOTCH3	1	PY00001019	L009	615.00	-25.42	-9.30
AB00477761	NOTCH3	1	PY00000207	L009	484.00	-42.53	1.05
AB00477762	NOTCH3	1	PY00001012	L010	1214.00	12.80	0.19
AB00477762	NOTCH3	1	PY00001019	L010	708.00	9.93	-33.42
AB00477762	NOTCH3	1	PY00000207	L010	551.00	20.91	-21.58
AB00477763	NRN1	1	PY00001012	L011	1131.00	-17.31	7.21
AB00477763	NRN1	1	PY00001019	L011	659.00	-6.41	-12.06

AB00477763	NRN1	1	PY00000207	L011	356.00	-59.88	32.39
AB00477764	NRN1	1	PY00001012	L012	1121.00	27.44	9.52
AB00477764	NRN1	1	PY00001019	L012	563.00	44.75	-2.26
AB00477764	NRN1	1	PY00000207	L012	412.00	-29.84	20.78
AB00477765	NRN1	1	PY00001012	L013	1365.00	-37.44	-9.63
AB00477765	NRN1	1	PY00001019	L013	773.00	4.12	-48.99
AB00477765	NRN1	1	PY00000207	L013	521.00	23.89	-11.72
AB00477766	NRN1	1	PY00001012	L014	1256.00	-0.41	-4.14
AB00477766	NRN1	1	PY00001019	L014	599.00	-0.30	-4.02
AB00477766	NRN1	1	PY00000207	L014	515.00	35.27	-12.01
AB00477767	PAX2	1	PY00001012	L015	1030.00	10.55	17.99
AB00477767	PAX2	1	PY00001019	L015	607.00	48.56	-3.02
AB00477767	PAX2	1	PY00000207	L015	453.00	17.66	2.21
AB00477768	PAX2	1	PY00001012	L016	1203.00	17.66	1.63
AB00477768	PAX2	1	PY00001019	L016	632.00	49.86	-11.31
AB00477768	PAX2	1	PY00000207	L016	551.00	89.11	-23.32
AB00477769	PAX2	1	PY00001012	L017	1339.00	34.73	-9.73
AB00477769	PAX2	1	PY00001019	L017	619.00	23.25	-11.06
AB00477769	PAX2	1	PY00000207	L017	408.00	51.09	15.56
AB00477770	PAX2	1	PY00001012	L018	1183.00	-2.90	3.75
AB00477770	PAX2	1	PY00001019	L018	599.00	8.95	-9.30
AB00477770	PAX2	1	PY00000207	L018	360.00	13.02	22.52
AB00477771	PDK1	1	PY00001012	L019	1392.00	31.68	-18.78
AB00477771	PDK1	1	PY00001019	L019	634.00	33.90	-10.80
AB00477771	PDK1	1	PY00000207	L019	529.00	13.87	-14.04
AB00477772	PDK1	1	PY00001012	L020	1429.00	21.68	-20.32
AB00477772	PDK1	1	PY00001019	L020	601.00	28.55	-13.32
AB00477772	PDK1	1	PY00000207	L020	513.00	57.01	-5.33
AB00477773	PDK1	1	PY00001012	L021	1282.00	14.62	-5.30
AB00477773	PDK1	1	PY00001019	L021	620.00	58.20	-20.35
AB00477773	PDK1	1	PY00000207	L021	422.00	23.97	14.98
AB00477774	PDK1	1	PY00001012	L022	1331.00	11.07	-12.81
AB00477774	PDK1	1	PY00001019	L022	607.00	44.37	-12.81
AB00477774	PDK1	1	PY00000207	L022	452.00	36.16	5.11
AB00477775	PDLIM7	1	PY00001012	M003	1361.00	-3.96	-13.96
AB00477775	PDLIM7	1	PY00001019	M003	357.00	-16.32	35.93
AB00477775	PDLIM7	1	PY00000207	M003	580.00	34.58	-20.13
AB00477776	PDLIM7	1	PY00001012	M004	1331.00	31.45	-8.28
AB00477776	PDLIM7	1	PY00001019	M004	616.00	25.20	-11.31
AB00477776	PDLIM7	1	PY00000207	M004	599.00	17.88	-23.90
AB00477777	PDLIM7	1	PY00001012	M005	1535.00	35.24	-31.10
AB00477777	PDLIM7	1	PY00001019	M005	566.00	43.16	-2.01
AB00477777	PDLIM7	1	PY00000207	M005	685.00	36.64	-58.72
AB00477778	PDLIM7	1	PY00001012	M006	1424.00	-1.93	-16.75
AB00477778	PDLIM7	1	PY00001019	M006	568.00	21.93	-5.53
AB00477778	PDLIM7	1	PY00000207	M006	585.00	14.70	-25.93

AB00477779	PHYHIPL	1	PY00001012	M007	1368.00	37.63	-16.56
AB00477779	PHYHIPL	1	PY00001019	M007	545.00	-19.88	2.01
AB00477779	PHYHIPL	1	PY00000207	M007	597.00	-28.76	-30.29
AB00477780	PHYHIPL	1	PY00001012	M008	1467.00	59.04	-24.84
AB00477780	PHYHIPL	1	PY00001019	M008	717.00	16.32	-38.94
AB00477780	PHYHIPL	1	PY00000207	M008	667.00	1.00	-45.67
AB00477781	PHYHIPL	1	PY00001012	M009	1021.00	3.85	19.24
AB00477781	PHYHIPL	1	PY00001019	M009	462.00	-10.30	13.82
AB00477781	PHYHIPL	1	PY00000207	M009	520.00	86.79	-9.39
AB00477782	PHYHIPL	1	PY00001012	M010	1164.00	-14.34	11.54
AB00477782	PHYHIPL	1	PY00001019	M010	417.00	-25.73	40.45
AB00477782	PHYHIPL	1	PY00000207	M010	458.00	8.11	8.31
AB00477783	POU4F1	1	PY00001012	M011	1467.00	45.39	-22.91
AB00477783	POU4F1	1	PY00001019	M011	504.00	-29.09	15.33
AB00477783	POU4F1	1	PY00000207	M011	545.00	-5.53	-11.43
AB00477784	POU4F1	1	PY00001012	M012	1516.00	61.04	-29.94
AB00477784	POU4F1	1	PY00001019	M012	591.00	17.48	-8.04
AB00477784	POU4F1	1	PY00000207	M012	824.00	45.70	-84.84
AB00477785	POU4F1	1	PY00001012	M013	1265.00	14.67	-0.20
AB00477785	POU4F1	1	PY00001019	M013	406.00	1.82	30.90
AB00477785	POU4F1	1	PY00000207	M013	552.00	-7.07	-18.39
AB00477786	POU4F1	1	PY00001012	M014	1447.00	31.30	-13.87
AB00477786	POU4F1	1	PY00001019	M014	557.00	4.67	0.75
AB00477786	POU4F1	1	PY00000207	M014	530.00	41.41	-7.94
AB00477787	PRAF2	1	PY00001012	M015	1409.00	17.08	-11.65
AB00477787	PRAF2	1	PY00001019	M015	497.00	-10.73	17.84
AB00477787	PRAF2	1	PY00000207	M015	451.00	13.48	3.37
AB00477788	PRAF2	1	PY00001012	M016	1383.00	47.26	-13.96
AB00477788	PRAF2	1	PY00001019	M016	558.00	34.07	-3.02
AB00477788	PRAF2	1	PY00000207	M016	564.00	62.68	-17.81
AB00477789	PRAF2	1	PY00001012	M017	1665.00	46.26	-41.39
AB00477789	PRAF2	1	PY00001019	M017	580.00	-8.25	-10.05
AB00477789	PRAF2	1	PY00000207	M017	563.00	85.68	-24.77
AB00477790	PRAF2	1	PY00001012	M018	1253.00	9.70	-5.68
AB00477790	PRAF2	1	PY00001019	M018	595.00	26.51	-5.03
AB00477790	PRAF2	1	PY00000207	M018	544.00	22.20	-17.81
AB00477791	PRB1	1	PY00001012	M019	1468.00	46.33	-23.68
AB00477791	PRB1	1	PY00001019	M019	573.00	26.55	-4.02
AB00477791	PRB1	1	PY00000207	M019	526.00	-7.81	-8.52
AB00477792	PRB1	1	PY00001012	M020	1009.00	-15.81	19.34
AB00477792	PRB1	1	PY00001019	M020	561.00	-36.02	4.77
AB00477792	PRB1	1	PY00000207	M020	546.00	-14.47	-11.72
AB00477793	PRB1	1	PY00001012	M021	1675.00	30.57	-42.45
AB00477793	PRB1	1	PY00001019	M021	566.00	17.49	-3.27
AB00477793	PRB1	1	PY00000207	M021	667.00	29.94	-52.05
AB00477794	PRB1	1	PY00001012	M022	1005.00	11.44	18.19

AB00477794	PRB1	1	PY00001019	M022	656.00	29.02	-19.85
AB00477794	PRB1	1	PY00000207	M022	586.00	67.95	-22.45
AB00477795	PRES	1	PY00001012	N003	1078.00	-13.17	14.62
AB00477795	PRES	1	PY00001019	N003	549.00	45.04	-2.01
AB00477795	PRES	1	PY00000207	N003	485.00	-65.13	6.57
AB00477796	PRES	1	PY00001012	N004	1294.00	-0.43	-6.84
AB00477796	PRES	1	PY00001019	N004	523.00	-28.47	5.78
AB00477796	PRES	1	PY00000207	N004	521.00	-19.14	-12.88
AB00477797	PRES	1	PY00001012	N005	952.00	-36.19	25.50
AB00477797	PRES	1	PY00001019	N005	645.00	-20.42	-15.33
AB00477797	PRES	1	PY00000207	N005	498.00	15.48	-3.30
AB00477798	PRES	1	PY00001012	N006	964.00	-27.39	26.94
AB00477798	PRES	1	PY00001019	N006	479.00	-41.10	19.60
AB00477798	PRES	1	PY00000207	N006	423.00	-31.86	15.27
AB00477799	PSMA1	1	PY00001012	N007	1137.00	-7.84	5.58
AB00477799	PSMA1	1	PY00001019	N007	514.00	-41.32	10.05
AB00477799	PSMA1	1	PY00000207	N007	460.00	-1.46	9.76
AB00477800	PSMA1	1	PY00001012	N008	1206.00	4.79	3.65
AB00477800	PSMA1	1	PY00001019	N008	614.00	-7.68	-11.31
AB00477800	PSMA1	1	PY00000207	N008	490.00	-14.88	-0.69
AB00477801	PSMA1	1	PY00001012	N009	890.00	-34.65	31.66
AB00477801	PSMA1	1	PY00001019	N009	527.00	-6.52	20.85
AB00477801	PSMA1	1	PY00000207	N009	488.00	29.57	6.57
AB00477802	PSMA1	1	PY00001012	N010	996.00	-34.12	18.47
AB00477802	PSMA1	1	PY00001019	N010	580.00	1.61	-2.01
AB00477802	PSMA1	1	PY00000207	N010	427.00	1.89	16.72
AB00477803	RARG	1	PY00001012	N011	949.00	4.18	23.96
AB00477803	RARG	1	PY00001019	N011	533.00	-9.73	7.04
AB00477803	RARG	1	PY00000207	N011	517.00	-53.36	-7.94
AB00477804	RARG	1	PY00001012	N012	1118.00	-21.94	8.85
AB00477804	RARG	1	PY00001019	N012	490.00	-39.09	20.10
AB00477804	RARG	1	PY00000207	N012	392.00	-59.67	28.62
AB00477805	RARG	1	PY00001012	N013	846.00	-36.88	37.05
AB00477805	RARG	1	PY00001019	N013	530.00	3.55	13.07
AB00477805	RARG	1	PY00000207	N013	397.00	-25.86	19.62
AB00477806	RARG	1	PY00001012	N014	823.00	5.12	39.94
AB00477806	RARG	1	PY00001019	N014	525.00	15.84	19.10
AB00477806	RARG	1	PY00000207	N014	372.00	-21.54	31.23
AB00477807	RHOA	1	PY00001012	N015	1152.00	2.35	6.83
AB00477807	RHOA	1	PY00001019	N015	643.00	1.82	-17.09
AB00477807	RHOA	1	PY00000207	N015	455.00	-15.86	6.27
AB00477808	RHOA	1	PY00001012	N016	1221.00	1.57	1.15
AB00477808	RHOA	1	PY00001019	N016	661.00	4.17	-21.61
AB00477808	RHOA	1	PY00000207	N016	519.00	44.04	-9.68
AB00477809	RHOA	1	PY00001012	N017	1438.00	32.64	-21.37
AB00477809	RHOA	1	PY00001019	N017	703.00	70.67	-29.65

AB00477809	RHOA	1	PY00000207	N017	443.00	11.36	14.69
AB00477810	RHOA	1	PY00001012	N018	1204.00	12.79	1.53
AB00477810	RHOA	1	PY00001019	N018	600.00	45.07	-4.02
AB00477810	RHOA	1	PY00000207	N018	495.00	69.26	2.21
AB00477811	RHOC	1	PY00001019	N019	554.00	28.46	8.79
AB00477811	RHOC	1	PY00000207	N019	408.00	47.53	20.20
AB00477812	RHOC	1	PY00001012	N020	1132.00	46.93	8.18
AB00477812	RHOC	1	PY00001019	N020	536.00	9.93	11.06
AB00477812	RHOC	1	PY00000207	N020	431.00	-18.09	12.95
AB00477813	RHOC	1	PY00001012	N021	1051.00	-12.05	15.39
AB00477813	RHOC	1	PY00001019	N021	678.00	37.39	-25.13
AB00477813	RHOC	1	PY00000207	N021	458.00	-6.12	7.73
AB00477814	RHOC	1	PY00001012	N022	1263.00	13.34	-2.03
AB00477814	RHOC	1	PY00001019	N022	520.00	-9.01	11.81
AB00477814	RHOC	1	PY00000207	N022	476.00	35.92	-0.69
AB00477815	RPS27A	1	PY00001012	O003	1057.00	-11.85	17.80
AB00477815	RPS27A	1	PY00001019	O003	457.00	-20.31	19.35
AB00477815	RPS27A	1	PY00000207	O003	519.00	-5.72	-2.43
AB00477816	RPS27A	1	PY00001012	O004	1006.00	-21.86	24.63
AB00477816	RPS27A	1	PY00001019	O004	437.00	-27.84	27.14
AB00477816	RPS27A	1	PY00000207	O004	469.00	-28.48	11.21
AB00477817	RPS27A	1	PY00001012	O005	1453.00	7.46	-19.45
AB00477817	RPS27A	1	PY00001019	O005	578.00	-10.93	-5.53
AB00477817	RPS27A	1	PY00000207	O005	572.00	-54.61	-20.13
AB00477818	RPS27A	1	PY00001012	O006	935.00	-27.62	28.87
AB00477818	RPS27A	1	PY00001019	O006	483.00	-34.62	21.86
AB00477818	RPS27A	1	PY00000207	O006	488.00	-59.14	8.02
AB00477819	RPS6	1	PY00001012	O007	913.00	-17.35	30.79
AB00477819	RPS6	1	PY00001019	O007	396.00	-43.52	40.45
AB00477819	RPS6	1	PY00000207	O007	450.00	-52.06	15.56
AB00477820	RPS6	1	PY00001012	O008	859.00	-44.42	40.71
AB00477820	RPS6	1	PY00001019	O008	446.00	-46.27	32.66
AB00477820	RPS6	1	PY00000207	O008	390.00	-80.02	35.29
AB00477821	RPS6	1	PY00001012	O009	1059.00	-6.88	18.09
AB00477821	RPS6	1	PY00001019	O009	439.00	5.17	25.88
AB00477821	RPS6	1	PY00000207	O009	460.00	35.22	15.85
AB00477822	RPS6	1	PY00001012	O010	833.00	-47.11	38.98
AB00477822	RPS6	1	PY00001019	O010	427.00	-34.50	36.93
AB00477822	RPS6	1	PY00000207	O010	510.00	2.91	0.47
AB00477823	SATB1	1	PY00001012	O011	845.00	4.16	28.87
AB00477823	SATB1	1	PY00001019	O011	367.00	3.97	35.93
AB00477823	SATB1	1	PY00000207	O011	657.00	13.81	-39.57
AB00477824	SATB1	1	PY00001012	O012	1375.00	32.63	-13.29
AB00477824	SATB1	1	PY00001019	O012	618.00	0.89	-7.54
AB00477824	SATB1	1	PY00000207	O012	548.00	-34.31	-9.97
AB00477825	SATB1	1	PY00001012	O013	1462.00	22.92	-16.85

AB00477825	SATB1	1	PY00001019	O013	487.00	11.86	15.33
AB00477825	SATB1	1	PY00000207	O013	642.00	-36.46	-37.25
AB00477826	SATB1	1	PY00001012	O014	1390.00	16.44	-16.47
AB00477826	SATB1	1	PY00001019	O014	492.00	34.57	13.07
AB00477826	SATB1	1	PY00000207	O014	645.00	6.37	-38.41
AB00477827	SERPINB2	1	PY00001012	O015	1412.00	3.83	-16.08
AB00477827	SERPINB2	1	PY00001019	O015	478.00	17.40	17.84
AB00477827	SERPINB2	1	PY00000207	O015	623.00	-41.75	-33.77
AB00477828	SERPINB2	1	PY00001012	O016	1429.00	20.55	-20.80
AB00477828	SERPINB2	1	PY00001019	O016	480.00	-22.15	14.57
AB00477828	SERPINB2	1	PY00000207	O016	579.00	-17.29	-22.45
AB00477829	SERPINB2	1	PY00001012	O017	1520.00	39.26	-30.13
AB00477829	SERPINB2	1	PY00001019	O017	493.00	-2.09	18.34
AB00477829	SERPINB2	1	PY00000207	O017	498.00	-63.77	0.47
AB00477830	SERPINB2	1	PY00001012	O018	1395.00	17.14	-16.95
AB00477830	SERPINB2	1	PY00001019	O018	655.00	44.50	-20.85
AB00477830	SERPINB2	1	PY00000207	O018	610.00	-15.17	-29.13
AB00477831	SFRS3	1	PY00001012	O019	1109.00	-35.93	14.72
AB00477831	SFRS3	1	PY00001019	O019	421.00	-17.82	28.14
AB00477831	SFRS3	1	PY00000207	O019	549.00	-32.22	-10.55
AB00477832	SFRS3	1	PY00001019	O020	513.00	-22.08	12.81
AB00477832	SFRS3	1	PY00000207	O020	576.00	-3.53	-15.78
AB00477833	SFRS3	1	PY00001012	O021	1268.00	10.64	-5.40
AB00477833	SFRS3	1	PY00001019	O021	476.00	-0.91	9.05
AB00477833	SFRS3	1	PY00000207	O021	510.00	6.71	1.34
AB00477834	SFRS3	1	PY00001012	O022	1431.00	21.41	-23.88
AB00477834	SFRS3	1	PY00001019	O022	643.00	22.18	-18.34
AB00477834	SFRS3	1	PY00000207	O022	637.00	79.58	-37.83
AB00477835	SIK2	1	PY00001012	P003	1025.00	-1.29	16.84
AB00477835	SIK2	1	PY00001019	P003	564.00	31.52	7.79
AB00477835	SIK2	1	PY00000207	P003	486.00	-59.90	3.95
AB00477836	SIK2	1	PY00001012	P004	1404.00	57.45	-18.49
AB00477836	SIK2	1	PY00001019	P004	719.00	57.37	-42.46
AB00477836	SIK2	1	PY00000207	P004	464.00	-55.80	4.82
AB00477837	SIK2	1	PY00001012	P005	1210.00	40.60	-1.93
AB00477837	SIK2	1	PY00001019	P005	703.00	-7.27	-19.35
AB00477837	SIK2	1	PY00000207	P005	483.00	-49.78	-3.88
AB00477838	SIK2	1	PY00001012	P006	844.00	-37.90	37.72
AB00477838	SIK2	1	PY00001019	P006	505.00	-44.23	32.41
AB00477838	SIK2	1	PY00000207	P006	340.00	-73.74	38.77
AB00477839	SMO	1	PY00001012	P007	1014.00	26.76	17.22
AB00477839	SMO	1	PY00001019	P007	592.00	-13.88	-0.25
AB00477839	SMO	1	PY00000207	P007	481.00	31.09	-14.33
AB00477840	SMO	1	PY00001012	P008	1429.00	58.99	-21.09
AB00477840	SMO	1	PY00001019	P008	618.00	4.54	-9.30
AB00477840	SMO	1	PY00000207	P008	467.00	27.65	-9.68

AB00477841	SMO	1	PY00001012	P009	970.00	-5.37	21.46
AB00477841	SMO	1	PY00001019	P009	682.00	42.38	-15.58
AB00477841	SMO	1	PY00000207	P009	475.00	3.11	-3.59
AB00477842	SMO	1	PY00001012	P010	1369.00	3.85	-13.87
AB00477842	SMO	1	PY00001019	P010	653.00	-4.91	-20.85
AB00477842	SMO	1	PY00000207	P010	451.00	-1.50	6.57
AB00477843	SNAI2	1	PY00001012	P011	918.00	-28.56	26.08
AB00477843	SNAI2	1	PY00001019	P011	671.00	2.36	-11.81
AB00477843	SNAI2	1	PY00000207	P011	459.00	-43.75	3.37
AB00477844	SNAI2	1	PY00001012	P012	1328.00	16.71	-10.40
AB00477844	SNAI2	1	PY00001019	P012	653.00	-4.25	-14.82
AB00477844	SNAI2	1	PY00000207	P012	408.00	-73.25	17.59
AB00477845	SNAI2	1	PY00001012	P013	1177.00	10.60	4.90
AB00477845	SNAI2	1	PY00001019	P013	744.00	83.40	-34.17
AB00477845	SNAI2	1	PY00000207	P013	494.00	-32.96	-0.98
AB00477846	SNAI2	1	PY00001012	P014	1220.00	6.93	2.02
AB00477846	SNAI2	1	PY00001019	P014	630.00	-1.68	-14.07
AB00477846	SNAI2	1	PY00000207	P014	582.00	33.06	-23.32
AB00477847	TEF	1	PY00001012	P015	1203.00	17.38	2.30
AB00477847	TEF	1	PY00001019	P015	777.00	24.68	-46.73
AB00477847	TEF	1	PY00000207	P015	541.00	12.92	-22.16
AB00477848	TEF	1	PY00001012	P016	1137.00	37.58	10.87
AB00477848	TEF	1	PY00001019	P016	566.00	19.39	3.52
AB00477848	TEF	1	PY00000207	P016	425.00	-37.44	17.30
AB00477849	TEF	1	PY00001012	P017	916.00	20.34	29.25
AB00477849	TEF	1	PY00001019	P017	739.00	-0.27	-38.19
AB00477849	TEF	1	PY00000207	P017	509.00	5.58	-9.10
AB00477850	TEF	1	PY00001012	P018	1310.00	36.07	-8.19
AB00477850	TEF	1	PY00001019	P018	592.00	23.83	0.00
AB00477850	TEF	1	PY00000207	P018	470.00	9.52	1.34
AB00477851	TF	1	PY00001012	P019	1075.00	7.56	13.47
AB00477851	TF	1	PY00001019	P019	674.00	38.91	-23.87
AB00477851	TF	1	PY00000207	P019	488.00	96.47	-5.62
AB00477852	TF	1	PY00001012	P020	1272.00	6.28	-5.20
AB00477852	TF	1	PY00001019	P020	458.00	-29.17	27.14
AB00477852	TF	1	PY00000207	P020	451.00	33.19	0.76
AB00477853	TF	1	PY00001012	P021	1122.00	-7.30	10.68
AB00477853	TF	1	PY00001019	P021	618.00	7.42	-7.79
AB00477853	TF	1	PY00000207	P021	437.00	-1.52	10.34
AB00477854	TF	1	PY00001012	P022	1346.00	13.55	-6.84
AB00477854	TF	1	PY00001019	P022	421.00	24.18	32.91
AB00477854	TF	1	PY00000207	P022	352.00	-9.53	37.32
AB00477855	TFAP2BL1	2	PY00001004	A003	1124.00	-34.18	10.33
AB00477855	TFAP2BL1	2	PY00001006	A003	742.00	-0.65	-46.09
AB00477855	TFAP2BL1	2	PY00000505	A003	535.00	-53.00	2.79
AB00477856	TFAP2BL1	2	PY00001004	A004	962.00	-31.94	20.77

AB00477856	TFAP2BL1	2	PY00001006	A004	550.00	14.22	4.14
AB00477856	TFAP2BL1	2	PY00000505	A004	504.00	-34.16	7.52
AB00477857	TFAP2BL1	2	PY00001004	A005	902.00	11.64	29.74
AB00477857	TFAP2BL1	2	PY00001006	A005	701.00	-10.57	-30.16
AB00477857	TFAP2BL1	2	PY00000505	A005	546.00	-22.60	-3.94
AB00477858	TFAP2BL1	2	PY00001004	A006	1344.00	-39.15	-10.37
AB00477858	TFAP2BL1	2	PY00001006	A006	674.00	-8.54	-20.44
AB00477858	TFAP2BL1	2	PY00000505	A006	505.00	-67.02	3.29
AB00477859	TGFB2	2	PY00001004	A007	1297.00	20.15	-9.97
AB00477859	TGFB2	2	PY00001006	A007	583.00	10.88	-10.18
AB00477859	TGFB2	2	PY00000505	A007	599.00	-28.77	-12.42
AB00477860	TGFB2	2	PY00001004	A008	960.00	-14.20	16.14
AB00477860	TGFB2	2	PY00001006	A008	700.00	46.21	-40.42
AB00477860	TGFB2	2	PY00000505	A008	489.00	3.76	12.26
AB00477861	TGFB2	2	PY00001004	A009	906.00	7.12	27.87
AB00477861	TGFB2	2	PY00001006	A009	691.00	-14.10	-33.40
AB00477861	TGFB2	2	PY00000505	A009	588.00	-5.32	-3.94
AB00477862	TGFB2	2	PY00001004	A010	1557.00	-10.97	-35.10
AB00477862	TGFB2	2	PY00001006	A010	720.00	-14.41	-36.10
AB00477862	TGFB2	2	PY00000505	A010	433.00	-36.43	29.96
AB00477863	THAP5	2	PY00001004	A011	1319.00	-23.48	-9.18
AB00477863	THAP5	2	PY00001006	A011	668.00	-6.69	-15.85
AB00477863	THAP5	2	PY00000505	A011	532.00	-74.27	3.04
AB00477864	THAP5	2	PY00001004	A012	1162.00	-30.01	4.51
AB00477864	THAP5	2	PY00001006	A012	659.00	30.61	-15.31
AB00477864	THAP5	2	PY00000505	A012	557.00	-24.68	-7.18
AB00477865	THAP5	2	PY00001004	A013	1335.00	-34.79	-11.15
AB00477865	THAP5	2	PY00001006	A013	709.00	43.92	-40.69
AB00477865	THAP5	2	PY00000505	A013	586.00	-73.22	-6.18
AB00477866	THAP5	2	PY00001004	A014	1589.00	12.72	-33.72
AB00477866	THAP5	2	PY00001006	A014	615.00	10.70	-12.34
AB00477866	THAP5	2	PY00000505	A014	524.00	-66.25	3.29
AB00477867	TKTL1	2	PY00001004	A015	1194.00	18.10	3.53
AB00477867	TKTL1	2	PY00001006	A015	533.00	12.90	6.84
AB00477867	TKTL1	2	PY00000505	A015	423.00	-61.14	26.22
AB00477868	TKTL1	2	PY00001004	A016	1162.00	40.39	7.08
AB00477868	TKTL1	2	PY00001006	A016	472.00	34.87	18.99
AB00477868	TKTL1	2	PY00000505	A016	474.00	-55.07	15.75
AB00477869	TKTL1	2	PY00001004	A017	1446.00	42.65	-21.11
AB00477869	TKTL1	2	PY00001006	A017	575.00	55.94	-10.45
AB00477869	TKTL1	2	PY00000505	A017	610.00	-43.53	-7.18
AB00477870	TKTL1	2	PY00001004	A018	1529.00	54.51	-28.10
AB00477870	TKTL1	2	PY00001006	A018	508.00	39.36	3.05
AB00477870	TKTL1	2	PY00000505	A018	523.00	60.50	-3.19
AB00477871	TMEM23	2	PY00001004	A019	1238.00	40.73	-3.17
AB00477871	TMEM23	2	PY00001006	A019	684.00	23.20	-35.83

AB00477871	TMEM23	2	PY00000505	A019	608.00	82.83	-11.67
AB00477872	TMEM23	2	PY00001004	A020	1237.00	47.04	-3.27
AB00477872	TMEM23	2	PY00001006	A020	602.00	26.12	-7.75
AB00477872	TMEM23	2	PY00000505	A020	419.00	24.75	26.47
AB00477873	TMEM23	2	PY00001004	A021	1212.00	1.31	-1.99
AB00477873	TMEM23	2	PY00001006	A021	416.00	41.07	21.42
AB00477873	TMEM23	2	PY00000505	A021	544.00	-47.61	0.05
AB00477874	TMEM23	2	PY00001004	A022	771.00	25.87	37.33
AB00477874	TMEM23	2	PY00001006	A022	473.00	-18.87	19.26
AB00477874	TMEM23	2	PY00000505	A022	428.00	30.43	18.99
AB00477875	TP53	2	PY00001004	B003	1485.00	7.87	-29.78
AB00477875	TP53	2	PY00001006	B003	628.00	29.31	-25.57
AB00477875	TP53	2	PY00000505	B003	495.00	-56.45	14.25
AB00477876	TP53	2	PY00001004	B004	1024.00	58.59	16.24
AB00477876	TP53	2	PY00001006	B004	381.00	72.35	34.65
AB00477876	TP53	2	PY00000505	B004	512.00	-27.20	8.77
AB00477877	TP53	2	PY00001004	B005	1346.00	-1.73	-16.97
AB00477877	TP53	2	PY00001006	B005	566.00	21.49	-11.80
AB00477877	TP53	2	PY00000505	B005	523.00	-41.99	4.53
AB00477878	TP53	2	PY00001004	B006	1210.00	-19.38	-0.02
AB00477878	TP53	2	PY00001006	B006	561.00	-12.54	-11.26
AB00477878	TP53	2	PY00000505	B006	475.00	-52.19	14.75
AB00477879	TPX2	2	PY00001004	B007	1022.00	-28.06	20.18
AB00477879	TPX2	2	PY00001006	B007	467.00	-32.61	23.85
AB00477879	TPX2	2	PY00000505	B007	509.00	-52.77	7.52
AB00477880	TPX2	2	PY00001004	B008	1015.00	39.65	20.77
AB00477880	TPX2	2	PY00001006	B008	382.00	-24.22	35.73
AB00477880	TPX2	2	PY00000505	B008	614.00	10.19	-10.67
AB00477881	TPX2	2	PY00001004	B009	942.00	-48.96	33.29
AB00477881	TPX2	2	PY00001006	B009	398.00	-33.57	38.16
AB00477881	TPX2	2	PY00000505	B009	433.00	1.19	27.22
AB00477882	TPX2	2	PY00001004	B010	699.00	-52.15	53.09
AB00477882	TPX2	2	PY00001006	B010	289.00	-46.35	62.46
AB00477882	TPX2	2	PY00000505	B010	499.00	-8.09	14.01
AB00477883	TRAF3	2	PY00001004	B011	1374.00	19.38	-18.25
AB00477883	TRAF3	2	PY00001006	B011	606.00	-2.23	-16.12
AB00477883	TRAF3	2	PY00000505	B011	525.00	-46.84	2.79
AB00477884	TRAF3	2	PY00001004	B012	1224.00	-25.18	-6.33
AB00477884	TRAF3	2	PY00001006	B012	551.00	-15.81	-3.97
AB00477884	TRAF3	2	PY00000505	B012	501.00	-16.39	16.00
AB00477885	TRAF3	2	PY00001004	B013	1140.00	-10.58	9.15
AB00477885	TRAF3	2	PY00001006	B013	465.00	8.03	18.45
AB00477885	TRAF3	2	PY00000505	B013	532.00	63.89	1.54
AB00477886	TRAF3	2	PY00001004	B014	1327.00	45.72	-17.07
AB00477886	TRAF3	2	PY00001006	B014	520.00	-20.82	8.19
AB00477886	TRAF3	2	PY00000505	B014	641.00	30.29	-17.40

AB00477887	TSC	2	PY00001004	B015	1140.00	-30.90	8.16
AB00477887	TSC	2	PY00001006	B015	410.00	-11.44	27.63
AB00477887	TSC	2	PY00000505	B015	487.00	-31.98	15.75
AB00477888	TSC	2	PY00001004	B016	1195.00	35.87	-3.96
AB00477888	TSC	2	PY00001006	B016	478.00	17.95	13.05
AB00477888	TSC	2	PY00000505	B016	543.00	-17.59	2.79
AB00477889	TSC	2	PY00001004	B017	1145.00	-20.17	7.08
AB00477889	TSC	2	PY00001006	B017	433.00	12.13	20.88
AB00477889	TSC	2	PY00000505	B017	548.00	41.46	-4.94
AB00477890	TSC	2	PY00001004	B018	1336.00	45.23	-10.37
AB00477890	TSC	2	PY00001006	B018	379.00	18.81	26.55
AB00477890	TSC	2	PY00000505	B018	585.00	25.30	0.55
AB00477891	TSC2	2	PY00001004	B019	1444.00	-8.85	-23.57
AB00477891	TSC2	2	PY00001006	B019	536.00	3.40	-3.16
AB00477891	TSC2	2	PY00000505	B019	492.00	0.35	12.26
AB00477892	TSC2	2	PY00001004	B020	1222.00	-17.62	-0.02
AB00477892	TSC2	2	PY00001006	B020	509.00	35.38	-3.16
AB00477892	TSC2	2	PY00000505	B020	507.00	21.97	8.02
AB00477893	TSC2	2	PY00001004	B021	1065.00	66.89	13.88
AB00477893	TSC2	2	PY00001006	B021	297.00	70.98	44.64
AB00477893	TSC2	2	PY00000505	B021	465.00	-30.01	20.24
AB00477894	TSC2	2	PY00001004	B022	841.00	16.57	32.01
AB00477894	TSC2	2	PY00001006	B022	463.00	4.75	8.46
AB00477894	TSC2	2	PY00000505	B022	608.00	51.16	-13.66
AB00477895	UBB	2	PY00001004	C003	979.00	-49.07	29.45
AB00477895	UBB	2	PY00001006	C003	305.00	-57.65	70.03
AB00477895	UBB	2	PY00000505	C003	501.00	-16.72	7.77
AB00477896	UBB	2	PY00001004	C004	1329.00	1.47	-10.96
AB00477896	UBB	2	PY00001006	C004	504.00	-4.89	16.29
AB00477896	UBB	2	PY00000505	C004	421.00	19.44	21.23
AB00477897	UBB	2	PY00001004	C005	535.00	-61.73	72.51
AB00477897	UBB	2	PY00001006	C005	294.00	-46.42	74.89
AB00477897	UBB	2	PY00000505	C005	457.00	-45.80	19.24
AB00477898	UBB	2	PY00001004	C006	1345.00	-2.60	-13.12
AB00477898	UBB	2	PY00001006	C006	519.00	-37.73	8.19
AB00477898	UBB	2	PY00000505	C006	479.00	-0.81	8.02
AB00477899	UBC	2	PY00001004	C007	668.00	-52.68	55.76
AB00477899	UBC	2	PY00001006	C007	358.00	-43.00	57.33
AB00477899	UBC	2	PY00000505	C007	477.00	-43.59	12.51
AB00477900	UBC	2	PY00001004	C008	919.00	-48.73	36.34
AB00477900	UBC	2	PY00001006	C008	497.00	-39.48	29.79
AB00477900	UBC	2	PY00000505	C008	500.00	-24.40	8.02
AB00477901	UBC	2	PY00001004	C009	1104.00	-23.96	12.10
AB00477901	UBC	2	PY00001006	C009	589.00	-5.85	-6.94
AB00477901	UBC	2	PY00000505	C009	527.00	23.91	2.04
AB00477902	UBC	2	PY00001004	C010	360.00	-50.71	83.54

AB00477902	UBC	2	PY00001006	C010	285.00	-24.67	90.55
AB00477902	UBC	2	PY00000505	C010	287.00	-97.13	55.13
AB00477903	UNQ739	2	PY00001004	C011	1454.00	10.26	-25.24
AB00477903	UNQ739	2	PY00001006	C011	473.00	-12.67	12.24
AB00477903	UNQ739	2	PY00000505	C011	472.00	10.70	15.00
AB00477904	UNQ739	2	PY00001004	C012	986.00	-5.40	24.03
AB00477904	UNQ739	2	PY00001006	C012	497.00	3.89	21.96
AB00477904	UNQ739	2	PY00000505	C012	444.00	-19.93	24.22
AB00477905	UNQ739	2	PY00001004	C013	977.00	-1.23	27.77
AB00477905	UNQ739	2	PY00001006	C013	483.00	42.53	17.10
AB00477905	UNQ739	2	PY00000505	C013	564.00	-8.87	0.79
AB00477906	UNQ739	2	PY00001004	C014	1296.00	-8.97	-7.41
AB00477906	UNQ739	2	PY00001006	C014	468.00	5.45	14.13
AB00477906	UNQ739	2	PY00000505	C014	488.00	2.44	8.02
AB00477907	WEE1	2	PY00001004	C015	655.00	-63.24	59.50
AB00477907	WEE1	2	PY00001006	C015	322.00	-43.13	61.38
AB00477907	WEE1	2	PY00000505	C015	397.00	-26.11	36.44
AB00477908	WEE1	2	PY00001004	C016	1531.00	-22.41	-31.26
AB00477908	WEE1	2	PY00001006	C016	464.00	16.76	20.61
AB00477908	WEE1	2	PY00000505	C016	424.00	-103.80	25.22
AB00477909	WEE1	2	PY00001004	C017	765.00	-44.11	44.92
AB00477909	WEE1	2	PY00001006	C017	221.00	-0.27	68.14
AB00477909	WEE1	2	PY00000505	C017	518.00	2.09	5.03
AB00477910	WEE1	2	PY00001004	C018	1444.00	4.82	-22.49
AB00477910	WEE1	2	PY00001006	C018	492.00	-15.76	23.31
AB00477910	WEE1	2	PY00000505	C018	381.00	-28.77	32.45
AB00477911	WFDC11	2	PY00001004	C019	1165.00	-39.01	6.09
AB00477911	WFDC11	2	PY00001006	C019	408.00	-16.35	32.22
AB00477911	WFDC11	2	PY00000505	C019	492.00	-53.33	8.27
AB00477912	WFDC11	2	PY00001004	C020	1463.00	14.80	-25.05
AB00477912	WFDC11	2	PY00001006	C020	386.00	13.15	26.82
AB00477912	WFDC11	2	PY00000505	C020	430.00	-27.32	24.47
AB00477913	WFDC11	2	PY00001004	C021	1281.00	5.40	-6.23
AB00477913	WFDC11	2	PY00001006	C021	590.00	15.17	-11.53
AB00477913	WFDC11	2	PY00000505	C021	499.00	-7.27	2.54
AB00477914	WFDC11	2	PY00001004	C022	1304.00	24.22	-6.33
AB00477914	WFDC11	2	PY00001006	C022	531.00	14.23	11.43
AB00477914	WFDC11	2	PY00000505	C022	428.00	37.14	18.74
AB00477915	WNT9B	2	PY00001004	D003	1298.00	-22.63	-9.08
AB00477915	WNT9B	2	PY00001006	D003	470.00	-24.16	5.49
AB00477915	WNT9B	2	PY00000505	D003	490.00	-48.28	15.00
AB00477916	WNT9B	2	PY00001004	D004	1347.00	-24.47	-14.70
AB00477916	WNT9B	2	PY00001006	D004	550.00	3.08	-5.05
AB00477916	WNT9B	2	PY00000505	D004	601.00	-14.74	-10.17
AB00477917	WNT9B	2	PY00001004	D005	1059.00	-23.30	18.80
AB00477917	WNT9B	2	PY00001006	D005	401.00	2.67	29.79

AB00477917	WNT9B	2	PY00000505	D005	579.00	-25.34	-6.68
AB00477918	WNT9B	2	PY00001004	D006	1384.00	2.94	-17.66
AB00477918	WNT9B	2	PY00001006	D006	481.00	-26.91	16.29
AB00477918	WNT9B	2	PY00000505	D006	517.00	7.18	1.29
AB00477919	YAP	2	PY00001004	D007	1253.00	51.60	-6.52
AB00477919	YAP	2	PY00001006	D007	473.00	-7.95	15.21
AB00477919	YAP	2	PY00000505	D007	474.00	-27.64	20.49
AB00477920	YAP	2	PY00001004	D008	1040.00	10.36	12.30
AB00477920	YAP	2	PY00001006	D008	482.00	12.15	14.67
AB00477920	YAP	2	PY00000505	D008	550.00	51.24	2.29
AB00477921	YAP	2	PY00001004	D009	1180.00	3.14	-0.61
AB00477921	YAP	2	PY00001006	D009	457.00	2.65	16.29
AB00477921	YAP	2	PY00000505	D009	436.00	-29.88	22.73
AB00477922	YAP	2	PY00001004	D010	1045.00	-33.29	15.16
AB00477922	YAP	2	PY00001006	D010	321.00	-26.47	50.58
AB00477922	YAP	2	PY00000505	D010	555.00	-8.80	-6.43
AB00477923	ZDHHC18	2	PY00001004	D011	991.00	-19.86	20.48
AB00477923	ZDHHC18	2	PY00001006	D011	544.00	7.57	-7.21
AB00477923	ZDHHC18	2	PY00000505	D011	522.00	-46.69	4.53
AB00477924	ZDHHC18	2	PY00001004	D012	699.00	-59.72	50.24
AB00477924	ZDHHC18	2	PY00001006	D012	298.00	-29.81	60.84
AB00477924	ZDHHC18	2	PY00000505	D012	490.00	-51.84	11.01
AB00477925	ZDHHC18	2	PY00001004	D013	827.00	-48.61	41.27
AB00477925	ZDHHC18	2	PY00001006	D013	483.00	-10.54	10.35
AB00477925	ZDHHC18	2	PY00000505	D013	441.00	8.60	18.74
AB00477926	ZDHHC18	2	PY00001004	D014	817.00	1.00	35.85
AB00477926	ZDHHC18	2	PY00001006	D014	530.00	35.38	1.16
AB00477926	ZDHHC18	2	PY00000505	D014	473.00	-12.06	15.50
AB00477927	ZNF322A	2	PY00001004	D015	1230.00	-17.34	-4.26
AB00477927	ZNF322A	2	PY00001006	D015	310.00	-10.84	41.67
AB00477927	ZNF322A	2	PY00000505	D015	448.00	42.72	21.73
AB00477928	ZNF322A	2	PY00001004	D016	1150.00	-35.62	5.70
AB00477928	ZNF322A	2	PY00001006	D016	511.00	-16.09	5.22
AB00477928	ZNF322A	2	PY00000505	D016	609.00	-4.12	-17.40
AB00477929	ZNF322A	2	PY00001004	D017	1117.00	-26.26	12.00
AB00477929	ZNF322A	2	PY00001006	D017	484.00	-19.69	14.67
AB00477929	ZNF322A	2	PY00000505	D017	496.00	30.57	4.03
AB00477930	ZNF322A	2	PY00001004	D018	1195.00	-24.15	0.18
AB00477930	ZNF322A	2	PY00001006	D018	372.00	-21.44	40.86
AB00477930	ZNF322A	2	PY00000505	D018	485.00	-14.89	12.76
AB00477931	ZNF513	2	PY00001004	D019	1063.00	45.09	12.50
AB00477931	ZNF513	2	PY00001006	D019	285.00	89.69	41.94
AB00477931	ZNF513	2	PY00000505	D019	436.00	87.56	21.98
AB00477932	ZNF513	2	PY00001004	D020	993.00	-39.22	26.00
AB00477932	ZNF513	2	PY00001006	D020	291.00	42.24	46.53
AB00477932	ZNF513	2	PY00000505	D020	535.00	21.50	4.53

AB00477933	ZNF513	2	PY00001004	D021	931.00	-38.71	31.02
AB00477933	ZNF513	2	PY00001006	D021	274.00	-28.09	54.90
AB00477933	ZNF513	2	PY00000505	D021	476.00	-44.92	9.52
AB00477534	ZNF513	2	PY00001004	D022	1182.00	-17.44	-1.40
AB00477534	ZNF513	2	PY00001006	D022	637.00	22.96	-29.08
AB00477534	ZNF513	2	PY00000505	D022	528.00	18.05	1.79

Table S2. scRNA-seq sample information.

Name	Number of cells (post-QC)	Number of cells (>10k reads)	Mean reads per cell	Total number of reads	Median genes per cell	Total genes	Mean UMI per cell	Median UMI per cell	Total UMIs
GS5A	316	471	66707	31418884	2816	20540	20174	7989	9501998
GS5B	864	1310	27963	36603973	1811	23500	7414	5258	9711821
GS5E	1389	1824	26661	48656029	2729	21437	11952	9207	21812302
GS5G	843	98718110	18869	18642521	3018	20023	10149	7956	10027084

Table S3. Candidates identified by scRNA-seq.

Astrocyte (n=1469)

MALAT1	NADK2	TRPM7	ZNF106	MSH3	RGS17
MEG3	REL	TRAPPC3	UCHL5	ATR	CCZ1B
NEAT1	SGOL2	LTN1	KPNA1	GMCL1	ANGEL2
WSB1	LINC00969	ARL5B	NUPL2	ARIH2	STAG1
ATF5	APAF1	C7orf41	KLHL7	DCLK2	ZNF512
TRIM9	AEN	DHX15	FERMT2	ZNF431	DCAF10
GOLGA4	DCAF7	GPATCH2L	DCUN1D4	COQ9	ZNF721
CALD1	SNRNP70	TNRC6A	AP3M2	ERCC4	SFPQ
PLAT	ONECUT2	EXTL3	NASP	DDR2	FRMD4A
PLEKHA4	C6orf62	U2AF1	CLASP1	CBS	EIF3B
CCNL1	HPS4	PPP2R3C	SLC35E1	CNTLN	DDX6
EPHA3	LUC7L	PRR11	CRYZL1	DLX6-AS1	NUP214
RBM25	SLC38A2	ZNF146	ZBTB43	CCDC174	EFNB2
MDM2	AGPAT5	NOL11	PXDN	KIAA0020	SMCHD1
TOP2A	BICD1	C3orf58	SF3B1	TRIM33	ARFGEF1
NKTR	GPS1	EIF5B	RBM14	ARID4A	ZBTB21
VEGFA	RHOJ	PPP1R2	CTNND1	PRKAB1	LAS1L
XPO1	COBL	GTF3C3	NAA25	TMEM170A	IQCG
PTPRZ1	TAF1	AFTPH	ZBTB25	FGF13	UNK
KMT2E	XPC	FTSJ3	GPRC5B	RP11-166D19.1	NAGK
UBE2C	CEP152	DDX46	DBF4	SP140L	DIAPH2
MLC1	MKNK2	CREBZF	PSMC6	VPS13A	PARP8
ARHGEF6	RIOK3	KIF20B	ANKZF1	GTPBP10	PTPN13
PCLO	NUFIP2	C10orf137	ACAP2	ACVR2B	KPNB1
COA1	IFI16	TRIM22	USP9Y	SMAD5	KIAA0947
HSPA1A	STK32A	PIAS2	CHD6	CDC42EP3	STX10
FNBP4	CELF1	ARID4B	UPF3A	USP9X	USP7
CLK1	SPECC1	FAR1	DYNC2LI1	MALSU1	KDM4A
DDX3Y	KHSRP	WDR52	AKAP17A	SGSM1	JPX
RBM39	RP11-473I1.10	FBXO11	ORC5	PAG1	CNOT4
HSPA1B	RNMT	PEX2	INSR	SPG20	KANK1
ASNS	ASIC4	PSMC1	SIPA1L2	SSU72	WDR43
ZMYND8	MARK3	CD47	QSER1	PRDM2	ATG14
BOD1L1	NFIA	UBR3	BRCC3	METTL2B	TBC1D5
KDM3A	ZCCHC11	PCNX	SNAP29	TMEM30A	RBM22
ZC3H13	RLIM	GTF2A1	CBLL1	DZIP3	TMEM41B
TRA2A	LARP7	PTCD3	TNPO1	CDC37	APBB2
BBC3	SSBP2	RNF19A	MLLT6	TNRC6C	POLI
MAP2	RAB6A	FAM118A	NFATC4	DNPEP	ADAR
PER1	ZNF680	SNX13	SF1	PARP14	SMARCA5
CREBRF	SLC7A11	MPHOSPH10	ZEB2	NAA38	NPAS2
PDK1	ITSN1	DKC1	SERTAD2	OSGEP	SART1

CDK1	CTNNB1	CMPK1	AC005154.6	VEZF1	PTPRS
PNN	FGFR1OP2	PRRC2C	OIP5-AS1	HOXB4	C17orf85
DHX36	ABI2	DYNC2H1	NUB1	BAIAP3	CEP78
SLC25A36	ANKRA2	ANKRD10	DNAJC9	TLK1	LARP4
XRN2	FAM133B	CDK5RAP2	COPB2	PRPF39	MINA
HES7	ATP13A3	TM2D1	MTHFD1	RP11-315A16.1	PHF14
RBPJ	NPEPPS	SLC11A2	RUFY3	PCDHB16	MAP4
LUC7L3	LYPLAL1	TMEM165	ZNF439	PRPS1	KNOP1
YTHDC1	MNS1	GCFC2	ICA1	MYO6	JAM3
SPATA22	TMEM259	NSUN2	RASA2	DIP2B	SAPCD2
PBK	BCL6	USP16	FAM179B	LARP1	LSG1
CENPF	AKAP9	DDX55	FOXP1	CCNL2	RCOR1
RAB34	C2orf69	PCSK7	PPIP5K2	ACIN1	ETNK1
BDP1	PPM1A	NCKAP1	TTC3	KIF13B	TRPS1
PNISR	RBMX	KIAA1033	RAD50	NAA15	NCOR1
ZNF207	TTC14	KDM2A	CDK5RAP3	MGME1	CTBP1
RSRC2	AKAP8L	LINC00662	C5orf24	GPALPP1	STXBP1
ANKRD36C	THAP9-AS1	DAZAP1	MLH1	NOL10	RB1CC1
TFG	DDB2	THADA	C10orf118	XRCC2	FAT1
ZNF91	TRIM4	N4BP2L2	NUP107	UACA	BRD8
H2AFY	ELAVL3	ZNF667	BRE	SLC5A3	SHPRH
MGEA5	SS18L1	SOS1	PKD1	PCMTD2	SP100
FBXW7	SMC2	POLDIP3	ZNF273	TNRC6B	YTHDF3
MSMO1	ZNF83	MAFG	TBRG1	ZNF506	FENDRR
PPP1R3C	KLHL28	RYK	SLTM	MTMR9	GOLGA3
PRKDC	LMO2	GLS	WNK3	PCM1	TXLNG
CWF19L2	DST	SETD3	CPSF3	SLC25A12	EXOC7
HBP1	PPIG	DDX23	ERICH1	AGO3	PIGL
EPN2	NAP1L4	TSPYL2	CABIN1	CLDN12	WDR6
IARS	MDM4	PRUNE2	ZC3H15	ILKAP	TSHZ2
CCNB1	GOLT1B	HEXIM1	SEL1L3	SLC23A2	CBX6
ABCF1	SPAG9	TMEM50B	POLR2B	FBXO3	MPLKIP
TMF1	BRWD1	STARD4	C3orf17	WDR33	TMEM237
QKI	FZD3	ARFGAP1	IMP4	PHLDA1	ELFN1
GPCPD1	MMP16	NFX1	REEP2	PMM2	FAM50A
ZNF395	ZNF121	PRMT2	TIA1	DCAF16	PKN2
PDHA1	PABPN1	PRDM4	EVI5	RNF10	STIM2
NUPL1	DIP2A	ARL5A	SCAF4	CEP44	PRELID2
HSP90AA1	MYEF2	PDCD4	DENND4C	PLXNB2	SAP30BP
ANKRD36	YARS	HMGN1	ZBTB44	RAD54L2	DCP2
HNRNPM	RNF20	LDLR	ZNF12	DENR	PAFAH1B1
PCDH17	ELP2	NR1D2	TRIM44	CEP97	PUS7L
GOLGB1	RANBP2	AKAP11	CNDP2	SMC5	KCTD7
SMC4	MORC3	ZC3HAV1	ZNF800	TTC9C	SPRED1

ZSCAN18	SPAG5	TEAD1	ATP5S	PHF6	CEP350
CKAP2	CHERP	CIAO1	NOL3	GOSR1	SEPT6
MAR6	PRPF6	BNIP2	PHIP	MORC4	CEP112
GGH	HMGCS1	TCERG1	HDAC3	EP300	ZNF326
TRA2B	FNIP1	ATRX	PCNXL4	NAA16	MUT
MKI67	SRPK1	RCOR3	WDFY3	TIAL1	IQSEC1
FDFT1	GBE1	CAMK2D	RAP2C	NADSYN1	OSBPL6
ARGLU1	YAE1D1	THOC2	MED13	WAC	EWSR1
IFRD1	PURB	RBM12B	MYCBP2	CEP70	KIAA1429
XRN1	CWC25	GRIP2	ZNF445	SECISBP2	PISD
HSPA4L	WBP4	CCDC88A	ERV3-1	CCNH	ANKS3
EXOSC8	ROCK2	TRIP11	ACBD3	RBAK	BMS1
NOP56	SAFB	FYN	ZBTB11	ZNF197	CDC42SE2
RP11-1114A5.4	DERL1	SRSF5	TRPC4AP	TRAPPC6B	ALDH7A1
ZNF844	DR1	ACYP2	GNB5	NRDE2	SMC1A
RBM6	CDC27	TM9SF2	ASH1L	SPDL1	ABCE1
BTAF1	TSC1	ARHGAP12	AFAP1L1	ZRANB1	NUP85
ARL6IP6	BAZ2A	LZTFL1	CSTF3	TTC19	MED6
LENG8	TET1	SLC35F5	ZFYVE20	CAMSAP2	RFC1
PFKFB4	SUPT5H	PER2	ZFHX4	POLR2H	SETX
KLHL24	FAM111A	C7orf55-LUC7L2	NRD1	BAZ1A	KIAA1731
LSAMP	PUM2	ADD3	GAPVD1	RSBN1L	UBE3C
BIRC5	JMJD6	IVD	SESN1	HEXA	SNHG10
PAXBP1	ECT2	DNAJC30	MBOAT2	WHAMM	GNL3
ASRGL1	CCP110	CEP57	SLC4A7	NDEL1	PHF20L1
UTRN	FUT11	AARS	ST3GAL5	HAP1	MPHOSPH6
ZNF292	MAT2A	UBA5	SCD	MAP4K5	UBA3
PCDH9	RBM38	NNT-AS1	CCDC53	EHMT2	CNTRL
FDXR	TANC1	GFM1	ANKRD17	ARMCX3	MAP2K7
AIMP2	ZBTB10	SPTY2D1	PHF21B	COL6A1	SRP19
SON	C12orf10	NEMF	TOP2B	CAPN7	GUF1
NOP58	ZNF638	MIF4GD	ATP11B	ZNF528	PPP6R3
RGS6	STX16	NSRP1	BPTF	SATB1	DCAF11
SETD5	REV3L	PRPF38A	UBN1	DDX39A	OXSR1
TLK2	PHF17	CEP290	SUGT1	TMEM87A	CTTN
THY1	ASAP1	CBFA2T2	TRIM24	RAI14	RUFY2
NUF2	PTK2	KAT2A	RNF169	SPATS2	RARS
TPX2	EPM2AIP1	PCF11	LONP1	CHD9	SOX6
ITSN2	SUN1	GPR155	PPP1R12A	ZBTB7A	AP3B1
CBR1	SLC44A1	ATP2A2	ABHD13	AKAP12	DDX56
CPEB2	NUP54	WAC-AS1	SFSWAP	DNAJC13	ADAM10
FAM122B	ORC4	PRKAB2	C11orf58	TNPO2	SLMAP
POLE4	HLTF	ARHGAP21	RQCD1	PRKRIP1	UVRAG

CTC-444N24.11	TRPM4	UPF2	ADNP	TNKS	OFD1
PREPL	AKAP10	PPA2	FNDC3A	ATPAF1	ZNF33A
KIAA0907	CCNC	SENP7	ZNF529	FAM102A	MLTK
HSPH1	SRPR	HIF1A	CDS2	YAP1	DTX3
ANKRD12	SRP54	ZC3H11A	MTERFD2	ZNF138	CHRNB1
U2SURP	ZC3H7A	KBTBD2	LAMC1	TMEM43	KDSR
SULT1C2	HIST1H1D	SRSF6	CTD-3018O17.3	RC3H2	GSPT2
CEP95	CIB1	MON2	DNAJC25	RSAD1	EPB41L5
ARHGAP5	ZFYVE16	DDX42	SETD2	FAM161A	N4BP2
SNX5	GDI1	MAR7	BAALC	BTBD7	C17orf75
ACOT13	GPBP1	FANCI	FRA10AC1	PARN	CCDC43
ZFC3H1	MAP3K2	SOX2	ILF3-AS1	GATAD1	HMGXB4
GRIA2	INSIG1	BAG5	WDR61	KAT6A	RNF213
MICU2	CRY2	GPX8	RNF145	ASAP2	PIK3C3
ATAD2	MKLN1	TTC17	GIGYF2	ASB8	EHMT1
FLCN	PTBP3	ARMCX4	AGFG1	NLGN1	ZCCHC6
SLC4A4	BBX	CDC42BPA	CTC-228N24.3	DNAJB6	MYH10
SRSF1	NDUFAF4	DHX57	SP4	MPP6	PRPF8
IGF2BP3	PRPF38B	CHD1	PANK3	SAMD4A	ZC3H8
RIF1	ZNF44	CLSPN	C2orf49	TSC22D2	TRNT1
SMEK1	ASCC3	IWS1	NAA50	GYS1	KIF16B
SQLE	SMARCA4	POU3F2	CDK12	UTY	PTPN12
SUCLA2	ZC2HC1A	SENP2	KTN1	KATNBL1	PRRC2B
ZNF791	PPP1CC	TCF25	CCDC66	GOLGA2	NPAS3
SAFB2	MAP4K4	IPO7	TMEM245	RPGR	FAM208A
KIF15	SLFN5	MTF2	TPD52L2	MAP3K1	CHD7
RIMKLB	PMPCB	LYST	UBE2F	NELFCD	IFT81
RSF1	HNRNPD	BRD1	ZC3H14	KIF1B	RPAP3
HIBADH	UBR5	KIDINS220	KIF1A	DNAJC10	HIBCH
SLC20A1	WDR60	FUBP1	EXT2	DNM1L	UTP18
LIG1	POU2F1	CBX5	SMG6	TBC1D23	CACNG8
TRAF4	TOX4	SLC38A1	UPF3B	AC005152.2	C20orf194
PPP2CB	ABCA1	UBXN4	RABGAP1	APOL6	ATXN2
MAPK1IP1L	ZNF518A	NCOR2	AKT1	SFXN3	SPOP
MCL1	SREK1	C1orf63	SMURF2	RNF168	VPS53
RBM33	RBM28	SUPT6H	SNRNP200	RAD18	ANAPC5
TNRC18	PFKP	KIAA0430	NCAPH2	MAP7D2	MYO9A
RBM5	PSMC3IP	SYNE1	CEP68	CTDSPL2	FOXRED1
IRF2BP2	PNP	ACOX1	SOX11	PIP5K1A	ANKIB1
HNRNPA2B1	FBXO7	PDGFRA	EPRS	FMNL2	SKIV2L2
ZDBF2	CTB-89H12.4	PARP9	SEC22A	GINS2	NBAS
YIPF4	GAD1	RP11-220I1.1	MEF2A	CLN5	USP48
NOVA1	CSNK1D	CDK14	RNF150	STX2	NHLRC3

RRM2B	EZH2	PHTF2	MICU1	AKT2	TIMM44
PRC1	NUDT16	KIAA1109	UIMC1	RP11-161M6.2	VPS26A
CHD2	NISCH	GTF2I	PTAR1	ZNF829	PLEKHA8
NUMA1	UHMK1	HNRNPH1	FAM136A	CCDC18	PCNT
USP36	SRGAP3	SNX27	VPS36	CHD3	FKBP15
CCNT2	PDCD6IP	PGPEP1	GNPTAB	TIMELESS	CCDC173
ZMAT1	NPHP3	ANKRD28	BRD2	TRIP12	DIDO1
MORF4L2	HELLS	HMX1	UBXN2A	KIAA2026	USP8
POLR2A	ZNF441	SBF2	RNF24	NFRKB	CSPP1
MSL1	EGR3	LARS	IPO9	MTR	LONRF2
SLC2A3	CHORDC1	MPHOSPH9	PARP2	IMPDH1	KDM5C
CCDC14	MED13L	SLC30A9	DPY19L4	PCGF3	TGS1
CPNE1	CCDC136	DIS3L2	CRAMP1L	SRRT	MBNL1
ALDH3A2	POGZ	NCBP2	TRIM52	SUPT7L	CDC25B
PSRC1	ZMYM2	EVL	ABCD4	CUL4A	ARHGEF12
TM7SF3	CENPC	PRKRA	C11orf54	CPSF6	BCAT2
PLEKHH2	COMMD4	SENP5	NT5DC2	THOC1	FAXDC2
ZNF654	COBLL1	OPA1	TFRC	WNK1	CWC27
ZNF160	METTL12	PCID2	TBC1D14	KLHL42	SENP6
KMT2C	CLK4	ITGA7	XPNPEP1	MGRN1	ZNF608
DNMT1	ZNF563	ANKRD13A	EP400	CENPJ	RGS12
MXD1	FAM126B	SBNO1	MEIS1	PRPF3	LRP6
FAM181B	DDX17	SUDS3	PBRM1	DOCK7	TRAF3IP1
CLASP2	MSI2	PABPC4	SNHG14	RANBP6	DBNL
FAHD1	UTP6	FAM63B	WDR70	VEZT	PHF20
OGT	KDM4B	COL27A1	DIAPH3	DCP1A	R3HDM2
HP1BP3	MKRN2	FOXJ3	CEP250	RETSAT	SRFBP1
LRRC58	CASC3	FAM192A	HMGCR	EXOSC9	VPS8
RLF	ZBTB20	MCM3AP	ZNHIT6	TUG1	MICA
NUP160	UBN2	BRCA1	RBM12	ATM	ZHX1
PHC3	USP37	WHSC1	LRIF1	PAAF1	WAPAL
FAM13A	DNAJC7	STAT3	DIXDC1	NSF	TPM1
COL9A3	SSFA2	USP10	POM121C	PYGB	DAAM1
MIS18BP1	UFM1	ADAMTS9	ZC3H6	ARHGEF26	HPS1
NOM1	RSRC1	C12orf75	FN3KRP	WDR48	C11orf30
ELMSAN1	EXTL2	COPA	TGIF2	MAP1A	FBXO9
SNF8	SRSF11	UBE2G2	GPATCH1	LCORL	AKAP1
LRP1	SEC31A	DZIP1	SLC22A17	SEC16A	TAF2
KMT2A	ZKSCAN1	ESCO1	ZNF738	SMARCAD1	BTN2A1
SYNE2	YEATS2	EEA1	USP33	GON4L	PAK3
GAS7	NUDT3	FNDC3B	ELP6	FGFR1	SUPT20H
RORA	UBQLN1	DSCAM	THUMPD1	RP11-299J3.8	PPFIA1
ADAM9	ZZEF1	CSGALNACT2	CAND1	ZNF711	CCDC50
GCC2	USP1	LPP	RAB3GAP1	NARF	OSBPL1A

BAZ2B	SRRM2	LINC00963	ADNP2	PIBF1	PDS5B
BAZ1B	NRP2	ANXA4	ARHGAP35	PHF3	NRCAM
MIR24-2	CDK13	CTB-31O20.2	CCDC84	COX19	DDX51
PAM	HNRNPA3	CUL3	COPB1	BIRC2	INO80D
BIRC6	DICER1	SEC61A1	KBTBD6	UXS1	ZNFX1
ZNF451	PSME4	FGF11	PGM3	ZNF511	MAP4K3
KANSL1	CKAP5	EIF4G2	MAR5	LPIN1	TPR
PSIP1	BRAF	DLG1	MCM3	PRPF40A	KIAA1958
CXCL3	CCDC93	WDR11	GLRX3	SSBP4	TAF11
RBBP6	METTL14	PRPF4B	PIK3CA	IGF2R	ANKRD26
DMTF1	TAF15	IKBKAP	DEK	LBR	PANK2
CEBPZ	USP15	PCSK5	EED	PDCD11	LRRFIP2
CREB5	HELZ	CCDC34	AASS	TUBGCP3	NAA35
CDC5L	INTU	FRG1B	IQGAP1	EIF2S3	C5orf42
CENPE	CCNT1	SCFD1	STT3B	USP40	FKBP10
LRPPRC	ATP2C1	PTPN1	YIPF6	SUGP2	ISY1
TMPO	MCM4	TMEM116	SETD5-AS1	ATXN1	FNBP1L
SIPA1L1	EPS8	FBXO22	MAP9	CDC16	KAT6B
HNRNPL	LMNA	THUMPD2	SLC25A26	USP14	PRKAG1
ATF7IP	INTS10	PGAP1	RP1-155D22.2	CASC7	DYNC1LI1
ENSA	CLIP4	DDX3X	SPTAN1	TLE4	NAPG
REV1	RAF1	DHX29	FAM76B	FAM13B	AGAP1
ATP2B1	CEP135	TP53BP1	TMCC1	GLYR1	EIF4E
MPHOSPH8	VPS13C	UBAP2	TMEM131	CEP128	FAM219A
CDCA5	ACSL3	TRIM37	BRWD3	PROX1	HOTAIRM1
SLC1A3	GFPT1	WDR26	FRYL	SLC4A10	CCAR1
LL22NC03-2H8.5	RP11-199F11.2	KDM5A	ZNF766	DHX9	AKAP8
DDX18	DNAJB4	USPL1	NOC3L	DFFA	UTP14C
TJP1	FOXK1	SLC33A1	PHF21A	DSCR3	

**Intersect with RNAi screen (n=9)**
PDK1
CCNB1
TPX2
KIF15
CTNNB1
MAP3K2
HIF1A
AKT1
SATB1

Neuron (n=1355)

EGR1	FAM60A	XBP1	NOLC1	CIRBP	PICALM

FAM162A	RAD51AP1	PMAIP1	ZNF711	ZNF844	B4GALT4
FOS	COPA	C19orf60	NARF	CHCHD2	TOPBP1
MALAT1	ZNF480	RP11-473I1.10	PHF3	RBM6	TM2D3
MEG3	UBE2G2	RNMT	COX19	PNRC1	TFRC
NEAT1	ESCO1	MARK3	BIRC2	BTAF1	YTHDC2
PHPT1	FNDC3B	ZCCHC11	AIMP1	BMP7	NEU1
PPIA	TMEM218	PPDPF	TOMM70A	PCNP	PBRM1
WSB1	CSGALNACT2	BANF1	SSBP4	HPRT1	WDR70
ZFAS1	RCHY1	SSBP2	RECK	NDUFS7	DIAPH3
EEF1A1	APOPT1	RAB6A	GLRX5	KLF6	UBALD2
GDF15	SEC24B	ZNF680	BRI3	NDUFB7	ANKLE2
HMGB2	TNFRSF10B	RP11-553L6.5	TRIM23	DDIT4	LETMD1
ATF5	BRCA2	LRRN3	LBR	PAXBP1	ZNHIT6
DDIT3	ANAPC16	TPST1	RP9	UTRN	POM121C
CDKN1A	COPS2	CTNNB1	TUBGCP3	LAMTOR2	UNC119
GLTSCR2	RAP2B	FKBP4	SUGP2	NAMPT	ZC3H6
FKBP9	SNX10	FGFR1OP2	CFDP1	ZNF292	FRG1
EIF4A2	CUL3	SLU7	CASC7	ALKBH5	DCUN1D1
NRN1	SESN3	FAM133B	MBTD1	FDXR	CAND1
PPP1R15A	HNRNPH3	C17orf58	SYNRG	XPOT	ARHGEF9
FAU	NDUFA13	ATP13A3	SLC4A10	SON	RAB3GAP1
GOLGA4	SEC61A1	AES	DHX9	HIGD1A	MAGOH
H3F3B	EIF3G	MZT2B	TOB2	CNBP	PGM3
ENO2	DLG1	NPEPPS	FIP1L1	TIMM13	TGOLN2
IER2	HNRNPDL	MNS1	STAG1	NOP58	EED
FAM211A	PRPF4B	NDUFC1	GABARAPL1	CCNB1IP1	SETD5-AS1
EEF2	FOXN3	JUN	GPR107	SETD5	SLC25A26
FTH1	TCEAL8	EAPP	NEDD4L	UBL5	SPTAN1
CDK6	KDELC2	AKAP9	SFPQ	TLK2	ZCCHC7
SNRPD2	FRG1B	C2orf69	ZFP91	DLX1	SPTLC1
C17orf76-AS1	SCFD1	RBMX	EIF3B	EIF1B	TMEM131
UBA52	COQ4	TTC14	DDX6	SNHG16	SF3B14
EIF4B	PDS5A	TRMT112	EFNB2	HES1	PHF21A
JUND	DDX3X	TCP1	SMCHD1	ITSN2	ATR
FTL	TP53BP1	DDB2	ARFGEF1	USMG5	CALCOCO2
EIF3E	UBAP2	TRIM4	LAS1L	FAM122B	GMCL1
CCNL1	DNAJB2	ELAVL3	CTSL	WDR45B	ARIH2
ODC1	KDM5A	C11orf96	LARP6	CTC-444N24.11	DDR2
ATF4	USPL1	SS18L1	DIAPH2	HEY1	UQCRH
RBM25	SLC33A1	CD81	PTPN13	RP11-71N10.1	CNTLN
EPB41L4A-AS1	KPNA1	KLHL28	KIAA0947	COX7C	CCDC174
NDUFC2	STMN4	C16orf13	USP7	PIM3	KIAA0020
MDM2	APPL1	DRAXIN	COPRS	PREPL	TRIM33

EMC10	VKORC1	UBXN7	CNOT4	SERTAD1	SAV1
TOP2A	ZFAND2A	DST	WDR43	TOMM7	ARID4A
LGALS1	DCUN1D4	MOB4	THRA	ATP5G2	SP140L
DNAJB1	CCDC58	PPIG	SLC6A8	ARRDC4	VPS13A
NKTR	PTRH2	IFI27L2	ADAR	TMEM123	CWC22
ATF3	THSD7A	SPAG9	RGS10	HSPH1	LRR1
VEGFA	NASP	CRBN	PRKCI	ANKRD12	SOX8
H1F0	SLC35E1	EIF4H	C17orf85	TMEM160	CDC42EP3
INSIG2	ZBTB43	BRWD1	GINS4	U2SURP	USP9X
SEC61G	NFYB	DLX2	CEP78	GADD45GIP1	HNRNPLL
SNHG1	HJURP	MMP16	SNAPC3	NPC2	EIF4A3
CA9	SF3B1	RAD23B	SIVA1	C21orf59	PLD3
XPO1	CTNND1	MYEF2	BTBD6	ARHGAP5	CAMK1D
CCNG2	NAA25	DNASE2	WASF2	EIF3L	CDC37
KMT2E	GPRC5B	UBE2D3	LARP4	RRAGD	SF3A1
ZMAT3	WSB2	RANBP2	DDX41	ZFC3H1	ACN9
SLC3A2	ACAP2	SPAG5	EPC2	RP11-798M19.6	DNPEP
RPLP2	MCM2	PRPF6	LINC00116	MINOS1	PARP14
COX6A1	USP9Y	FNIP1	PHF14	NFE2L2	OSGEP
PCLO	CHD6	GBE1	OPTN	ATAD2	C16orf91
PLOD2	DYNC2LI1	MAF	MAP4	SAT1	TLK1
GNB2L1	KPNA4	TMEM14B	TMEM18	SLC4A4	PRPF39
SHFM1	SOS2	GID8	SAPCD2	SRSF1	BFAR
CEBPB	SIPA1L2	PURB	LSG1	RIF1	PCDHB16
HIST1H4C	RNF181	CWC25	MUM1	SMEK1	MYO6
CRYAB	SF1	TCEA1	RCOR1	HNRNPC	LARP1
SNHG6	ZEB2	SAFB	SART3	CKS2	CCNL2
RPL18	TRIM16	NAP1L1	TRPS1	ZNF791	SRRM1
FNBP4	AC005154.6	DR1	NCOR1	SAFB2	RHOQ
IGFBP5	OIP5-AS1	BUB3	SNAPC1	KIF15	UBXN1
CLK1	NUB1	FAM49A	BRD8	RIMKLB	ACIN1
DDX3Y	RUFY3	TSC1	SH3YL1	VEGFB	CDKN2D
RBM39	PTBP1	G3BP1	MIS12	PET100	SET
BTG1	FAM179B	SUPT5H	SP100	SLC20A1	TMSB15A
TAOK1	DDX24	FAM111A	ALG13	VDAC2	NAA15
ASNS	PPIP5K2	PUM2	GOLGA3	DDX27	PFDN4
BOD1L1	TTC3	JMJD6	WDR6	KLF10	XRCC2
PTMA	CDK5RAP3	MRPL33	TSHZ2	UQCRB	UACA
KDM3A	CYFIP2	ECT2	MPLKIP	CD9	GLTP
HILPDA	MLH1	CCP110	KDM5B	POLR2I	SLC5A3
P4HA1	ING1	SPRY1	MED29	SLC16A1	ANKRD49
COX5A	G3BP2	MAT2A	FAM50A	MCL1	GPHN
LMO4	PLEKHB2	C6orf48	RALY	RBM33	HNRNPUL2
BHLHE40	ORC6	MIDN	PKN2	RBM5	TNRC6B

PTP4A1	WNK3	ZNF638	STIM2	LAMTOR3	PCM1
NDUFB10	CABIN1	REV3L	GS1-259H13.2	TOMM20	KCNQ2
PLK2	ZC3H15	MT3	B3GAT1	IRF2BP2	SLC25A12
SHMT2	TIA1	PHF17	TNIP1	RP11-304L19.5	TNPO3
TRA2A	RAB5C	PTK2	DCP2	ZDBF2	AGO3
ZNF90	SHOC2	SUN1	SMARCA2	YIPF4	OLFM1
COX7A2L	SCAF4	MOB1A	ILVBL	LMO1	WDR33
POLR2L	SUMO1	NUP54	SPRED1	CIR1	DCAF16
PDK1	CCDC15	ORC4	CEP350	PRC1	PRMT1
HERPUD1	TRIM44	HLTF	KIAA1143	CHD2	MAN1A2
PNN	SAMD8	OLA1	ZNF326	SERPINE2	EME1
DHX36	PHIP	CCNC	SUV420H1	PAPOLA	SMC5
SLC25A36	IRS2	VAPA	YKT6	NUMA1	MORC4
ARRDC3	HDAC3	SRP54	AP1G1	ZMAT1	EP300
HSPE1	PRKD3	ZC3H7A	IQSEC1	MORF4L2	NAA16
H1FX	PPME1	HIST1H1D	EWSR1	MSL1	TIAL1
NUPR1	SRP68	TBL1XR1	GGNBP2	EEF1B2	WAC
TCF4	GTF2F1	HSPA13	KLF9	TMED7	PIH1D1
HIST1H1C	ACBD3	GPBP1	ZNF580	CCDC14	COA5
HNRNPA1	ZBTB11	ANLN	PISD	SLC44A2	SIKE1
PIN4	SERINC1	MAP3K2	NLRP1	EIF2S2	TNC
ID1	WDR54	MKLN1	TOR1AIP1	EIF2A	PTCH1
RBPJ	YTHDF1	PARVB	CNOT1	KIAA1715	CPSF3L
PCBP2	MIR210HG	PRPF38B	LLPH	UBA2	INTS2
PSMA6	TMEM59L	UBE2B	BMS1	TM7SF3	VOPP1
TMSB10	ASH1L	MAP4K4	DDX54	RPL5	CAMSAP2
LUC7L3	POLK	TRIB1	PPP4R2	PLEKHH2	SLC25A37
YTHDC1	AFAP1L1	HNRNPD	PPIL4	SYF2	TMEM55B
TMEM258	ZFYVE20	UBR5	MED6	KMT2C	DIP2C
SPATA22	KNSTRN	WDR60	SETX	HMOX1	MAP4K5
RPL3	CD99	TCEAL3	BEND5	RPL10A	GADD45A
RPL13	GAPVD1	AHNAK	KIAA1731	ADAMTS1	EHMT2
CCT4	EPC1	MMADHC	GNL3	RP11-639B1.1	TSR3
COX6B1	CHPT1	SREK1	GMPS	COX16	COL6A1
CEBPG	SESN1	RBM28	FAM168B	CLASP2	IGBP1
HNRNPUL1	SLC4A7	PFKP	SUMO2	TMED2	ZNF528
MYADM	SMARCD3	PSMC3IP	KIAA0753	LRRC58	METAP1
TAF1D	SCD	ZNF667-AS1	CNTRL	RLF	NIPSNAP1
DHRS2	DPH3	SNRNP27	DCAF11	MIS18BP1	CHD9
BDP1	TOP2B	TRIM2	OXSR1	APP	MOB1B
PNISR	ATP11B	SRGAP3	CTTN	TMEM68	TNPO2
ERO1L	BPTF	PDCD6IP	ATP6V1C1	NOM1	TNKS
UQCRFS1	UBN1	LTA4H	RUFY2	CRLF1	FAM102A
BNIP3	TMEM159	HELLS	RARS	NDUFA3	YAP1

ZNF207	LONP1	CDV3	AP3B1	KMT2A	ZNF138
TMEM45A	ABHD13	CHORDC1	CTSD	SYNE2	ARFGAP3
ANGPTL4	SFSWAP	MED13L	CASP8AP2	PPM1D	RC3H2
RYBP	BRIX1	CENPC	ADAM10	GCC2	AP000350.4
CCNI	GXYLT2	EEF1D	SLMAP	BAZ2B	BTBD7
RSRC2	RQCD1	ZFP36L1	OFD1	ZNF451	GATAD1
ZNF91	LAMC1	LINC00657	OBSL1	COX5B	SLC12A2
PFDN5	SETD2	METTL12	MLTK	MAP1LC3B	KAT6A
ATP5L	FST	MLF1	NGDN	RBBP6	ASAP2
TRIB2	HOXC10	MAP3K13	IFI6	DMTF1	PDCD7
MGEA5	GM2A	DDX17	THAP6	CEBPZ	AFMID
HSPA9	FRA10AC1	RPL7A	KDSR	SESN2	NLGN1
LINC00152	HIPK1	MSI2	N4BP2	LAMB1	DNAJB6
FBXW7	ILF3-AS1	UTP6	ZDHHC6	RPL7	ZNF200
SLC25A6	RNF145	C12orf23	UFSP2	SERPINH1	BTF3
MSMO1	GIGYF2	CASC3	SSH2	GAS5	ZNF428
EMD	NMRK1	ZBTB20	MCMBP	LRPPRC	GOLGA2
ZFAND5	AC002454.1	FANCL	CTA-29F11.1	TSN	EPHB3
PPP1R3C	EPDR1	SSFA2	RAD1	MFAP4	MAP3K1
EIF1	NFE2L1	RSRC1	IST1	PRELID1	MLLT10
OSER1	C2orf49	RAB5A	MZT2A	NDUFA5	DNAJC10
CWF19L2	HK1	SRSF11	TRIM13	AZI2	FEM1B
ERH	NAA50	SEC31A	HMGXB4	YBX3	TBC1D23
HBP1	NDUFS4	UBQLN1	RNF213	REV1	RNF168
EEF1A2	CCDC66	ZNF263	PIK3C3	RSL1D1	RAD18
IARS	TPD52L2	SRRM2	FBL	MPHOSPH8	MAP7D2
CHMP2A	PLA2G12A	CDK13	ZCCHC6	CDCA5	CTDSPL2
ID3	STT3A	HNRNPA3	PRPF8	DDX18	PMS1
TMF1	KIF1A	DICER1	LSM5	NADK2	FMNL2
QKI	RABGAP1	BRD4	TRNT1	VAMP2	PLEKHA3
NBEAL1	SNRNP200	CCNK	NUP155	SGOL2	SLC16A9
GPCPD1	NCAPH2	SAP30	PTPN12	CMBL	MFGE8
ID2	CEP68	CLIC2	PRRC2B	YWHAG	CCDC18
NT5C3A	SRSF7	ATF2	FAM208A	SNHG7	CHD3
TIPARP	SELK	C1orf52	GTF3C4	SNRNP70	CHCHD7
HSP90AA1	URI1	CCDC93	CHD7	C6orf62	TRIP12
GOLGB1	EIF3F	CCDC80	HIBCH	ROBO1	CA11
SMC4	RALBP1	METTL14	TOR1AIP2	NUDT21	NFRKB
PKIA	USP48	BLOC1S2	CCAR1	TTC17	SKIV2L2
IPO5	DNTTIP2	PPIL3	UTP14C	NFIX	TPM4
RBMX2	GATAD2B	NSUN2	SMIM15	ARL4A	MTX3
C12orf5	TIMM44	DDX55	CEP152	MCM7	SRRT
KIF9	MAT2B	CTDNEP1	FXR1	BRK1	FSTL1
MKLN1-AS1	VPS26A	YIPF5	RIOK3	LTBP4	THOC1

NMD3	PCNT	N4BP2L2	CCDC59	SEL1L	CENPJ
LTN1	FKBP15	ZNF667	NUFIP2	ARL8B	PGAM1
TSC22D3	DIDO1	SOS1	RPN1	UPP1	BZW1
DHX15	NDRG4	POLDIP3	CELF1	CHD1	MCM3AP
GPATCH2L	USP8	CHAMP1	EXOSC9	YY1	BRCA1
TNRC6A	CSPP1	TXNL1	ELAVL1	CLSPN	WHSC1
RANBP1	CD27-AS1	SETD3	TUG1	IWS1	GEM
CAPZA2	LONRF2	TSPYL2	RAP1B	COIL	STAT3
U2AF1	TGS1	HEXIM1	FANCG	POU3F2	ADAMTS9
ZNF146	ARHGEF12	NXF1	MRPL10	TCF25	ZFP36L2
C3orf38	XRCC1	TXNL4A	LCORL	CRTAP	MAR6
TMEM38B	SFXN1	TTK	PARP2	PARL	GNPTAB
NOL11	AIP	NFX1	RNF126	GLUL	BRD2
ALKBH7	CWC27	RTFDC1	CDKN1B	MTF2	HELZ
GNAI2	SENP6	LDLR	HSPA4L	LYST	BST2
COMMD2	ZNF608	RNASE1	HNRNPA0	KIDINS220	CACNG8
METRNL	IFT20	MAPK1	GFPT1	FUBP1	C20orf194
ZNF330	NEDD1	TCERG1	TDP1	SMEK2	NCAPG
TACC3	TRAF3IP1	ATRX	TTTY15	BTG2	MIR4435-1HG
EIF5B	DBNL	THOC2	AC114803.3	COQ10B	ZNF75A
PPP1R2	PHF20	ARHGEF2	DCP1A	JOSD1	IMPDH1
GTF3C3	PRPF4	VHL	ADM	BLOC1S6	PABPC4
AFTPH	LGALS8	CCDC88A	EPS8	NCOR2	FAM63B
DDX46	USF2	TRIP11	ASPM	KIAA0430	WDR89
KIF20B	WAPAL	SRSF5	ZNF277	MTA1	SBNO1
PIAS2	GUSB	LZTFL1	LMNA	VAT1	THAP5
MKRN1	DAAM1	SLC35F5	RAE1	SYNE1	LSM6
RCN1	TMEM189	SMIM4	CEP135	SLC25A38	TAF15
CDCA2	CCDC181	C7orf55-<br>LUC7L2	VPS13C	ST13	USP15
ARID4B	IP6K2	SLC2A4RG	RMND5A	PHTF2	LSM7
OST4	EXOSC5	CEP57	SNHG5	KIAA1109	ZSCAN18
FBXO11	PAK3	NONO	SIAH2	PTPRF	BUB1B
IER5	PPFIA1	NSA2	TAF7	FAM210A	UIMC1
ARMCX2	CCDC50	FER	LSM14A	GTF2I	LUC7L
HIST1H2BD	ZYG11B	UBA5	SIK3	HNRNPH1	SLC38A2
PSMC1	CHAF1A	NEMF	WTAP	TMEM256	WDR82
PCNX	EMC9	NSRP1	TRA2B	SBF2	RNF6
GTF2A1	TPR	PRPF38A	STC1	MPHOSPH9	7-Mar
PTOV1	ANKRD26	MSRB2	OAZ1	C19orf43	ATG12
MBIP	LRRFIP2	ATAD5	RHEB	PLEKHA2	BAG5
FAM118A	SP110	CEP290	ARGLU1	DUSP11	IFI44L
TRIAP1	TSGA10	IL13RA2	IFRD1	OPA1	EIF4E
SNX13	SUMO3	PCF11	NDUFB5	SLC11A2	SEC23IP
MPHOSPH10	SEC63	SNHG8	AK4	AC013394.2	POU3F3

HRK	FKBP10	GPR155	ZNF433	GPR137B	IFI27L1
PRRC2C	ISY1	ARHGAP21	LINC00461	MAX	DDX42
DYNC2H1	KAT6B	ZC3H11A	SNRPF	METTL17	UGCG
ANKRD10	PRKAG1	EIF3H	BUB1	TMEM251	ANKRD54
CDK5RAP2	SNRPA1	KBTBD2	XPC	TM2D1	AGAP1
PFDN2	DYNC1LI1	SRSF6	ATXN2	DYNLL2	

**Intersect with RNAi screen (n=10)**
NRN1
RPS27A
PDK1
RPS6
CCNB1IP1
KIF15
CTNNB1
MAP3K2
CASP8AP2
THAP5

Oligo (n=1659)

EGR1	CLGN	DARS	SF3B14	ASCC3	KLHDC10
FAM162A	CSDE1	NFE2L2	FRYL	PRDX6	AC093323.3
FOS	SSR2	ATAD2	USP46	UBE2B	BAMBI
MALAT1	BRCA1	SAT1	CALCOCO2	PPP1CC	EIF3B
MEG3	WHSC1	SLC4A4	ARIH2	MAP4K4	PFKM
NEAT1	STAT3	SRSF1	DDR2	PMPCB	DDX6
PGK1	C12orf75	DAZAP2	SIRT2	TRIB1	ZNF577
WSB1	UBE2G2	RIF1	CBS	UBR5	NUP214
ZFAS1	DZIP1	HNRNPC	CNTLN	WDR60	EFNB2
EEF1A1	ESCO1	CKS2	CCDC174	GTF2B	SMCHD1
GDF15	FNDC3B	ZNF791	KIAA0020	DPYSL2	ARFGEF1
HMGB2	APOPT1	SERINC3	CPE	MMADHC	ZFAND2B
ATF5	LINC00963	PRCP	SAV1	SREK1	LAS1L
DDIT3	TNFRSF10B	SAFB2	ARID4A	RBM28	CTSL
CDKN1A	YWHAQ	HAT1	CHMP2B	MED10	IQCG
GLTSCR2	NCAM1	VEGFB	RP11-166D19.1	CDIP1	NPLOC4
VIM	ANAPC16	PET100	BTBD1	PFKP	KARS
EIF4A2	COPS2	NAPA	SP140L	FBXO7	SMG7
NRN1	KEAP1	SLC20A1	AP3S1	ZNF667-AS1	DIAPH2
PPP1R15A	CUL3	VDAC2	CWC22	PSAP	ZBTB4
FAU	HNRNPH3	STRADB	PNMAL1	SNRNP27	PTPN13
GOLGA4	NECAP1	PPP2R1A	SMAD5	GAD1	ZMYND11
H3F3B	NDUFA13	LIG1	FTSJ2	CAPRIN1	WBP2
ENO2	SEC61A1	DDX27	USP9X	EZH2	KPNB1

IER2	DLG1	UQCRB	GORASP2	TRIM2	KCTD10
IGFBP2	HEXB	CD9	EIF4A3	NUDT16	KIAA0947
FAM211A	HNRNPDL	C1D	SPG20	SRGAP3	NMI
EEF2	PRPF4B	KHDRBS1	FARSB	BAX	COPRS
TMEM66	FOXN3	SLC16A1	CCDC104	HELLS	SPATA20
FTH1	TCEAL8	PPP2CB	TMEM30A	CDV3	ZNF259
CDK6	CCDC34	MAPK1IP1L	STAM2	MED13L	KDM4A
SRP9	SCFD1	RBM5	DZIP3	CCDC136	CNOT4
SNRPD2	CYP2U1	LAMTOR3	CDC37	ZMYM2	PSMD10
C17orf76-AS1	DDX3X	TOMM20	SF3A1	NACA	KANK1
UBA52	TP53BP1	RP11-304L19.5	APEH	CENPC	ANTXR1
EIF4B	UBAP2	SEPHS2	ACN9	IDI1	WDR43
RHOA	SBDS	ZDBF2	DNPEP	EEF1D	PRPSAP1
GNAS	DNAJB2	PRKAR1A	PRRC1	PSMD3	TBC1D5
FTL	KDM5A	YIPF4	PARP14	RAB1A	GNG2
EIF3E	EIF4A1	LMO1	OSGEP	LINC00657	EIF3M
CCNL1	KPNA1	RRM2B	VEZF1	METTL12	MAGEH1
ODC1	NUPL2	CIR1	UBE2D2	MLF1	THRA
ATF4	TMEM9B	PRC1	KLHDC2	FAM3C	SLC6A8
RBM25	METTL7A	CHD2	BCHE	MAP3K13	NPAS2
WRB	STMN4	SERPINE2	MAP1LC3A	DDX17	RGS10
EPB41L4A-AS1	APPL1	PAPOLA	C16orf91	TMEM59	MAD2L1BP
MDM2	VKORC1	NUMA1	TLK1	MSI2	THAP1
ALDOA	ZFAND2A	CCNT2	BFAR	UTP6	BSG
EMC10	PHYH	ZMAT1	MYO6	C12orf23	PRKCI
TOP2A	DPYSL4	MORF4L2	ZNF622	CASC3	C17orf85
DNAJB1	CCDC58	NDFIP1	LARP1	HN1L	CEP78
NKTR	CETN3	MSL1	CCNL2	UBN2	WASF2
ATF3	GNB1	EEF1B2	SRRM1	CRMP1	LARP4
VEGFA	SSR1	SLC44A2	PTPRG	RAB7A	UHRF1BP1L
H1F0	THSD7A	PTGES3	C8orf4	SSFA2	KTI12
INSIG2	GDI2	SCARB2	RHOQ	UFM1	PPP4C
SEC61G	CRYZL1	KIAA1715	UBXN1	RAB5A	DDX41
SNHG1	ZBTB43	PLEKHH2	ACIN1	SRSF11	EPC2
XPO1	FAM134A	SYF2	SNX17	SEC31A	CAB39
KMT2E	NFYB	RABGGTB	SF3A3	UBQLN1	MFSD6
ZMAT3	TIMM9	ZNF160	NAA15	USP1	LINC00116
PFN2	TMEM55A	KMT2C	PFDN4	SRRM2	PHF14
TYMS	PXDN	ANXA5	NENF	CDK13	OPTN
PRNP	SF3B1	ADAMTS1	XRCC2	HNRNPA3	MAP4
SLC3A2	SARS	PEA15	UACA	TMEM167A	MAGI1
PCLO	NAA25	TMED2	FNDC4	DICER1	SAPCD2
PLOD2	ZBTB25	LRRC58	PTP4A2	BRD4	CPSF4
GNB2L1	CLCN3	JAM2	YWHAZ	CCNK	LSG1

UBC	GPRC5B	CCNG1	SLC5A3	CA12	RCOR1
HSPA8	ACAP2	RLF	AMD1	SAP30	SART3
CRYAB	CHD6	FEZ2	ANKRD49	C1orf52	TRPS1
SNHG6	KPNA4	GRSF1	HNRNPUL2	CCDC93	TIMM21
FNBP4	AKAP17A	MIS18BP1	TNRC6B	CCDC80	NCOR1
RRM2	NCAPD3	APP	BPGM	METTL14	INTS12
PHGDH	SIPA1L2	NOM1	PCM1	USP15	LINC01003
IGFBP5	QSER1	AMZ2	KCNQ2	GNAI3	FAT1
DDX3Y	CXCL16	WDR1	OLFM1	HIST1H2AC	HEBP1
RBM39	RNF181	KMT2A	ILKAP	NSL1	BRD8
BTG1	ARL1	SYNE2	FBXO3	DPM1	MIS12
GADD45B	NFATC4	GCC2	ZNF644	FHL1	SP100
PCNA	SF1	VMA21	ATP6AP1	SSR3	PDCD10
TAOK1	YTHDF2	BAZ2B	WDR41	MCM4	ALG13
SPRY2	OIP5-AS1	MTX2	WDR33	EPS8	C10orf35
HSD17B12	DAP3	ISCU	RPF2	LMNA	GOLGA3
ASNS	COPB2	GOT1	PHLDA1	RAE1	EXOC7
BOD1L1	RUFY3	PAM	DCAF16	DHRS13	TSHZ2
KDELR2	DUSP12	BIRC6	PRMT1	VPS13C	KDM5B
PTMA	FAM179B	MAP1LC3B	CEP44	ACSL3	MED29
KDM3A	DDX24	RBBP6	RAD54L2	HNRNPA0	PDCL
HILPDA	METTL9	DMTF1	SMC5	PKIA	IBTK
GAPDH	PPIP5K2	CEBPZ	TTC9C	COPS7A	PKN2
P4HA1	TTC3	LAMB1	SNX4	RPA1	STIM2
LMO4	CD164	CREB5	CARS	C12orf5	GS1-259H13.2
MTHFD2	CDK5RAP3	SERPINH1	MORC4	KIF9	PRELID2
BHLHE40	CYFIP2	CENPE	EP300	SAR1B	PLIN3
PTP4A1	MLH1	GAS5	NAA16	TMEM14A	FAM84B
ITM2B	RRAGC	LRPPRC	TIAL1	C16orf80	TNIP1
SHMT2	RNASEH2B	TSN	NADSYN1	C7orf41	PAFAH1B1
TRA2A	ACTL6A	EI24	WAC	TSC22D3	PUS7L
ZNF90	C10orf118	NDUFA5	CRLS1	DHX15	PPID
TXNIP	TFDP1	TMPO	SIKE1	GPATCH2L	WDYHV1
ATXN10	G3BP2	CBX3	CYB5D1	TNRC6A	KCTD7
PPP1CB	PLEKHB2	AZI2	TNC	CAPZA2	NPM3
COX7A2L	ZC3H15	ACTR2	EFCAB14	U2AF1	NFKBIB
PDK1	ANG	YBX3	CCNH	PPP2R3C	SPRED1
HSPA5	POLR2B	RSL1D1	XAF1	ZNF146	XIAP
HERPUD1	C3orf17	AK3	PTCH1	PRIM1	CEP350
PNN	TIA1	ARPC4	RBAK	NOL11	BROX
DHX36	COMMD8	LL22NC03-2H8.5	VOPP1	GNAI2	KIAA1143
BNIP3L	SCAF4	DDX18	ZFAND1	ZNF330	CEP112
SLC25A36	LBH	VAMP2	SPDL1	SEC11A	GALNT7
WARS	SUMO1	COPS3	CAMSAP2	PPP1R2	ZNF326

ARRDC3	TRIM44	JMY	POLR2H	ATP5EP2	SUV420H1
H1FX	ZNF800	CMBL	MPRIP	KIF20B	YKT6
PABPC1	NOL3	VGLL4	ATCAY	ALDOC	STAT1
ARF4	PHIP	YWHAG	SNX1	PIAS2	MUT
NUPR1	GINM1	SNHG7	SLC25A37	MKRN1	CREB1
TCF4	IRS2	HNMT	TMEM55B	RCN1	IQSEC1
HIST1H1C	PRKD3	SNRNP70	MAP4K5	ARID4B	TSG101
MDH1	BACE2	BEX4	CERS6	PEX2	EWSR1
HNRNPA1	PPME1	C6orf62	GADD45A	PSMC1	KLF9
ID1	SRP68	ROBO1	COL6A1	CD47	PISD
SELT	NUCB1	HPS4	KCTD5	PCNX	CECR5
ANXA1	MYCBP2	NUDT21	CAPN7	ARC	LETM1
RBPJ	GTF2F1	APLP1	IGBP1	PTOV1	PCGF2
COPS8	ACBD3	LUC7L	ZNF528	MBIP	NOTCH2NL
UBE2E1	TBCD	NPM1	GYG1	PTCD3	POLR1D
PCBP1	WDR54	TEX30	DDX39A	FAM118A	C8orf44
PCBP2	TMEM59L	NDUFB5	VPS26B	TXNDC9	KLF3
RNF187	ASH1L	AK4	NIPSNAP1	TLE1	DDX54
CNIH1	ZFHX4	ERP44	CHD9	MAP2K2	PPP4R2
RPSAP58	CD99	KIAA0101	DNAJC13	TRIAP1	PPIL4
TMSB10	GAPVD1	LINC00461	ATPAF1	SNX13	ALG2
LUC7L3	EPC1	TAF1	YAP1	MPHOSPH10	MED6
YTHDC1	SLC4A7	XPC	ZNF138	ARHGEF7	RFC1
GARS	SRP72	FXR1	ARFGAP3	HRK	SETX
CENPF	SMARCD3	CCDC59	C19orf66	PRRC2C	ZSCAN16-AS1
HNRNPF	SCD	HNRNPH2	PRKCSH	DYNC2H1	BEND5
CCT4	PSMD6	2-Sep	UBALD1	ANKRD10	POLB
CEBPG	CYBRD1	NUFIP2	FAM161A	CDK5RAP2	KIAA1731
HNRNPUL1	ARMC8	RPN1	BTBD7	DYNLL2	GNL3
HADHB	DPH3	CELF1	GATAD1	AC013394.2	HSF1
CLDND1	HBEGF	PMAIP1	UBR4	TXNDC12	PHF20L1
LAPTM4A	MEAF6	CLTC	KAT6A	AC064875.2	FAM168B
TAF1D	TOP2B	RNMT	ASAP2	BLOC1S2	C2orf15
BDP1	ZNF581	MARK3	ACBD5	PPIL3	PBX1
PNISR	BPTF	ZCCHC11	ASB8	NSUN2	UBA3
MAD2L1	UBN1	HNRNPK	LDLRAD3	TWF2	SUMO2
SQSTM1	TMEM159	PPDPF	PDCD7	VDAC1	KIAA0753
BNIP3	LONP1	TXNRD1	ABHD10	DDX55	CNTRL
ZNF207	SAE1	GHITM	MANBA	CTDNEP1	OXSR1
	SFSWAP	DKK3	NLGN1	SEC22C	CTTN
ANGPTL4	BRIX1	STRAP	DNAJB6	ARPP19	ATP6V1C1
RYBP	NGLY1	SSBP2	TPRKB	AAMP	RUFY2
CCNI	SKAP2	LRRN3	BTF3	PCSK7	RARS
RSRC2	DALRD3	CTNNB1	PAFAH1B2	CXXC5	MTA2

ANKRD36C	LAMC1	FKBP4	ZNF428	SRSF9	AP3B1
LAMP2	RP11-468E2.2	EIF2AK2	RPGR	GNAI1	SAMD9L
TFG	CTD-3018O17.3	PRKAG2-AS1	MLLT10	N4BP2L2	CTSD
ZNF91	CPT1A	FGFR1OP2	KIF1B	SOS1	CASP8AP2
PPT1	DNAJC25	SLU7	DNAJC10	POLDIP3	DDX56
PFDN5	SETD2	ABI2	DNM1L	CHAMP1	UVRAG
TRIB2	HOXC10	FAM49B	TBC1D23	MAFG	OFD1
SPTSSA	FRA10AC1	FAM133B	AC005152.2	TXNL1	OBSL1
MGEA5	CNIH3	CCT8	CD70	TSPYL2	MLTK
HSPA9	SOX9	C17orf58	RNF168	HEXIM1	RARS2
FBXW7	SEMA6D	AES	CSPG5	MAGED2	NGDN
MSMO1	ILF3-AS1	MZT2B	RAD18	ARL5A	KDSR
EMD	RNF145	NPEPPS	SNX2	ARMC10	N4BP2
ZFAND5	GIGYF2	NFIB	CTDSPL2	HMGN1	NR2F1-AS1
GNB2	RELA	MID1IP1	CDK11B	LDLR	UFSP2
ENOPH1	AC002454.1	MNS1	PELO	DUSP4	SSH2
EIF1	UBQLN2	ACTR3	FAM172A	RNASE1	CTA-29F11.1
MZT1	NFE2L1	NDUFC1	PMS1	AKAP11	SLC25A14
PPP2R5C	C2orf49	JUN	FMNL2	ZC3HAV1	MZT2A
SDCBP	HK1	EAPP	CLN5	TEAD1	PARD3
OSER1	CDK12	AKAP9	PLEKHA3	MAPK1	HMGXB4
CWF19L2	FXYD5	GAS1	GNS	BNIP2	RNF213
TSPAN13	SMIM7	RBMX	PYGL	TCERG1	PIK3C3
IARS	CCDC66	TTC14	CCDC18	ATRX	FBL
ID3	TMEM245	AKAP8L	RBFOX2	CAMK2D	ZCCHC6
CYCS	TPD52L2	TRMT112	CHD3	THOC2	PRPF8
TMF1	PLA2G12A	FAM210B	TRIP12	IL6ST	RAB4A
QKI	SNHG12	IMPDH2	CA11	C19orf48	ZC3H8
NBEAL1	KIF1A	TCP1	KIAA2026	CCDC88A	KIF16B
GPCPD1	FAM206A	DDB2	NFRKB	TRIP11	STRBP
DDX5	SMG6	TRIM4	RBM48	SRSF5	PTPN12
ID2	RABGAP1	CTNNA1	WDR82	SCAMP1	PRRC2B
NT5C3A	RMDN3	PPP2CA	RNF6	ARHGAP12	GTF3C4
HSP90AA1	SNRNP200	C11orf96	ADAM17	DCTN2	CHD7
GOLGB1	CEP68	ZNF83	MTR	LIMS1	TOR1AIP2
SMC4	SRSF7	CD81	MDN1	C7orf55-LUC7L2	THAP5
RNF13	SOX11	DRAXIN	TRAPPC2P1	SRPRB	CACNG8
RAD23A	SELK	UBXN7	SRRT	MAPRE1	ATXN2
ZFP36L2	EPRS	DST	AC074289.1	CEP57	PSMD13
GGH	URI1	MOB4	PDHX	NONO	POU3F3
ADM	EIF3F	PPIG	TP53	NSA2	VPS53
TRA2B	YWHAH	IFI27L2	WNK1	FER	MAX
STC1	RALBP1	SPAG9	KLHL42	FAM120A	ANAPC5

SNX3	DESI2	CRBN	CKAP4	UBA5	GALNT11
OAZ1	RAB39B	EIF4H	CENPJ	SPTY2D1	PWWP2A
RHEB	MAEA	BRWD1	PRPF3	NEMF	SKIV2L2
ARGLU1	UIMC1	DLX2	BAG4	CEP290	USP48
IFRD1	PITHD1	RAD23B	PGAM1	ANAPC13	DNTTIP2
CDKN1B	BRD2	MYEF2	EXOSC9	IL13RA2	UBR1
CIRBP	RMND5A	YARS	ELAVL1	SGCB	OTUD5
RSL24D1	SNHG5	DNASE2	TUG1	PCF11	CLUAP1
ZNF844	EXOC5	AKIRIN1	ATM	IFT52	TIMM44
RBM6	TAF7	UBE2D3	PAAF1	MKI67IP	MAT2B
PNRC1	FAM32A	RANBP2	ATP5SL	SNHG8	VPS26A
BTG3	LSM14A	RAB21	PYGB	GPR155	IMMP2L
FSCN1	SIK3	NDFIP2	ARHGEF26	PSPH	NUP88
PCNP	PARP2	ATP6V1H	WDR48	ATP2A2	PCNT
HPRT1	FAM122A	PRPF6	CASK	ARHGAP21	CCDC173
KLF6	TRIM52	GYPC	GON4L	UPF2	DIDO1
STARD3NL	PNMA1	BET1	FGFR1	ZC3H11A	NDRG4
DDIT4	C11orf54	LINC00998	NOLC1	EIF3H	CSPP1
PAXBP1	NT5DC2	MAF	NLGN4Y	KBTBD2	CD27-AS1
NAMPT	TM2D3	PURB	ZNF711	SRSF6	LONRF2
ZNF292	FYTTD1	ATP6V1A	NARF	CCDC115	UBAP1
ALKBH5	TFRC	CWC25	PIBF1	DDX42	TGS1
FDXR	YTHDC2	TCEA1	PHF3	7-Mar	ARHGEF12
MXI1	EP400	SAFB	UBFD1	ATG12	XRCC1
XPOT	RP11-732M18.3	NAP1L1	PTPLB	SOX2	AIP
MLF1IP	WDFY3-AS2	PSMC2	BIRC2	BAG5	CWC27
SON	NEU1	MFF	TTLL7	TTC17	SENP6
HIGD1A	PBRM1	ORMDL1	LPIN1	CFL2	SPG21
CNBP	SNHG14	BUB3	C12orf29	NFIX	IFT20
NOP58	WDR70	CDC27	PRPF40A	ARL4A	LRP6
SETD5	NKIRAS1	FAM49A	AIMP1	DUSP14	CSNK1E
TSC22D1	DIAPH3	TSC1	TOMM70A	CDC42BPA	EPB41L1
TLK2	LETMD1	G3BP1	SSBP4	MCM7	SPG7
DLX1	ZNHIT6	BAZ2A	BCAS2	BRK1	DBNL
EIF1B	HGS	TET1	GLRX5	TANK	RNASEH1
SRSF2	LRIF1	SUPT5H	BRI3	SEL1L	R3HDM2
SNHG16	POM121C	PRPSAP2	IGF2R	APMAP	PRPF4
HES1	UNC119	FAM111A	LBR	ARL8B	SRFBP1
P4HB	PLCG1	BCL7B	PGRMC2	CHD1	VPS8
COPS4	ZC3H6	PMP22	PDCD11	YY1	LGALS8
TPX2	SLC22A17	C6orf48	RP9	IWS1	ZHX1
ITSN2	USP33	MIDN	EIF2S3	HDGF	USF2
WDR45B	FRG1	PHB2	SUGP2	COIL	WAPAL
SCOC	THUMPD1	ZNF638	ATXN1	SENP2	GTF2H1

HEY1	LSM14B	STX16	CDC16	VAPB	GUSB
PDIA6	RAB3GAP1	REV3L	USP14	CARHSP1	DAAM1
COX7C	CCDC84	MT3	CFDP1	TCF25	HPS1
PREPL	COPB1	TMEM128	TLE4	CRTAP	MEGF8
SERTAD1	MAGOH	PTK2	FAM13B	PARL	AKAP1
TOMM7	PGM3	EPM2AIP1	RAB22A	IPO7	IP6K2
ATP5G2	AAR2	SUN1	ZFYVE21	GLUL	POLR3D
TMEM123	TGOLN2	MOB1A	SYNRG	MTF2	SLC25A17
HSPH1	DEK	TARS	IGF1R	GOLPH3	HABP4
PAICS	SNN	HLTF	ZNF148	MOAP1	ZNF117
ANKRD12	STT3B	OLA1	NTPCR	KIDINS220	EXOSC5
RTN3	YIPF6	VAPA	SLC4A10	FUBP1	SUPT20H
U2SURP	SETD5-AS1	PAIP2	DHX9	SMEK2	PPFIA1
CEP95	IFNGR2	SRP54	TOB2	BTG2	ZNF789
GADD45GIP1	SLC25A26	ZC3H7A	IPP	COQ10B	SOD2
NPC2	CHCHD3	TBL1XR1	DSCR3	DHX40	CHAF1A
KXD1	SPTAN1	HSPA13	RGS17	BLOC1S6	OSBPL1A
ARHGAP5	ZCCHC7	GDI1	CCZ1B	SERP1	FAM105B
BTF3L4	FAM76B	GPBP1	FIP1L1	SUPT6H	B4GALT5
EIF3L	SPTLC1	MAP3K2	STAG1	CELF2	NOB1
RRAGD	PRPF19	MKLN1	GABARAPL1	KIAA0430	STRN
SNX5	C12orf76	PARVB	ZNF721	VAT1	TPR
ZFC3H1	BRWD3	PRPF38B	SFPQ	SYNE1	POLR1E
PDGFRA	MRFAP1L1	GPI	MECP2	FAM210A	CREBL2
PLRG1	ANKRD26	SGCE	MOK	COPZ1	ZNF219
RP11-220I1.1	URB1	C19orf43	AKAP8	EIF2S1	C5orf42
ST13	POM121	EVL	CMAS	HNRNPH1	FKBP10
CDK14	LRRFIP2	GLOD4	MBD4	EIF3D	KAT6B
MTCH1	LINC00667	PRKRA	SMIM15	RFK	DYNC1LI1
PHTF2	SP110	OPA1	FAM63B	GPT2	ANKRD54
SEPP1	NAA35	PCID2	TMEM33	TMEM256	FLOT2
SAR1A	TSGA10	SBNO1	SNHG17	ACTR10	MFAP1
KIAA1109	MAD1L1	PCYOX1	TPM4	MPC1	AGAP1
PTPRF	SEC63	PABPC4			

**Intersect with RNAi screen (n=15)**
NRN1
RHOA
ALDOA
UBC
RPS27A
PDK1
RPS6
TPX2

CTNNB1
MAP3K2
TP53
GNG2
CASP8AP2
THAP5
ZNF117

Table S4. List of antibodies.

Antibody	Company	Catlog#	Dilution
ZNF117	Novus	NBP1-79242	1:1000
Beta-actin	BioLegend	664802	1:1000
Nestin	BioLegend	841901	1:100
GalC	Sigma	MAB342	1:100
GFAP	Dako	Z0334	1:100
Tuj1	R&D Systems	MAB1195	1:100
Olig1	R&D Systems	MAB2417	1:100

Table S5. List of Primers.

Primers for ChIP-qPCR	
JAG2-ChIP-For	TCAAAGCCCAGGGTACCCCAG
JAG2-ChIP-Rev	GTCCTGGGCTTTAAGGTGCCC
TBL1X -ChIP-For	CAACGAGAGAACATGTGAAGT
TBL1X -ChIP-Rev	CAGTGAGCTTGTTTCAGGCT
IL6ST -ChIP-For	AGACTCTTGCCTATTAGTGT
IL6ST -ChIP-Rev	CTTCATAATAACTCTATGAAG
CDH4-ChIP-For	GCCGTGAATAAGCCTCTTACG
CDH4 -ChIP-Rev	ACCAGTAACATACCAGAAAGT

Primers for qRT-PCR	
JAG2-For	TGGGACTGGGACAACGATAC
JAG2-Rev	AGTGGCGCTGTAGTAGTTCTC
NOTCH1-For	GAGGCGTGGCAGACTATGC
NOTCH1-Rev	CTTGTACTCCGTCAGCGTGA
NOTCH2-For	CAACCGCAATGGAGGCTATG
NOTCH2-Rev	GCGAAGGCACAATCATCAATGTT
NOTCH3-For	TGGCGACCTCACTTACGACT
NOTCH3-Rev	CACTGGCAGTTATAGGTGTTGAC
GAPDH-For	TCAGCCTGGGGCTGGCATTG
GAPDH-Rev	GGCTGGTGGTCCAGGGGTCT

Primers for sequencing	
ZNF138F	CTTTGCATGCTTTCACGCCT
ZNF138R	GTAAGGGTTGGGGACTGCTT
ZNF85F	GTCTCACAGCTACCCAGAGC
ZNF85R	GGTTTGAGGACTGGTTAAAGGC
ZNF107F	TGACAGCTACCCCAAGCAAA
ZNF107R	GCCTTGCCACATTCTTCACA
SH3PXD2BF	GACAGAAGAGGTTGAGCCCC
SH3PXD2BR	GACCTCGTGTCAGACTGTGG
IGS5F	GTGATGTGACAGAGCCATTT
IGS5R	GGAAGTTGGAAAGCGAAGAT

References

1. Bradshaw, A., *et al.* Cancer Stem Cell Hierarchy in Glioblastoma Multiforme. *Frontiers in Surgery* **3**, 21 (2016).
2. Darmanis, S., *et al.* A survey of human brain transcriptome diversity at the single cell level. *Proc Natl Acad Sci U S A* **112**, 7285-7290 (2015).
3. Singh, S.K., *et al.* Identification of human brain tumour initiating cells. *Nature* **432**, 396-401 (2004).
4. Darmanis, S., *et al.* Single-Cell RNA-Seq Analysis of Infiltrating Neoplastic Cells at the Migrating Front of Human Glioblastoma. *Cell Rep* **21**, 1399-1410 (2017).
5. Lathia, J.D., *et al.* Integrin alpha 6 regulates glioblastoma stem cells. *Cell Stem Cell* **6**, 421-432 (2010).
6. Cenciarelli, C., *et al.* PDGFRα depletion attenuates glioblastoma stem cells features by modulation of STAT3, RB1 and multiple oncogenic signals. *Oncotarget* **7**, 53047-53063 (2016).
7. Zhao, X., *et al.* The N-Myc-DLL3 cascade is suppressed by the ubiquitin ligase Huwe1 to inhibit proliferation and promote neurogenesis in the developing brain. *Dev Cell* **17**, 210-221 (2009).
8. Yan, T., *et al.* Neuronal markers are expressed in human gliomas and NSE knockdown sensitizes glioblastoma cells to radiotherapy and temozolomide. *BMC Cancer* **11**, 524 (2011).
9. Hu, J., *et al.* Neutralization of terminal differentiation in gliomagenesis. *Proceedings of the National Academy of Sciences* **110**, 14520-14527 (2013).
10. Evangelidou, M., Karamita, M., Vamvakas, S.S., Szymkowski, D.E. & Probert, L. Altered expression of oligodendrocyte and neuronal marker genes predicts the clinical onset of autoimmune encephalomyelitis and indicates the effectiveness of multiple sclerosis-directed therapeutics. *J Immunol* **192**, 4122-4133 (2014).
11. Ayanlaja, A.A., *et al.* Distinct Features of Doublecortin as a Marker of Neuronal Migration and Its Implications in Cancer Cell Mobility. *Frontiers in Molecular Neuroscience* **10**, 199 (2017).
12. Tanaka, T., *et al.* CD24 expression as a marker for predicting clinical outcome and invasive activity in uterine cervical cancer. *Oncol Rep* **34**, 2282-2288 (2015).
13. Gunther, H.S., *et al.* Glioblastoma-derived stem cell-enriched cultures form distinct subgroups according to molecular and phenotypic criteria. *Oncogene* **27**, 2897-2909 (2008).
14. Zhang, Y., *et al.* Purification and Characterization of Progenitor and Mature Human Astrocytes Reveals Transcriptional and Functional Differences with Mouse. *Neuron* **89**, 37-53 (2016).
15. Campeau, E., *et al.* A versatile viral system for expression and depletion of proteins in mammalian cells. *PloS one* **4**, e6529 (2009).
16. Concordet, J.P. & Haeussler, M. CRISPOR: intuitive guide selection for CRISPR/Cas9 genome editing experiments and screens. *Nucleic Acids Res* **46**, W242-W245 (2018).
17. Stegmeier, F., Hu, G., Rickles, R.J., Hannon, G.J. & Elledge, S.J. A lentiviral microRNA-based system for single-copy polymerase II-regulated RNA interference in mammalian cells. *Proc Natl Acad Sci U S A* **102**, 13212-13217 (2005).
