# Supplementary material for: ZNF117 regulates glioblastoma stem cell differentiation towards oligodendroglial lineage
Source: Nat Commun. 2022 Apr 22;13:2196. doi: 10.1038/s41467-022-29884-3 (PMC9033827; doi:10.1038/s41467-022-29884-3)
Supplement: Supplementary file 2 — Inventory of Supporting Information [file 41467_2022_29884_MOESM2_ESM.docx]

Inventory of Supporting Information

**Manuscript #:** NCOMMS-21-10451.

**Corresponding author name(s):** Jiangbing Zhou.

1. **Supplementary**

| Figure # | Figure title  One sentence only | Filename  This should be the name the file is saved as when it is uploaded to our system. Please include the file extension. i.e.: *Smith_ED Fig1.jpg* | Figure Legend  If you are citing a reference for the first time in these legends, please include all new references in the Online Methods References section, and carry on the numbering from the main References section of the paper. |
| --- | --- | --- | --- |
| Supplementary Fig. 1 | Quantification of the efficiency in reducing Nestin expression in GS5 cells by delivering siNestin or control siGENOME RISC-Free siRNA. |  | 2x RNAiMax represents the use of two times volume of lipofectamine RNAiMax reagent suggested by the vendor (Invitrogen). 2x RNAiMax, which demonstrates a slightly greater efficiency than 1x RNAiMax, was selected for the genome wide RNAi screen. The mean of data of three biologically independent samples is shown and data are presented as mean values ± SD (n = 3). |
| Supplementary Fig. 2 | Analysis of GS5 scRNA-seq data. |  | **a**, tSNE of scRNA-seq data from GS5 cells. Unsupervised clustering based on top 1,000 variable genes reveals cell-cell heterogeneity within the GS5 tumor population. **b**, Violin plot of individual gene markers used to identify cell types from clusters. *KLF4*^1^, *DPYSL5*^2^, *C16orf89*^2^, and *PLP1*^2^ were used to identify GSCs, neurons, astrocytes, and oligodendrocytes, respectively. **c**, Box-and-whisker plots of canonical gene marker sets used to identify cell types from clusters (the middle, bottom, and top lines correspond to the median, bottom, and top quartile, and whiskers to lower and upper extremes minus bottom quartile and top quartile, respectively). Units are the average number of mRNA transcripts for each gene set per cell. Canonical cell type markers include: *CD133*^3^, *OLIG2*^4^, *ITGA6*^5^, *PDGFRA*^6^, *DLL3*^7^, *OLIG1*^7^ used to define GSCs. *NEFL*^8^, *RBFOX3*^9^, *GRIA3*^4^, *STMN2*^1^, *COBL*^2^, *LIN7C*^10^, *DCX*^11^, and *CD24*^12^ used to define neurons, *GFAP*^13^, *PAQR6*^2^, *PPP1R3C*^2^, *C16orf89*^2^, *ALDOC*^14^, and *AQP4*^2^ used to define astrocytes, and *MAL*^2^, *PLP1*^2^, *MBP*^2^, *MAG*^2^, and *MOG*^14^ used to define oligodendrocytes. Enriched gene score was calculated as the sum of the average number of mRNA transcripts per gene for each cell type. **d**, Cell states of single-cell trajectories. States 1, 3, and 4 were used for GSC to neuron, oligodendrocyte, and astrocyte differentiation, respectively. **e,f**, Venn diagram of significant genes that regulate (e) neurons and (f) astrocyte differentiation from single-cell trajectory analysis and Nestin expression from the RNAi screen. *ZNF117* does not overlap for either cell type. **g,h**, Heatmap of significant genes that regulate GSC to oligodendrocyte differentiation. *ZNF117* decreases in expression as GSCs differentiation into (g) neurons, and (h) astrocytes. Heatmap colors represent normalized gene expression across pseudotime. Genes are hierarchically clustered according to their expression pattern. q-val < 1e-320. |
| Supplementary Fig. 3 | Flow cytometry analysis of FBS-differentiated GS5 cells. |  | Cells were cultured in DMEM medium supplemented with 10% FBS. Two weeks later, the cells were collected, stained with the indicated antibodies, and subjected to flow cytometry analysis. |
| Supplementary Fig. 4 | *ZNF117* regulates GSC differentiation towards oligodendroglial lineage. |  | **a**, Schematic of sgRNA locations in *ZNF117* gene. **b,c**, Flow cytometry analyses of GFAP+ and Tuj1+ (b) and Olig1+ (c) cell populations in GS5 cells after treatment with Cas9 and the indicated sgRNAs. **d**, Ki67 staining of residual tumors isolated from mice received inoculation of the indicated cells. Images are representative of 3 independent experiments. Scale bar, 50 μm. |
| Supplementary Fig. 5 | Flow cytometry analysis of genetically engineered GS5 cells. |  | Cells treated with Cas9/sgGFP, Cas9/sgZNF117, Cas9/sgZNF117 following with *ZNF117* overexpression, and Cas9/sgZNF117 following with *JAG2* overexpression were stained with the indicated antibodies and subjected to flow cytometry analysis. *Jagged 2* (human, BC032053) cDNA and *ZNF117* (Human, BC034021) cDNA were purchased from Horizon Discovery and subcloned into vector pLenti CMV Blast (a gift from Eric Campeau & Paul Kaufman, Addgene plasmid# 17486) ^15^. Overexpression was performed through lentiviral transduction. |
| Supplementary Fig. 6 | Characterization of potential off-target effects of *ZNF117* sgRNA-1. |  | **a**, Bioinformatic analysis of candidate genes carrying mismatched sequences in exons. Analysis was carried out using CRISPOR (<http://crispor.tefor.net/crispor.py>) ^16^. **b-f**, Sequencing analysis did not find significant activities in *ZNF138* (b), *ZNF85* (c), *ZNF107* (d), *SH3PXD2B* (e) and *IGSF5* (f). Upper and bottom panels illustrate the sequences in GS5^sgGFP^ cells and GS5^sgZNF117-1^ cells, respectively. Sequencing analysis did not find expression of *KDM5D* and thus was not included. |
| Supplementary Fig. 7 | Validation of the differentiation effect of *ZNF117* in PS24 cells. |  | **a**, WB analysis of the expression of *ZNF117* in PS24 cells cultured in serum-containing medium or treated with Cas9 and the indicated sgRNAs. **b,c**, Proliferation (n=3 biologically independent samples) (b) and stem cell frequency (c) of PS24 cells treated with Cas9 and the indicated sgRNAs **d-f**, Representative flow cytometry images (d) and corresponding quantification (n=3 biologically independent samples) (e), and immunostaining (f) of Nestin+ and GalC+ cell populations in PS24 cells after treatment with Cas9 and the indicated sgRNAs. Scale bar, 50 μm. g, Kaplan Meier survival analysis of the mice received inoculation of the indicated cells. All data are presented as mean ± SD. *P-value < 0.05; **P-value < 0.01. Statistical differences were determined by two-tailed student’s t test. Images are representative of 3 independent experiments. |
| Supplementary Fig. 8 | Characterization of signaling regulated by *ZNF117*. |  | **a**, Analysis of pathways regulated by *ZNF117* based on whole-transcript expression analysis. **b**, Validation of the expression of Notch 1, 2, 3 in GS5 cells treated with Cas9 and sgGFP or sgZNF117 (n=3 biologically independent samples). **c**, Pathway reporter assay suggests that ZNF117 doesn’t modulate Wnt activity (n=3 biologically independent samples). **d**, Distribution of the *ZNF117* binding regions identified by ChIP-Seq. **e**, ChIP-PCR analysis confirmed *ZNF117* binds with the indicated genes (n=3 biologically independent samples). All data are presented as mean ± SD. **P*-value < 0.05. Statistical differences were determined by two-tailed student’s t test. Source data are provided as a Source Data file. |
| Supplementary Fig. 9 | Database analysis of *ZNF117*. |  | **a**, Analysis of TCGA RNA-seq database suggests that *ZNF117* expresses in a higher level in GBM than normal brain tissues (the middle, bottom, and top lines correspond to the median, bottom, and top quartile). **b**-**d**, Analyses of TCGA RNA-seq database (b), Rembrandt database (c) and CGGA (d) databased suggests that *ZNF117* is negatively correlated with patient survival. Maximally selected rank statistics was used to determine the optimal cutoff for Kaplan-Meier survival analysis as provided in the 'survminer' package in GlioVis (GlioVis data portal for visualization and analysis of brain tumor expression datasets, Neuro Oncol. 2017; 19(1):139-141). Specific parameters used for the analyses include: a&b: Dataset: adult “TCGA_GBM”, Platform: “RNA-seq”, Cutoff value: “optimal cutoff”; c, Dataset: adult “Rembrandt”, Cutoff value: “optimal cutoff”; d, Dataset: adult “CGGA”, Tumor type: “Primary”, Histology: “All”; Cutoff value: “optimal cutoff”. |
| Supplementary Fig. 10 | Imaging of tumor transfection by LHNPs. PS30 cells were engineered to expression GFP and inoculated into the brains of nude mice. |  | Three weeks later, the mice were treated with LNHPs encapsulated with RFP-expression plasmid (pPRIME-CMV-dsRed, Addgene plasmid # 11658)^17^. Three days later, the mice were euthanized. The brains were isolated, sectioned, and imaged. Images are representative of 3 independent experiments. Scale bar, 50 μm. |
| Supplementary Fig. 11 | Characterization of *ZNF117* as a therapeutic target. |  | **a**, WB analysis of *ZNF117* expression in GS5 cells treated with LHNPs loaded with Cas9 and sgGFP or sgZNF117. **b**, Inhibition of the proliferation of GS5 cells treated with Cas9 with the indicated sgRNA by TMZ (n=3 biologically independent samples). All data are presented as mean ± SD. **P-value < 0.01. Statistical differences were determined by two-tailed student’s t test. |
| Supplementary Fig. 12 | Schematic representation of *ZNF117*-mediated GSC differentiation through interaction with *JAG2*. |  |  |
| Supplementary Fig. 13 | A representative flow cytometry gating strategy. |  |  |

1. **Supplementary Information:**
2. **Flat Files**

| Item | Present? | Filename  This should be the name the file is saved as when it is uploaded to our system, and should include the file extension. The extension must be .pdf | A brief, numerical description of file contents.  i.e.: *Supplementary Figures 1-4, Supplementary Discussion, and Supplementary Tables 1-4.* |
| --- | --- | --- | --- |
| Reporting Summary | y | Reporting-summary NCOMMS-21-10451.pdf |  |

1. **Additional Supplementary Files**

| Type | Number  If there are multiple files of the same type this should be the numerical indicator. i.e. “1” for Video 1, “2” for Video 2, etc. | Filename  This should be the name the file is saved as when it is uploaded to our system, and should include the file extension. i.e.: *Smith_*  *Supplementary_Video_1.mov* | Legend or Descriptive Caption  Describe the contents of the file |
| --- | --- | --- | --- |
| Supplementary Table | 1 | Validation of top 100 genes |  |
| Supplementary Table | 2 | scRNA-seq sample information |  |
| Supplementary Table | 3 | Candidates identified by scRNA-seq |  |
| Supplementary Table | 4 | List of antibodies used in the study |  |
| Supplementary Table | 5 | List of Primers used in the study |  |
| Supplementary Data | 1 | Genome wide screen |  |
| Supplementary Data | 2 | cDNA array analysis | Affymatrix Transcriptome Analysis Console (TAC) software was used to analyze expression array data. A 2-sided test was conducted with FDR (false discovery rate) adjusted for multiple comparisons. |
| Supplementary Data | 3 | ChIP-seq analysis |  |

***Add rows as needed to accommodate the number of files.***

1. **Source Data**

| Figure | Filename  This should be the name the file is saved as when it is uploaded to our system, and should include the file extension. i.e.: *Smith_SourceData_Fig1.xls, or Smith_*  *Unmodified_Gels_Fig1.pdf* | Data description  i.e.: Unprocessed Western Blots and/or gels, Statistical Source Data, etc. |
| --- | --- | --- |
|  | Source Data.xls | Source Data for all figures. |
