# Supplementary material for: ZNF117 regulates glioblastoma stem cell differentiation towards oligodendroglial lineage
Source: Nat Commun. 2022 Apr 22;13:2196. doi: 10.1038/s41467-022-29884-3 (PMC9033827; doi:10.1038/s41467-022-29884-3)
Supplement: Supplementary file 7 — Reporting Summary [file 41467_2022_29884_MOESM7_ESM.pdf]

nature research

Corresponding author(s): Jiangbing Zhou

Last updated by author(s): 02/22/2022

Reporting Summary

Nature Research wishes to improve the reproducibility of the work that we publish. This form provides structure for consistency and transparency in reporting. For further information on Nature Research policies, see our Editorial Policies and the Editorial Policy Checklist.

Statistics

For all statistical analyses, confirm that the following items are present in the figure legend, table legend, main text, or Methods section.

n/a	Confirmed	
☐	☒	The exact sample size (*n*) for each experimental group/condition, given as a discrete number and unit of measurement
☐	☒	A statement on whether measurements were taken from distinct samples or whether the same sample was measured repeatedly
☐	☒	The statistical test(s) used AND whether they are one- or two-sided<br>*Only common tests should be described solely by name; describe more complex techniques in the Methods section.*
☒	☐	A description of all covariates tested
☒	☐	A description of any assumptions or corrections, such as tests of normality and adjustment for multiple comparisons
☐	☒	A full description of the statistical parameters including central tendency (e.g. means) or other basic estimates (e.g. regression coefficient) AND variation (e.g. standard deviation) or associated estimates of uncertainty (e.g. confidence intervals)
☐	☒	For null hypothesis testing, the test statistic (e.g. *F*, *t*, *r*) with confidence intervals, effect sizes, degrees of freedom and *P* value noted<br>*Give P values as exact values whenever suitable.*
☒	☐	For Bayesian analysis, information on the choice of priors and Markov chain Monte Carlo settings
☒	☐	For hierarchical and complex designs, identification of the appropriate level for tests and full reporting of outcomes
☒	☐	Estimates of effect sizes (e.g. Cohen's *d*, Pearson's *r*), indicating how they were calculated

Our web collection on statistics for biologists contains articles on many of the points above.

Software and code

Policy information about availability of computer code

Data collection	Data are presented as the means ± standard deviations (SDs). Software was used for collection and analysis and is listed below.
Data analysis	Excel 2016 and Prism software version 8 (GraphPad Software) were used for data analysis and making figures. CHIP-qPCR was performed using CFX96 Real-Time System (BIO-RAD). The images of immunofluoresence staining was using Leica SP5 and processed by Adobe Photoshop CC2019; flow cytometry analysis was done by FlowJo 7.6 and BD FACSDiva 4.1.

For manuscripts utilizing custom algorithms or software that are central to the research but not yet described in published literature, software must be made available to editors and reviewers. We strongly encourage code deposition in a community repository (e.g. GitHub). See the Nature Research guidelines for submitting code & software for further information.

Data

Policy information about availability of data

All manuscripts must include a data availability statement. This statement should provide the following information, where applicable:

- Accession codes, unique identifiers, or web links for publicly available datasets
- A list of figures that have associated raw data
- A description of any restrictions on data availability

The cDNA array and ChIP- seq data generated in this study have been deposited in the Gene Expression Omnibus (GEO) database under accession code GSE187418 (https://www.ncbi.nlm.nih.gov/geo/query/acc.cgi?acc= GSE187418), GSE196067 (https://www.ncbi.nlm.nih.gov/geo/query/acc.cgi?acc=GSE196067). The data of human genome(hg19) can get from NCBI (https://www.ncbi.nlm.nih.gov/assembly/GCF_000001405.13/). Other data supporting the findings of this study are available from the authors upon reasonable request.

April 2020

Field-specific reporting

Please select the one below that is the best fit for your research. If you are not sure, read the appropriate sections before making your selection.

☒ Life sciences ☐ Behavioural & social sciences ☐ Ecological, evolutionary & environmental sciences

For a reference copy of the document with all sections, see nature.com/documents/nr-reporting-summary-flat.pdf

Life sciences study design

All studies must disclose on these points even when the disclosure is negative.

Sample size	All animal experiments were performed in seven mice each group and studies in vitro were performed in at least three replicated
Data exclusions	For both in vivo and in vitro analyses in this manuscript, we did not exclude any data.
Replication	For in vitro experiments, at least two-three biologically independent experiments were performed unless stated otherwise. As indicated in the figure legends, data are presented as mean ± s.d. of three samples or test, or one representative analysis. ALL attempts of replication were successful.<br>For in vivo experiments, at least 7 animals were performed statistical analysis for survival time, data are presented as mean ± s.d. of 7 animals.
Randomization	In the animal studies, we conducted randomization of the mice by which there was no bias on the averages of tumor sizes among experimental groups prior to treatments. Mice received inoculation of the same batch tumor cells were randomly grouped into experimental groups. Therapeutic evaluations were carried out through a team-based approach, with the reviewer who imaged tumors and recorded survival blinded to the treatment groups. The cells in vitro from each cell line were pooled, and then seeded and treated randomly.
Blinding	Experiments of MTT, Colony formation, western blot, CHIP-qPCR, DNA sequencing, Flow Cytometry, histological staining and mouse survival and tumor imaging were recorded ND performed in a blinded fashion by laboratory members. For Gene expression profile assay, the investigators were not blinded.

Reporting for specific materials, systems and methods

We require information from authors about some types of materials, experimental systems and methods used in many studies. Here, indicate whether each material, system or method listed is relevant to your study. If you are not sure if a list item applies to your research, read the appropriate section before selecting a response.

Materials & experimental systems

n/a	Involved in the study	
☐	☒	Antibodies
☐	☒	Eukaryotic cell lines
☒	☐	Palaeontology and archaeology
☐	☒	Animals and other organisms
☒	☐	Human research participants
☒	☐	Clinical data
☒	☐	Dual use research of concern

Methods

n/a	Involved in the study	
☐	☒	ChIP-seq
☐	☒	Flow cytometry
☒	☐	MRI-based neuroimaging

Antibodies

Antibodies used	We describe the source of the Abs in the supplementary table. Primary antibodies:<br>ZNF117 (Novus NBP1-79242 1:1000)<br>Beta-actin (BioLegend 664802 1:1000)<br>Nestin (BioLegend 841901 1:100)<br>GalC (Sigma MAB342 1:100)<br>GFAP (Dako Z0334 1:100)<br>Tuj1 (R&D Systems MAB1195 1:100)<br>Olig1 (R&D Systems MAB2417 1:100)<br>Secondary antibodies:<br>Alexa Fluor® 555 goat anti-mouse IgG(H+L) (Invitrogen A-21422 1:400)<br>Alexa Fluor® 488 chicken anti-rabbit IgG(H+L) (Invitrogen A-21441 1:800)<br><br>For CHIP-Seq and ChIP-qPCR analysis:<br>ZNF117 (Novus NBP1-79242)
Validation	All antibodies used in this study are commercially available, and all have been validated by the manufactures.

Validation	https://www.novusbio.com/products/znf117-antibody_nbp1-79242<br>https://www.biolegend.com/en-us/search-results?Keywords=actin<br>https://www.labome.com/product/BioLegend/841901.html<br>https://www.sigmaaldrich.com/US/en/product/mm/mab342<br>https://www.agilent.com/en/product/immunohistochemistry/antibodies-controls/primary-antibodies/glial-fibrillary-acidic-protein-(concentrate)-76683<br>https://www.rndsystems.com/cn/products/neuron-specific-beta-iii-tubulin-antibody-tuj-1_mab1195<br>https://www.rndsystems.com/cn/products/human-mouse-olig1-antibody-257219_mab2417<br>https://www.thermofisher.com/antibody/product/Chicken-anti-Rabbit-IgG-H-L-Cross-Adsorbed-Secondary-Antibody-Polyclonal/A-21441<br>https://www.thermofisher.com/antibody/product/Goat-anti-Mouse-IgG-H-L-Cross-Adsorbed-Secondary-Antibody-Polyclonal/A-21422

Eukaryotic cell lines

Policy information about cell lines

Cell line source(s)	HEK293T cells were purchsed from the ATCC. Primary glioblastoma cells, including GS5, PS24, PS24, PS30 were subcultured from human glioblastoma patients from Yale New Haven Hospital.
Authentication	HEK293T cells were purchased from the credible vendors, and they provided the authentication file. Primary glioblastoma cells were authenticated by our lab after subculture. Cell lines obtained from external institutions were authenticated by morphology, phenotype and growth.
Mycoplasma contamination	The cell line was tested for mycoplasma contamination regularly every four weeks using the Mycoplasma Detection Kit (Beyotime, Haimen, China). All cell lines tested negative for mycoplasma.
Commonly misidentified lines<br>(See ICLAC register)	No commonly misidentified cell lines were used.

Animals and other organisms

Policy information about studies involving animals; ARRIVE guidelines recommended for reporting animal research

Laboratory animals	Female nude mice (BALB/c nu/nu, 6 weeks old) were employed. Mice were housed in a standard environment which was characterized by 12 h light/dark cycle, 22-25°C and 40-60% humidity with free access to water and chow.
Wild animals	This study did not involve wild animals.
Field-collected samples	This study did not involve field-collected samples.
Ethics oversight	This study was compliant with all relevant ethical regulations regarding animal research and was approved by the Institutional Animal Care and Use Committee of Yale University.

Note that full information on the approval of the study protocol must also be provided in the manuscript.

ChIP-seq

Data deposition

☒ Confirm that both raw and final processed data have been deposited in a public database such as GEO.

☐ Confirm that you have deposited or provided access to graph files (e.g. BED files) for the called peaks.

Data access links<br>*May remain private before publication.*	https://www.ncbi.nlm.nih.gov/geo/query/acc.cgi?acc=GSE196067
Files in database submission	GS5_IP_vs_In_MACS2_peaks.txt<br>Input_GS5_TGACCA_L007_R1_all_nullsort.bam<br>Input_GS5_TGACCA_L007_R1_all_nullsort.fastq.gz<br>IP_GS5_GCCAAT_L007_R1_all_nullsort.bam<br>IP_GS5_GCCAAT_L007_R1_all_nullsort.fastq.gz
Genome browser session<br>(e.g. UCSC)	https://genome.ucsc.edu/

Methodology

Replicates	ChIP Seq experiments were done in one Biological Replicate. Each each biological replicates had three technical replicates which were pooled before library preparation and sequencing.
Sequencing depth	All Sequencing were done as 50bp single end Sequence. Sequencing were done to achieve 16 million reads per biological replicate .

Antibodies	GS5 cells wwere used for ZNF117 ChIP using anti-ZNF117 antibody (Novus NBP1-79242).
Peak calling parameters	*Specify the command line program and parameters used for read mapping and peak calling, including the ChIP, control and index files used.*
Data quality	QC passed.
Software	FastQC.

Flow Cytometry

Plots

Confirm that:

☒ The axis labels state the marker and fluorochrome used (e.g. CD4-FITC).

☒ The axis scales are clearly visible. Include numbers along axes only for bottom left plot of group (a 'group' is an analysis of identical markers).

☒ All plots are contour plots with outliers or pseudocolor plots.

☒ A numerical value for number of cells or percentage (with statistics) is provided.

Methodology

Sample preparation	Glioblastoma cells were dissociated by accutase into single cell suspension and these cell samples were diluted in 10^8 cells/ml buffer concentrations and stained with cell surface markers.
Instrument	For the data collection, FACS Aria II (BD) was used.
Software	For the data collection, BD FACSDiva software (BD) was used. For the data analysis, FlowJo software was used.
Cell population abundance	No cell sorting was performed.
Gating strategy	For the all data analysis, FSC-A/SSC-A gating was performed for cell size and characteristics determination and exclusion of debris. In addition, FSC-H/FSC-W gating and SSC-H/SSC-W gating were performed for the exclusion of cell doublets. Positive gate and negative gate were determined by the signal intensity. Representative gating results were also described in this article.

☒ Tick this box to confirm that a figure exemplifying the gating strategy is provided in the Supplementary Information.
